# Reduced-frequency bictegravir, emtricitabine, and tenofovir alafenamide in virologically suppressed adults with HIV: a single-centre randomised phase 2 pilot trial in Spain

**DOI:** 10.1016/j.eclinm.2026.104158

**Published:** 2026-08-23

**Authors:** Iván Chivite, Elisa Moraga, Abiu Sempere, Sònia Vicens-Artés, Pilar Callau, Cristina Rovira, Carmen Hurtado, Amedeo De Nicoló, Alejandro Sánchez-Molina, Berta Torres, Jordi Blanch, José Alcamí, José Luis Blanco, Antonio D’Avolio, Sonsoles Sánchez-Palomino, Elisa De Lazzari, Esteban Martínez

**Affiliations:** a Hospital Clínic Barcelona–Institut d’Investigacions Biomèdiques August Pi i Sunyer (IDIBAPS), Barcelona, Spain; b University of Barcelona, Barcelona, Spain; c Ospedale Amedeo di Savoia Hospital, Turin, Italy; d University of Turin, Turin, Italy; e Centro de Investigación Biomédica en Red de Enfermedades Infecciosas (CIBERINFEC), Instituto de Salud Carlos III, Madrid, Spain; f Centro de Investigación Biomédica en Red de Salud Mental (CIBERSAM), Instituto de Salud Carlos III, Madrid, Spain; g Reial Acadèmia de Medicina de Catalunya (RAMC), Barcelona, Spain

**Keywords:** HIV, Bictegravir, Emtricitabine, Tenofovir alafenamide, Reduced-frequency dosing, Pharmacokinetics

## Abstract

**Background:**

Reduced-frequency oral antiretroviral therapy may lessen treatment burden, but the exposure thresholds required to maintain virological suppression and the role of the HIV reservoir remain undefined.

**Methods:**

BETAF-RED was a 48-week, single-centre, randomised, open-label, controlled phase 2 pilot trial conducted at Hospital Clínic, Barcelona, Spain. Eligible participants were adults aged 18 years or older with HIV-1 RNA <50 copies per mL for at least 6 months while receiving once-daily bictegravir, emtricitabine, and tenofovir alafenamide. Key eligibility criteria included stable clinical condition and CD4 count >350 cells per μL; key exclusions included previous virological failure or documented resistance to study drugs, active hepatitis B or C infection, and conditions judged to jeopardise adherence. Participants were assigned 1:1:1:1 to receive the oral fixed-dose single-tablet regimen bictegravir, emtricitabine, and tenofovir alafenamide 50/200/25 mg once daily, three times weekly, twice weekly, or once weekly for 48 weeks. The primary endpoint was maintenance of HIV-1 RNA <50 copies per mL (virological success) at weeks 12 and 48, assessed by Food and Drug Administration Snapshot in the intention-to-treat exposed population, defined as all randomised participants receiving study drug, and in the prespecified on-treatment analysis. Secondary outcomes were virological failure and no virological data. Safety analyses included all participants who received at least one dose of study drug. This was not a non-inferiority study, and no non-inferiority margin was specified. ClinicalTrials.gov, NCT05602506.

**Findings:**

Between November 7, 2022, and July 3, 2023, 40 participants were enrolled; 39 received study medication. In the intention-to-treat exposed Food and Drug Administration Snapshot analysis, virological success was observed at week 12 in 9/9, 9/10, 10/10, and 8/10 participants in the once-daily, three-times-weekly, twice-weekly, and once-weekly arms, respectively. At week 48, virological success was observed in 8/9, 9/10, 9/10, and 7/10 participants in the once-daily, three-times-weekly, twice-weekly, and once-weekly arms, respectively. At week 12, virological failure and no virological data were observed in 0/9 and 0/9, respectively, in the once-daily arm; 1/10 and 0/10, respectively, in the three-times-weekly arm; 0/10 and 0/10, respectively, in the twice-weekly arm; and 2/10 and 0/10, respectively, in the once-weekly arm. At week 48, virological failure and no virological data were observed in 0/9 and 1/9, respectively, in the once-daily arm; 1/10 and 0/10, respectively, in the three-times-weekly arm; 0/10 and 1/10, respectively, in the twice-weekly arm; and 2/10 and 1/10, respectively, in the once-weekly arm. In the prespecified on-treatment analysis, virological success was observed in 36/37 participants at week 12 and in all 33 participants remaining on their assigned regimen at week 48; the only virological failure occurred at week 12 in the three-times-weekly arm, and no virological data were missing at either timepoint. All three participants with protocol-defined confirmed virological failure regained suppression after resuming once-daily therapy, without emergent resistance.

**Interpretation:**

Most participants met the primary endpoint of maintained virological suppression at weeks 12 and 48 in the intention-to-treat exposed FDA Snapshot analysis. Early protocol-defined confirmed virological failures occurred in the once-weekly arm, and one confirmed low-level viraemia event occurred in the three-times-weekly arm. Because the trial was not powered for formal between-arm comparisons, these findings should be interpreted descriptively. Once-weekly dosing is not supported as a maintenance strategy; twice-weekly and three-times-weekly dosing require confirmation in larger, adequately powered studies with close virological monitoring before any clinical role can be considered.

**Funding:**

Instituto de Salud Carlos III and Institut d’Investigacions Biomèdiques August Pi i Sunyer.

**Research in context**

**Evidence before this study**

We searched PubMed from inception up to July 28, 2026, without language restrictions, using the terms “bictegravir”, “emtricitabine”, “tenofovir alafenamide”, “non-daily”, “short-cycle”, “intermittent”, “reduced-frequency”, and “dose reduction”. Observational cohorts from France and Italy have reported durable virological suppression with reduced-frequency bictegravir, emtricitabine, and tenofovir alafenamide, most commonly administered four or five days per week. A randomised trial evaluating a five-days-on/two-days-off short-cycle strategy showed comparable 48-week efficacy and safety to standard once-daily therapy. However, no controlled trial had evaluated fixed reduced-frequency schedules of bictegravir, emtricitabine, and tenofovir alafenamide as infrequent as three-times-weekly, twice-weekly, or once-weekly dosing. In addition, the pharmacological limits of dosing forgiveness and the potential contribution of HIV reservoir characteristics to virological control under markedly reduced drug exposure remained undefined.

**Added value of this study**

BETAF-RED is a randomised, open-label, phase 2 pilot trial evaluating fixed three-times-weekly, twice-weekly, and once-weekly bictegravir, emtricitabine, and tenofovir alafenamide schedules in adults with sustained virological suppression, under close trial monitoring. In the intention-to-treat exposed Food and Drug Administration Snapshot analysis, early protocol-defined confirmed virological failures occurred in the once-weekly arm. In the prespecified on-treatment analysis, the only virological failure at week 12 occurred in the three-times-weekly arm, and no virological failures occurred at week 48 in any arm. Twice-weekly and three-times-weekly dosing showed exploratory signals of maintained suppression in most participants, but the trial was not designed to establish definitive efficacy, formally compare reduced-frequency schedules, or select regimens for clinical implementation. No emergent resistance mutations were detected in resistance testing performed at the time of protocol-defined confirmed virological failure; all affected participants subsequently regained suppression after resuming once-daily therapy. Plasma and intracellular pharmacokinetic data supported a concentration–viraemia relationship, and HIV reservoir measures remained stable overall. The two early failures in the once-weekly arm occurred in participants with among the highest baseline intact proviral DNA and intracellular HIV RNA transcriptional activity within that arm, suggesting that reservoir burden or transcriptional activity might influence rebound risk under marked dose reduction.

**Implications of all the available evidence**

Available evidence supports the biological and pharmacological plausibility of reduced-frequency oral antiretroviral therapy in carefully selected, virologically suppressed individuals. In the BETAF-RED trial, once-weekly dosing of bictegravir, emtricitabine, and tenofovir alafenamide was associated with early protocol-defined confirmed virological failures. These findings do not support once-weekly bictegravir, emtricitabine, and tenofovir alafenamide as a maintenance switch strategy. Twice-weekly and three-times-weekly dosing remain investigational and may warrant further evaluation in larger, adequately powered, multicentre trials with more diverse populations, longer follow-up, close viral-load monitoring, pharmacokinetic assessment, and prespecified evaluation of reservoir-related predictors of rebound. Future studies should also address generalisability, hepatitis B virus safety, transmission-related considerations in the event of detectable viraemia, and strategies for prompt return to fully suppressive once-daily therapy if virological rebound is confirmed.

## Introduction

Modern antiretroviral therapy achieves durable viral suppression and near-normal life expectancy in people living with HIV.^1^ Lifelong treatment is still required, and cumulative drug exposure may contribute to long-term toxicity and treatment burden.^2^ Treatment preferences increasingly emphasise convenience and reduced pill burden, supporting interest in simplified oral strategies and long-acting injectables.^3^^,^^4^ Although long-acting cabotegravir and rilpivirine have expanded maintenance treatment options, implementation remains challenging in many settings.^4^ Pharmacologically guided oral simplification may therefore offer a complementary approach to reducing dosing frequency and cumulative drug exposure.

Previous studies have shown that short-cycle or intermittent regimens can maintain virological suppression in selected adherent adults receiving high-barrier antiretroviral therapies, including integrase inhibitors and boosted protease inhibitors.^5^^,^^6^ The ANRS QUATUOR trial demonstrated non-inferior efficacy of a four-days-on and three-days-off regimen compared with once-daily therapy, and smaller studies of bictegravir, emtricitabine, and tenofovir alafenamide (B/F/TAF) have supported the feasibility of five-days-on/two-days-off or three-times-weekly dosing.^7^^,^^8^ These studies suggest substantial pharmacological forgiveness, but the lower exposure limit compatible with sustained viral control remains undefined. Bictegravir's slow dissociation from the HIV-1 integrase–DNA complex, together with the long intracellular half-lives of tenofovir-diphosphate and emtricitabine-triphosphate, provides a rationale for studying reduced-frequency dosing of this regimen.^9^, ^10^, ^11^

We designed BETAF-RED to explore whether reduced-frequency B/F/TAF could maintain virological suppression in selected adults already suppressed on once-daily B/F/TAF, and to generate preliminary virological, pharmacokinetic, and reservoir data.

## Methods

### Study design and participants

BETAF-RED was a 48-week, randomised, open-label, controlled, single-centre phase 2 pilot trial conducted at Hospital Clínic, a tertiary university hospital in Barcelona, Spain. Participants were randomly assigned to continue once-daily B/F/TAF or to switch to one of three fixed reduced-frequency schedules: three-times weekly, twice weekly, or once weekly. The trial was designed to generate preliminary clinical, virological, and pharmacokinetic data; it was not designed to establish definitive efficacy, test non-inferiority, formally compare reduced-frequency arms, or select regimens for clinical implementation. The trial was registered in EudraCT (2022-000358-26) and ClinicalTrials.gov (NCT05602506). The protocol (version 1.1, April 10, 2022) is provided in the Appendix.

Eligible participants were adults with HIV-1 infection, plasma HIV-1 RNA < 50 copies per mL for at least 6 months while receiving once-daily B/F/TAF, stable clinical condition, and CD4 count >350 cells per μL. Exclusion criteria included prior virological failure or documented resistance to bictegravir, emtricitabine, or tenofovir; diagnosed psychiatric illness; current or historical alcohol abuse or illicit drug use; active hepatitis B or C infection; and any condition judged to jeopardise adherence. Participants of childbearing potential required a negative pregnancy test and effective contraception during the study. Sex was recorded in the electronic case report form from medical records using male and female categories. Gender identity, race, and ethnicity were not collected as separate variables because they were not prespecified in the protocol or case report form.

### Ethics

The study was approved by the Comité de Ética de la Investigación con medicamentos del Hospital Clínic de Barcelona (approval reference HCB/2022/0285), which issued a favourable opinion on April 28, 2022. The ethics committee reviewed the protocol, participant information sheet, informed consent form, recruitment plan, participant compensation and indemnity arrangements, personal-data handling procedures, future use of biological samples, and the suitability of the participating centre and investigator. The trial was subsequently authorised by the Spanish Agency for Medicines and Medical Devices (Agencia Española de Medicamentos y Productos Sanitarios [AEMPS]) on May 11, 2022. All participants provided written informed consent before any study procedure.

### Randomisation and masking

Participants were randomly assigned 1:1:1:1 to continue once-daily (OD) B/F/TAF or to receive B/F/TAF three times weekly (3 W; Monday, Wednesday, Friday), twice weekly (2 W; Tuesday, Friday), or once weekly (1 W; Wednesday) for 48 weeks. Randomisation used a computer-generated allocation list with randomly varied block sizes of 2–4, prepared by the study statistician and implemented in the electronic case report form. Allocation was revealed only after eligibility and enrolment were confirmed. Participants and treating clinicians were not masked, but laboratory, pharmacokinetic, and imaging personnel remained blinded to treatment allocation.

### Procedures

All participants received B/F/TAF as the fixed-dose single-tablet regimen bictegravir/emtricitabine/tenofovir alafenamide 50/200/25 mg (Biktarvy; Gilead Sciences, Foster City, CA, USA). Study visits occurred at baseline and weeks 4, 12, 24, 36, and 48. Pharmacokinetic visits were scheduled in the morning at trough, immediately before the next scheduled dose. When dosing intervals varied within a regimen, sampling was planned after the longest interval between doses to capture the lowest expected trough exposure.

At each visit, investigators recorded medical history, concomitant medications, vital signs, and safety laboratories. Concomitant medications were prospectively reviewed at each visit for potential interactions with B/F/TAF, particularly medications or supplements that could reduce study-drug exposure through enzyme induction or polyvalent-cation chelation. Baseline hepatitis B virus (HBV) serology included HBsAg, anti-HBs, and anti-HBc, obtained from screening assessments or medical records. HBV DNA testing was used to exclude active HBV infection when clinically indicated by serology, but follow-up HBV DNA and repeat HBsAg testing were not protocol-mandated. ALT and AST were monitored at scheduled visits. Adherence counselling was reinforced at every visit, and adherence was assessed by pill counts and the Simplified Medication Adherence Questionnaire (SMAQ).^12^ Pregnancy testing in urine or blood was performed in participants of childbearing potential at protocol-specified visits. Participants were counselled on safer sex and advised to avoid unprotected sex during the study.

Plasma HIV-1 RNA (limit of detection 50 copies per mL) was measured at all visits. Protocol-defined confirmed virological failure was defined as two consecutive HIV-1 RNA values ≥50 copies per mL, with confirmatory testing performed as an unscheduled test within the protocol-specified 2-week window after the first detectable result. This conservative threshold was prespecified as an early safety criterion to trigger prompt return to once-daily B/F/TAF and should not be interpreted as equivalent to routine-care definitions of virological failure. Low-level viraemia was used descriptively to denote confirmed or persistent HIV-1 RNA ≥50 and <200 copies per mL; this was not a prespecified endpoint. Participants with protocol-defined confirmed virological failure resumed once-daily B/F/TAF unless resistance testing indicated the need for an optimised regimen.

A prespecified stopping rule required discontinuation of any reduced-frequency arm if both criteria were met: more than 30% of participants experienced on-treatment protocol-defined confirmed virological failure and more than 10% met this criterion in the ITT-exposed population. The >10% ITT-exposed criterion alone was not sufficient to stop an arm. Participants with protocol-defined confirmed virological failure discontinued the assigned reduced-frequency regimen and restarted once-daily B/F/TAF promptly, while remaining in follow-up whenever possible. Once all participants reached the week-12 primary endpoint, an independent Data Safety Monitoring Board reviewed safety and efficacy data and identified no safety concerns. The study would be terminated if all three reduced-frequency arms required discontinuation.

Ultrasensitive plasma HIV-1 RNA (limit of detection 5 copies per mL) was measured at baseline and week 48. The HIV reservoir was quantified in peripheral-blood CD4^+^ T cells at baseline and week 48 by droplet digital PCR using the Intact Proviral DNA Assay for total, intact, and defective HIV DNA,^13^ and quantification of intracellular HIV RNA species (LongLTR, Pol, PolyA, TatRev),^14^ expressed as copies per 10^6^ CD4^+^ T cells. CD4^+^ and CD8^+^ cell counts and the CD4^+^/CD8^+^ ratio were measured at every visit. High-sensitivity C-reactive protein and interleukin-6 were measured by ELISA at baseline and week 48. Adverse events were collected at each visit, recorded using standard medical terminology, and graded according to the Division of AIDS Table for Grading the Severity of Adult and Paediatric Adverse Events, corrected version 2.1. DXA was performed at baseline and week 48 to assess body composition; total body fat percentage was prespecified as the main body-composition outcome. Patient-reported outcomes included the Pittsburgh Sleep Quality Index^15^ and EQ-5D-5L.^16^

Plasma and peripheral blood mononuclear cells (PBMCs) pharmacokinetic sampling was performed at baseline, week 12, and week 48 to measure trough bictegravir, emtricitabine, and tenofovir in plasma, and intracellular bictegravir, emtricitabine triphosphate, and tenofovir diphosphate in PBMCs. No formal correction or imputation for sampling-time deviations was applied; pharmacokinetic concentrations were analysed as observed in descriptive and exploratory models. Quantification used validated LC–MS/MS or UHPLC–MS/MS methods.^17^, ^18^, ^19^, ^20^ Intracellular phosphorylated NRTI metabolites were normalised to cell number and molar mass and expressed as fmol per 10^6^ cells.^17^ Lower limits of quantification were 39 ng/mL in plasma and 0.39 ng/sample in PBMCs for bictegravir; 1.6 ng/mL and 0.039 ng/sample for TFV-DP; and 9.8 ng/mL and 0.039 ng/sample for FTC-TP. Corresponding intracellular LLOQs were 8.6 fmol/10^6^ cells for TFV-DP and 7.9 fmol/10^6^ cells for FTC-TP. Trough concentrations were interpreted relative to exploratory pharmacological reference values, not validated clinical treatment targets. For bictegravir, the protein-adjusted IC95 of 162 ng/mL was used as a conservative plasma reference; its application to intracellular PBMC concentrations was descriptive. For intracellular TFV-DP, reference values were extrapolated from pharmacodynamic studies suggesting EC50 values of approximately 75–125 fmol/10^6^ cells and an approximate EC90 of 290 fmol/10^6^ cells.^21^ The 290 fmol/10^6^ cells value was used as a conservative exploratory reference for higher intracellular TFV-DP exposure, not as a validated treatment target.

### Outcomes

The primary endpoint was maintenance of plasma HIV-1 RNA <50 copies per mL at weeks 12 and 48, assessed using the Food and Drug Administration (FDA) Snapshot algorithm in the intention-to-treat exposed (ITT-exposed) population and in the prespecified on-treatment population, as specified in the protocol. The ITT-exposed analysis was interpreted as the conservative treatment-strategy analysis; the on-treatment analysis described outcomes while participants remained on the assigned regimen.

Secondary and exploratory endpoints included protocol-specified virological endpoints, immunological safety markers, subclinical-toxicity endpoints, pharmacokinetic endpoints, safety outcomes, and patient-reported outcomes. Virological endpoints included plasma HIV-1 RNA < 50 copies per mL at weeks 4, 24, and 36; viral blips, defined as a single HIV-1 RNA value ≥ 50 copies per mL followed by resuppression to <50 copies per mL without treatment modification; and ultrasensitive plasma HIV-1 RNA < 5 copies per mL at week 48. Genotypic resistance testing in participants with protocol-defined confirmed virological failure was prespecified as exploratory. Reservoir endpoints included total, intact, and defective HIV DNA by Intact Proviral DNA Assay (IPDA) and intracellular HIV RNA species by droplet digital PCR. Immunological safety markers included CD4^+^ and CD8^+^ cell counts, CD4^+^/CD8^+^ ratio, and inflammatory biomarkers. Protocol-specified subclinical-toxicity and safety outcomes included adverse events, weight, body mass index, and DXA-derived body fat percentage. Protocol-specified patient-reported exploratory endpoints included sleep quality assessed with the Pittsburgh Sleep Quality Index and health-related quality of life assessed with EQ-5D-5L. Protocol-specified pharmacokinetic exploratory endpoints included plasma bictegravir, emtricitabine, and tenofovir concentrations, and intracellular bictegravir, emtricitabine-triphosphate, and tenofovir-diphosphate concentrations. Post hoc descriptive analyses included laboratory abnormalities, including grade 2 or higher ALT or AST elevations and declines of at least 25% in estimated glomerular filtration rate. Any patient-reported analyses beyond the protocol-specified Pittsburgh Sleep Quality Index and EQ-5D-5L measures were considered post hoc descriptive analyses. Receiver operating characteristic analyses evaluating plasma bictegravir and intracellular bictegravir, emtricitabine-triphosphate, and tenofovir-diphosphate concentrations as continuous predictors of detectable viraemia were post hoc exploratory analyses.

### Statistical analysis

The target sample size was 40 participants, with 10 per group. Because this was a pilot study designed to explore feasibility and biological signals of reduced-frequency dosing, no formal power calculation was performed and all analyses were considered exploratory.

The ITT-exposed population included all randomised participants who received at least one dose of study drug. The on-treatment population included data collected while participants remained on their assigned regimen without major protocol deviations.

For the primary endpoint, outcomes at weeks 12 and 48 were summarised by treatment arm using the FDA Snapshot algorithm. In the ITT-exposed FDA Snapshot analysis, participants were classified as virological success, virological failure, or no virological data. Participants with HIV-1 RNA <50 copies per mL in the relevant window were classified as successes. Participants who discontinued the assigned regimen or resumed once-daily B/F/TAF because of protocol-defined confirmed virological failure or lack/loss of efficacy were classified in the FDA Snapshot virological failure category at the relevant and subsequent timepoints, irrespective of subsequent resuppression. Participants without HIV-1 RNA data in the relevant window and without prior protocol-defined confirmed virological failure or lack/loss of efficacy were classified as having no virological data. No imputation of missing HIV-1 RNA values was performed. In on-treatment analyses, participants who changed or discontinued the assigned regimen were not considered on treatment after that timepoint. Proportions were reported with exact 95% confidence intervals using the Clopper–Pearson method.

For outcomes other than the FDA Snapshot primary endpoint, analyses used available observed data, with no imputation and no last-observation-carried-forward approach. Longitudinal continuous outcomes were analysed using mixed-effects linear regression models including treatment arm, visit, and their interaction, with random effects at the participant level. Skewed variables, including pharmacokinetic parameters, reservoir measures, and inflammatory biomarkers, were log10-transformed. Binary outcomes were analysed using mixed-effects Poisson regression models. Denominators for binary or categorical outcomes reflected the number of participants with available data at each visit. Safety analyses included all participants who received at least one dose of study medication, with adverse events summarised according to observed follow-up. Adverse-event incidence rates were reported per 100 person-years with 95% confidence intervals and compared using Poisson regression. Baseline characteristics were summarised descriptively, without formal statistical comparisons.

As a post hoc exploratory pharmacokinetic–virological analysis, receiver operating characteristic curves were used to assess plasma bictegravir and intracellular bictegravir, FTC-TP, and TFV-DP concentrations as continuous predictors of detectable viraemia, defined as HIV-1 RNA ≥50 copies per mL. Matched concentration–HIV-1 RNA pairs from available study timepoints were pooled across participants and visits. Because few detectable viraemia events occurred and repeated observations from the same participants were pooled, these analyses were considered descriptive and hypothesis-generating. No pharmacokinetic threshold derived from these analyses was interpreted as a validated clinical cutoff. No prespecified subgroup or sensitivity analyses were planned. Analyses were performed using Stata version 19 (StataCorp LLC, College Station, TX, USA). Data were collected in REDCap, hosted at Hospital Clínic, Barcelona, Spain.

### Role of the funding source

The funders had no role in study design, data collection, analysis, interpretation, manuscript writing, or the decision to submit for publication.

## Results

**Fig. 1:**
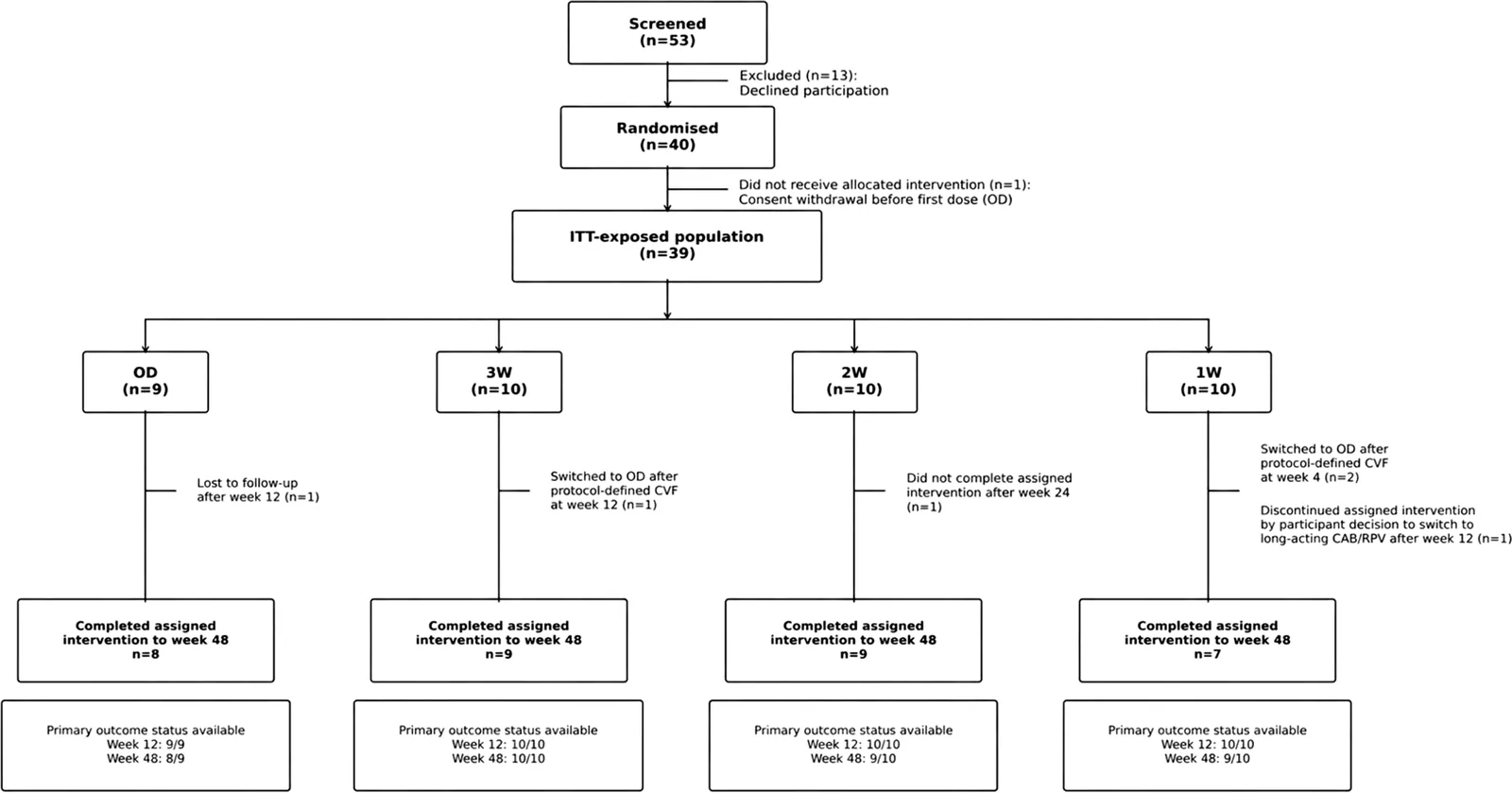
Study profile fifty-three individuals were screened; 13 declined participation. Forty participants were randomly assigned in a 1:1:1:1 ratio to the once-daily (OD), three-times-weekly (3 W), twice-weekly (2 W), or once-weekly (1 W) arms. One participant assigned to the OD arm withdrew consent before receiving the first dose and was not included in the ITT-exposed population. Six participants did not complete the assigned intervention through week 48. In the OD arm, one participant was lost to follow-up or transferred care after week 12. In the 3 W arm, one participant resumed once-daily B/F/TAF after protocol-defined confirmed virological failure at week 12. In the 2 W arm, one participant did not complete the assigned intervention after week 24; the reason was not documented in the analysis dataset. In the 1 W arm, two participants resumed once-daily B/F/TAF after protocol-defined confirmed virological failure at week 4, and one participant discontinued the assigned intervention by participant decision to switch to long-acting CAB/RPV after week 12. OD, once daily; 3 W, three times weekly; 2 W, twice weekly; 1 W, once weekly; ITT-exposed, intention-to-treat exposed; CVF, protocol-defined confirmed virological failure; B/F/TAF, bictegravir, emtricitabine, and tenofovir alafenamide; CAB/RPV, cabotegravir/rilpivirine.

Between November 7, 2022 and July 3, 2023, 53 individuals were screened and 40 were randomised equally to once-daily (OD), three-times-weekly (3 W), twice-weekly (2 W), or once-weekly (1 W) dosing. Thirty-nine participants received at least one dose of study medication and constituted the ITT-exposed population; one participant in the OD arm withdrew consent before the first dose. Thirty-three participants completed the assigned intervention through week 48. Six did not: one in the OD arm was lost to follow-up or transferred care after week 12; one in the 3 W arm switched to OD after protocol-defined confirmed virological failure at week 12; one in the 2 W arm did not complete the assigned intervention after week 24, with the reason not documented in the analysis dataset; and three in the 1 W arm did not complete the assigned intervention, two after protocol-defined confirmed virological failure at week 4 and one by participant decision to switch to long-acting cabotegravir and rilpivirine after week 12 (Fig. 1).

Baseline characteristics are shown in Table 1. Median age was 40 years (IQR 33–46), 36 participants (92%) were men, median time since HIV diagnosis was 8 years (IQR 5–13), median CD4^+^ count was 635 cells per μL (IQR 517–762), median CD4^+^/CD8^+^ ratio was 0.98 (IQR 0.83–1.14), and median body mass index was 24.5 kg/m^2^ (IQR 22.7–27.7). HBV serology was available for 38 participants. Seven (18%) had resolved HBV infection, defined as HBsAg negative, anti-HBs positive, and anti-HBc positive: 3/9 in the OD arm, 2/10 in the 3 W arm, 1/10 in the 2 W arm, and 1/9 in the 1 W arm. No participant with available baseline HBV serology was HBsAg positive.

**Table 1:** Baseline characteristics of study participants, by treatment group.

	OD (n = 9)	3 W (n = 10)	2 W (n = 10)	1 W (n = 10)	Total (n = 39)
Age, years	37 (33–41)	44 (33–47)	43 (37–49)	34 (30–46)	40 (33–46)
Male sex, n (%)	9 (100%)	10 (100%)	8 (80%)	9 (90%)	36 (92%)
Female sex, n (%)	0 (0%)	0 (0%)	2 (20%)	1 (10%)	3 (8%)
Years since HIV diagnosis, years	8 (6–12)	9 (5–16)	9 (7–12)	5 (3–9)	8 (5–13)
Resolved HBV infection, n/N (%)	3/9 (33%)	2/10 (20%)	1/10 (10%)	1/9 (11%)	7/38 (18%)
Weight, kg	73 (67–83)	75 (70–81)	76 (73–81)	77 (75–84)	76 (72–81)
BMI, kg/m^2^	24.1 (21.5–28.8)	24.1 (23.2–26.2)	24.7 (23.0–27.9)	25.2 (21.8–30.4)	24.5 (22.7–27.7)
CD4 count, cells/μL	635 (592–799)	526 (478–706)	629 (546–731)	689 (538–762)	635 (517–762)
CD8 count, cells/μL	723 (534–854)	667 (458–959)	776 (671–1037)	638 (448–890)	714 (494–918)
CD4/CD8 ratio	0.94 (0.91–1.19)	1.01 (0.87–1.13)	0.95 (0.62–1.01)	0.98 (0.94–1.14)	0.98 (0.83–1.14)
Creatinine, mg/dL	1.06 (1.00–1.16)	0.93 (0.82–0.97)	0.92 (0.86–1.05)	0.98 (0.76–1.08)	0.95 (0.86–1.08)
CKD-EPI, mL/min/1.73 m^2^	105 (92–115)	92 (91–110)	93 (85–110)	94 (86–110)	98 (86–115)
Glucose, mg/dL	81 (72–92)	77 (68–85)	82 (79–84)	79 (78–84)	81 (73–85)
Total cholesterol, mg/dL	158 (151–171)	174 (144–204)	186 (151–206)	176 (169–216)	172 (151–204)
LDL cholesterol, mg/dL	100 (88–108)	88 (74–108)	117 (94–137)	105 (86–140)	102 (80–119)
HDL cholesterol, mg/dL	39 (34–47)	43 (40–54)	46 (39–51)	49 (32–53)	43 (37–52)
Triglycerides, mg/dL	111 (97–129)	128 (106–241)	113 (84–131)	119 (75–144)	122 (84–144)
AST, U/L	28 (26–42)	28 (21–42)	23 (17–26)	27 (24–31)	26 (23–32)
ALT, U/L	30 (29–40)	27 (22–54)	25 (21–26)	28 (21–32)	26 (22–33)
Alkaline phosphatase, U/L	76 (66–86)	80 (60–83)	94 (73–102)	77 (69–93)	79 (69–93)
Total bilirubin, mg/dL	0.8 (0.7–0.9)	0.6 (0.5–0.7)	0.65 (0.5–0.9)	0.75 (0.5–0.9)	0.7 (0.5–0.9)

Data are presented as median (IQR) unless otherwise specified. Categorical variables are presented as n (%). For resolved hepatitis B virus (HBV) infection, data are presented as n/N (%) because HBV serology was available for 38/39 participants. Resolved HBV infection was defined as hepatitis B surface antigen (HBsAg) negative, antibody to hepatitis B surface antigen (anti-HBs) positive, and antibody to hepatitis B core antigen (anti-HBc) positive. No participant with available baseline HBV serology was HBsAg positive.

OD = once daily; 3 W = three times weekly; 2 W = twice weekly; 1 W = once weekly; BMI = body mass index; CKD-EPI = estimated glomerular filtration rate calculated using the Chronic Kidney Disease Epidemiology Collaboration equation; CD4/CD8 ratio = ratio of absolute CD4 and CD8 T-cell counts; HDL = high-density lipoprotein; LDL = low-density lipoprotein; AST = aspartate aminotransferase; ALT = alanine aminotransferase; HBV = hepatitis B virus; HBsAg = hepatitis B surface antigen; anti-HBs = antibody to hepatitis B surface antigen; anti-HBc = antibody to hepatitis B core antigen.

**Fig. 2:**
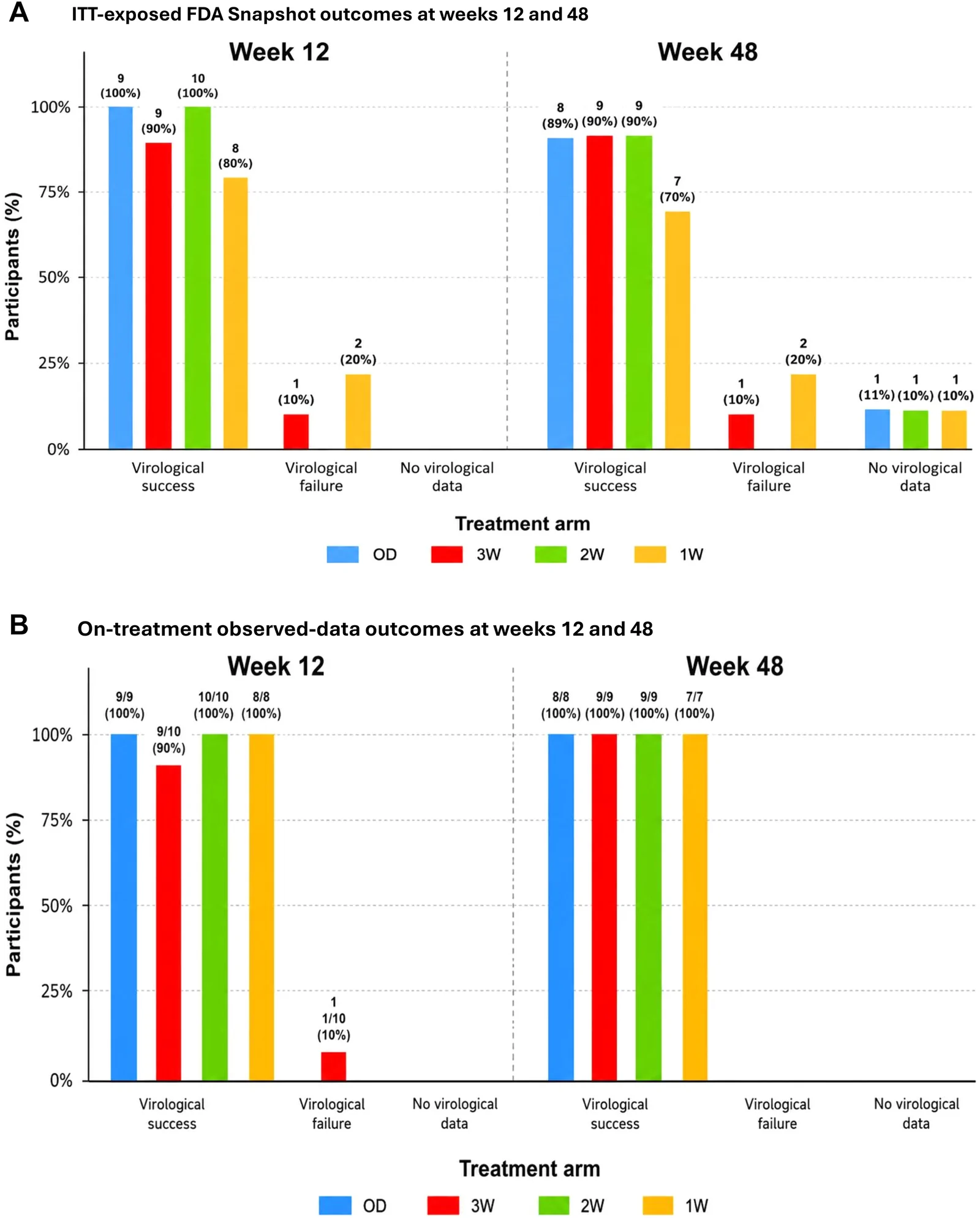
(A) ITT-exposed FDA Snapshot outcomes at weeks 12 and 48 Bars show the percentage of participants classified as virological success, virological failure, or no virological data at each timepoint, by treatment arm. Labels above bars show n (%). In this ITT-exposed FDA Snapshot analysis, participants who discontinued the assigned regimen or resumed once-daily B/F/TAF because of protocol-defined confirmed virological failure or lack/loss of efficacy were classified in the virological failure category at the relevant and subsequent timepoints, irrespective of subsequent resuppression. Colours indicate treatment arm: OD = once daily; 3 W = three times weekly; 2 W = twice weekly; 1 W = once weekly. ITT-exposed = intention-to-treat exposed; B/F/TAF = bictegravir, emtricitabine, and tenofovir alafenamide. (B) On-treatment observed-data outcomes at weeks 12 and 48 Bars show the percentage of participants classified as virological success, virological failure, or no virological data while participants remained on their assigned regimen. Labels above bars show n/N (%). Participants who resumed once-daily B/F/TAF after protocol-defined confirmed virological failure or discontinued the assigned regimen for other reasons were not considered on treatment after that timepoint. Colours indicate treatment arm: OD = once daily; 3 W = three times weekly; 2 W = twice weekly; 1 W = once weekly. ITT-exposed = intention-to-treat exposed; B/F/TAF = bictegravir, emtricitabine, and tenofovir alafenamide.

The ITT-exposed FDA Snapshot outcomes are shown in Table 2 and Fig. 2A, with virological success, virological failure, and no virological data presented separately by treatment arm. At week 12, virological success was observed in 36/39 participants: 9/9 in the OD arm, 9/10 in the 3 W arm, 10/10 in the 2 W arm, and 8/10 in the 1 W arm. Three participants were classified in the FDA Snapshot virological failure category at week 12, two in the 1 W arm and one in the 3 W arm; no participant had missing virological data. At week 48, virological success was observed in 33/39 participants: 8/9 in the OD arm, 9/10 in the 3 W arm, 9/10 in the 2 W arm, and 7/10 in the 1 W arm. Three participants were classified in the FDA Snapshot virological failure category, two in the 1 W arm and one in the 3 W arm, and three had no virological data: one in the OD arm, one in the 2 W arm, and one in the 1 W arm.

**Table 2:** Virological outcomes at weeks 12 and 48 in the ITT-exposed FDA Snapshot and on-treatment observed-data analyses.

Panel A. ITT-exposed population	OD (n = 9)	3 W (n = 10)	2 W (n = 10)	1 W (n = 10)	Total (n = 39)
Week 12					
Virological success	9 (100%)	9 (90%)	10 (100%)	8 (80%)	36 (92%)
Virological failure	0 (0%)	1 (10%)	0 (0%)	2 (20%)	3 (8%)
No virological data	0 (0%)	0 (0%)	0 (0%)	0 (0%)	0 (0%)
Week 48					
Virological success	8 (89%)	9 (90%)	9 (90%)	7 (70%)	33 (85%)
Virological failure	0 (0%)	1 (10%)	0 (0%)	2 (20%)	3 (8%)
No virological data	1 (11%)	0 (0%)	1 (10%)	1 (10%)	3 (8%)

Panel B. On-treatment observed-data population	OD	3 W	2 W	1 W	Total
Week 12					
Virological success	9/9 (100%)	9/10 (90%)	10/10 (100%)	8/8 (100%)	36/37 (97%)
Virological failure^a^	0/9 (0%)	1/10 (10%)	0/10 (0%)	0/8 (0%)	1/37 (3%)
No virological data	0/9 (0%)	0/10 (0%)	0/10 (0%)	0/8 (0%)	0/37 (0%)
Week 48					
Virological success	8/8 (100%)	9/9 (100%)	9/9 (100%)	7/7 (100%)	33/33 (100%)
Virological failure^a^	0/8 (0%)	0/9 (0%)	0/9 (0%)	0/7 (0%)	0/33 (0%)
No virological data	0/8 (0%)	0/9 (0%)	0/9 (0%)	0/7 (0%)	0/33 (0%)

Data are n (%) for Panel A and n/N (%) for Panel B. Panel A shows the intention-to-treat exposed (ITT-exposed) Food and Drug Administration (FDA) Snapshot analysis. Participants who discontinued the assigned regimen or resumed once-daily bictegravir, emtricitabine, and tenofovir alafenamide (B/F/TAF) because of protocol-defined confirmed virological failure or lack/loss of efficacy were classified in the FDA Snapshot virological failure category at weeks 12 and 48, irrespective of subsequent resuppression after restarting once-daily therapy. Participants without HIV-1 RNA data in the relevant analysis window and without prior protocol-defined confirmed virological failure or lack/loss of efficacy were classified as having no virological data.

Panel B shows the on-treatment observed-data analysis. Outcomes were summarised using observed data while participants remained on the assigned regimen. Participants with protocol-defined confirmed virological failure were classified in the virological failure category in the relevant analysis window and were not considered on treatment at subsequent timepoints after resuming once-daily therapy. Participants who changed the assigned regimen or discontinued the assigned intervention for other reasons were not considered on treatment after that timepoint. Therefore, denominators differ between the ITT-exposed and on-treatment analyses.

In Panel A, virological failure denotes the FDA Snapshot analysis category and should not necessarily be interpreted as clinical treatment failure according to routine-care thresholds. The participant in the 3 W arm classified in the virological failure category had confirmed low-level viraemia, with HIV-1 RNA values of 55 and 60 copies per mL, meeting the protocol-defined confirmed virological failure criterion; both values were <200 copies per mL.

OD = once daily; 3 W = three times weekly; 2 W = twice weekly; 1 W = once weekly; ITT-exposed = intention-to-treat exposed; FDA = Food and Drug Administration; B/F/TAF = bictegravir, emtricitabine, and tenofovir alafenamide.

a In Panel B, virological failure refers to protocol-defined confirmed virological failure occurring while the participant remained on the assigned regimen.

The three participants classified in the FDA Snapshot virological failure category met the protocol-defined confirmed virological failure criterion during follow-up: two in the 1 W arm at week 4 and one in the 3 W arm at week 12. The two 1 W events exceeded the >10% ITT-exposed component of the prespecified stopping rule, but the on-treatment component was 2/10 (20%) at week 4 and did not exceed the >30% threshold; therefore, the composite arm-level stopping rule was not formally met. The 3 W event corresponded to confirmed low-level viraemia, with HIV-1 RNA values of 55 and 60 copies per mL, both below 200 copies per mL. All three participants restarted once-daily B/F/TAF, regained viral suppression, and had no emergent resistance mutations. In the ITT-exposed FDA Snapshot analysis, they remained classified as FDA Snapshot virological failures at weeks 12 and 48 because the assigned reduced-frequency regimen had been discontinued for protocol-defined lack/loss of efficacy. Participant-level details are shown in Supplementary Table S1.

The on-treatment observed-data outcomes are shown in Table 2 and Fig. 2B. At week 12, virological success was observed in 36/37 participants: 9/9 in the OD arm, 9/10 in the 3 W arm, 10/10 in the 2 W arm, and 8/8 in the 1 W arm. The only on-treatment protocol-defined confirmed virological failure at week 12 occurred in the 3 W arm and consisted of confirmed low-level viraemia. At week 48, virological success was observed in all 33 participants who remained on the assigned regimen: 8/8 in the OD arm, 9/9 in the 3 W arm, 9/9 in the 2 W arm, and 7/7 in the 1 W arm, with no virological failures and no missing virological data. Viral blips were infrequent, transient, and not associated with protocol-defined confirmed virological failure or treatment discontinuation (Supplementary Table S2).

**Fig. 3:**
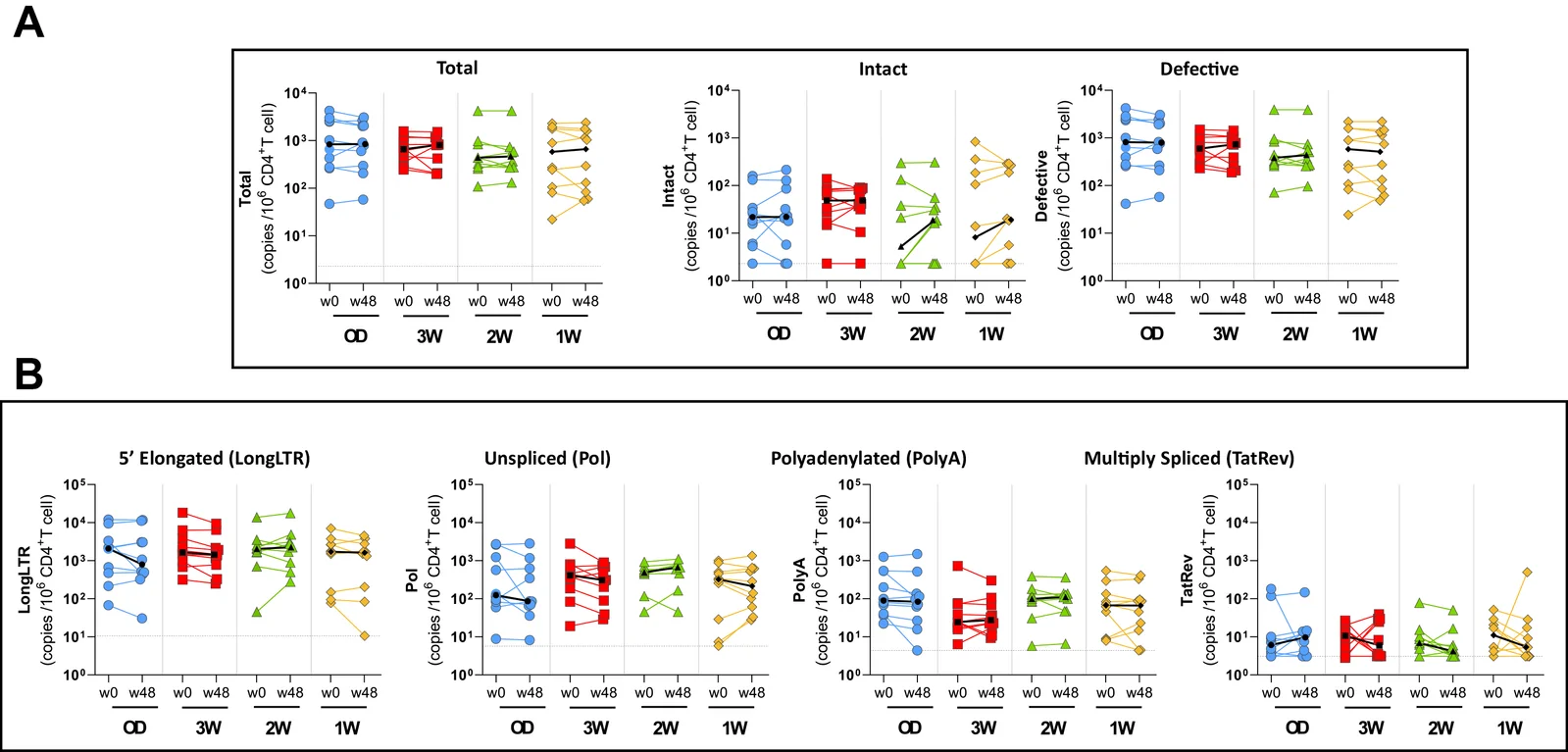
HIV reservoir measures at baseline and week 48 (A) Total, intact, and defective HIV DNA quantified by the Intact Proviral DNA Assay (IPDA), expressed as copies per 106 CD4+ T cells. (B) Intracellular HIV RNA transcripts (LongLTR, Pol, PolyA, TatRev), expressed as copies per 106 CD4+ T cells. Limits of detection from each technique are represented by horizontal discontinuous grey lines. Each dot represents the samples from each study participant and black dots are medians from each arm. OD = once daily; 3 W = three times weekly; 2 W = twice weekly; 1 W = once weekly; w0 = baseline; w48 = week 48.

Ultrasensitive plasma HIV-1 RNA remained stable from baseline to week 48, with no consistent differences between dosing arms (Supplementary Fig. S1A and S1B). Total, intact, and defective HIV proviral DNA also remained stable (Fig. 3A), as did intracellular HIV RNA transcripts (LongLTR, Pol, PolyA, TatRev) (Fig. 3B). The two participants with early protocol-defined confirmed virological failure in the 1 W arm were among those with the highest baseline intact proviral DNA across arms (836 and 357 copies/10^6^ CD4^+^ T cells; overall median 21 copies/10^6^ CD4^+^ T cells) and LongLTR icRNA levels within the 1 W arm (3788 and 7040 copies/10^6^ CD4^+^ T cells; 1 W median 1726 copies/10^6^ CD4^+^ T cells).

CD4^+^ and CD8^+^ T-cell counts remained stable throughout follow-up, with no significant between-arm differences in longitudinal models. The CD4^+^/CD8^+^ ratio increased modestly in the OD and 3 W arms, with mean changes of +0.20 and +0.14, respectively, but between-arm trajectories did not differ significantly (Supplementary Table S3).

**Fig. 4:**
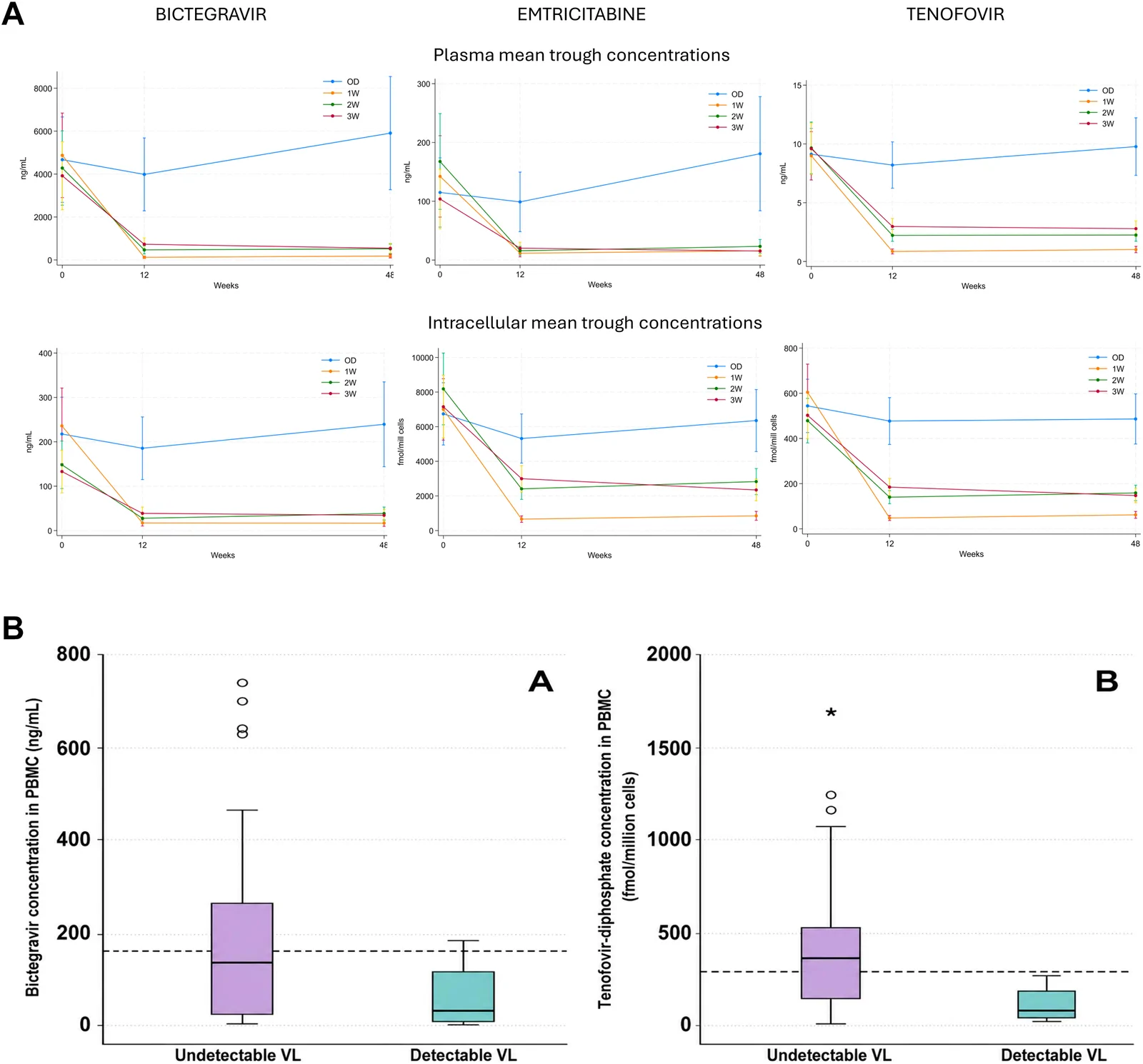
(A) Plasma and intracellular drug trough concentrations at baseline, week 12, and week 48. Model-predicted mean trough concentrations of bictegravir, emtricitabine, and tenofovir in plasma, and intracellular bictegravir, emtricitabine-triphosphate, and tenofovir-diphosphate concentrations in peripheral-blood mononuclear cells, are shown by treatment group at baseline, week 12, and week 48. Error bars indicate SDs. BIC = bictegravir; FTC-TP = emtricitabine-triphosphate; TFV-DP = tenofovir-diphosphate; PBMCs = peripheral-blood mononuclear cells; OD = once daily; 3 W = three times weekly; 2 W = twice weekly; 1 W = once weekly; SD = standard deviation. (B) Intracellular bictegravir and tenofovir-diphosphate concentrations in PBMCs according to HIV-1 RNA status. (A) Intracellular bictegravir concentrations in PBMCs. (B) Intracellular tenofovir-diphosphate concentrations in PBMCs. Detectable viraemia was defined as HIV-1 RNA ≥50 copies per mL. Light lavender boxes indicate samples with HIV-1 RNA < 50 copies per mL, and light teal boxes indicate samples with HIV-1 RNA ≥50 copies per mL. Boxes represent median and interquartile range, whiskers indicate adjacent values, and dots represent outlier values. Dashed horizontal lines indicate exploratory pharmacological reference values: 162 ng/mL for bictegravir and 290 fmol/10^6^ cells for tenofovir-diphosphate. The intracellular application of the bictegravir plasma IC95 reference and the tenofovir-diphosphate EC90-derived reference are descriptive and should not be interpreted as validated clinical cutoffs or treatment targets. BIC = bictegravir; PBMCs = peripheral-blood mononuclear cells; TFV-DP = tenofovir-diphosphate; VL = viral load.

Trough plasma and intracellular antiretroviral concentrations were lower in reduced-frequency arms than in the OD arm at weeks 12 and 48 (Fig. 4A). In the OD arm, some analytes were numerically higher at week 48 than at week 12, but variability estimates overlapped, supporting interpretation as small-sample and interindividual variability rather than a biologically meaningful exposure increase. Concentrations in the 3 W and 2 W arms remained generally above the exploratory pharmacological reference values, whereas concentrations in the 1 W arm were persistently lower, consistent with the early protocol-defined confirmed virological failure events in that group. These two 1 W events occurred at week 4, before the first scheduled post-baseline pharmacokinetic assessment, so drug concentrations at the time of failure were unavailable.

Concomitant medications are summarised in Supplementary Table S4. No concomitant medication judged likely to reduce B/F/TAF exposure was identified; specifically, no participant received strong enzyme inducers, St John's wort, sucralfate, or polyvalent cation-containing antacids or supplements.

Detectable viraemia occurred more often at timepoints with lower intracellular bictegravir and TFV-DP concentrations (Fig. 4B). Exploratory ROC analyses of plasma and intracellular drug concentrations as continuous predictors of detectable viraemia are shown in Supplementary Fig. S2, with AUROC estimates in Supplementary Table S5. Intracellular TFV-DP showed the highest discriminatory ability (AUROC 0.791, p < 0.001), followed by intracellular bictegravir (AUROC 0.739, p = 0.016), plasma bictegravir (AUROC 0.685, p = 0.082), and intracellular FTC-TP (AUROC 0.651, p = 0.196). These analyses were exploratory and pooled concentration–viraemia pairs across follow-up timepoints; derived values should not be interpreted as validated clinical cutoffs.

Adherence by SMAQ was high throughout follow-up. Most participants reported no missed doses in the preceding week, patterns were similar across dosing groups, and questionnaire completion ranged from 97% to 100% across visits (Supplementary Table S6).

Eighteen participants experienced 24 adverse events. All were mild, none were considered related to study treatment, and no serious adverse events, adverse-event-related treatment discontinuations, or laboratory abnormalities requiring treatment modification occurred. In post hoc laboratory-abnormality analyses, grade 2 or higher ALT or AST elevations (≥2.5 × upper limit of normal) occurred in 0/9, 0/10, 0/10, and 0/10 participants in the OD, 3 W, 2 W, and 1 W arms, respectively. A decline of at least 25% in estimated glomerular filtration rate from baseline occurred in 0/9, 0/10, 0/10, and 0/10 participants, respectively. Among participants with resolved HBV infection, ALT and AST remained stable, no transaminase elevations above the upper limit of normal occurred, and no adverse event was reported as hepatitis or suspected HBV reactivation. Follow-up HBV DNA and repeat HBsAg testing were not protocol-mandated, so subclinical HBV reactivation or HBsAg seroreversion cannot be fully excluded. Adverse-event incidence rates per 100 person-years were 46.6 (95% CI 0.9–92.2) for OD, 62.5 (12.5–112.5) for 1 W, 76.9 (23.6–130.2) for 2 W, and 57.7 (11.5–103.8) for 3 W (p = 0.864). The most frequent system-organ classes were musculoskeletal and infections (eight events each) (Supplementary Table S7). Infection-related events included lymphogranuloma venereum proctitis, syphilis, *Neisseria gonorrhoeae* infection, and incident HCV infection. No suspected linked HIV transmission event or new partner HIV diagnosis was documented, although partner information and testing were not systematically collected.

Body weight, body mass index, and DXA-derived total body fat percentage remained stable, with no clinically meaningful changes within groups or differences between dosing strategies (Supplementary Table S8). Biochemical parameters were clinically stable, including renal function, liver enzymes, glucose, electrolytes, and lipids (Supplementary Table S9). Serum creatinine decreased modestly in the 2 W arm from baseline to week 48, but this change was not accompanied by any ≥25% decline in estimated glomerular filtration rate, renal adverse event, treatment discontinuation, or laboratory abnormality requiring treatment modification. Alkaline phosphatase decreased in the 2 W arm without associated liver changes or clinical consequences. LDL cholesterol increased modestly in the 1 W and 2 W arms, while other lipid parameters remained stable. These changes were small, inconsistent, and not clinically meaningful.

Markers of systemic inflammation showed no consistent changes from baseline to week 48 and no differences between arms (Supplementary Table S10). Self-reported sleep quality improved modestly across all dosing strategies, with no significant between-arm differences in trajectories (Supplementary Table S11). Health-related quality of life remained high and stable, with no meaningful changes across EQ-5D-5L domains or visual analogue scale scores (Supplementary Table S12).

## Discussion

In this small, single-centre, randomised phase 2 pilot trial, reduced-frequency B/F/TAF generated descriptive virological and pharmacokinetic signals, but the findings do not support clinical implementation and do not permit formal comparisons between dosing schedules. Early protocol-defined confirmed virological failures were observed in the once-weekly arm, whereas twice-weekly and three-times-weekly dosing require confirmation in larger, adequately powered trials under close virological monitoring. Because participants were already virologically suppressed on once-daily B/F/TAF, the primary endpoint should be interpreted as maintenance of suppression after a reduced-frequency switch strategy, not as treatment-induction efficacy.

The protocol-defined confirmed virological failure criterion was intentionally conservative. Because the intervention deliberately reduced antiretroviral exposure in suppressed participants, two consecutive HIV-1 RNA values ≥50 copies per mL were used as an early safety trigger for return to once-daily B/F/TAF. This definition should not be interpreted as equivalent to clinical treatment failure in routine care, where higher thresholds, commonly around 200 copies per mL, are used in some guideline frameworks.^22^ This conservative criterion may therefore have increased the number of participants classified as protocol-defined failures compared with routine-care definitions.

The findings are consistent with the pharmacological properties of bictegravir, emtricitabine, and tenofovir alafenamide, including prolonged intracellular persistence and a high genetic barrier to resistance. The results are compatible with maintained suppression over 48–72-h dosing intervals in selected participants under close monitoring, but not with a single weekly exposure. These findings support the biological plausibility of reduced-frequency oral triple therapy; however, they also underscore that such strategies remain investigational.^9^, ^10^, ^11^^,^^23^

The pharmacokinetic findings provide a plausible explanation for the observed virological pattern. However, intracellular concentration thresholds that predict maintained virological suppression under reduced-frequency dosing remain uncertain. Bictegravir trough concentrations under three-times-weekly and twice-weekly dosing generally remained above the protein-adjusted IC95 used as a plasma reference value,^9^^,^^24^ whereas concentrations were lower with once-weekly dosing. For intracellular TFV-DP, published pharmacodynamic data provide exploratory reference values, including lower EC50 estimates of approximately 75–125 fmol/10^6^ cells and an approximate EC90 value of 290 fmol/10^6^ cells.^21^^,^^25^^,^^26^ These are extrapolated pharmacological references, not validated clinical targets for maintenance treatment. We used the higher value as a conservative exploratory reference for intracellular TFV-DP exposure, but the threshold required to maintain suppression in virologically suppressed people receiving reduced-frequency B/F/TAF remains unknown. Published data also support the biological relevance of intracellular TFV-DP exposure to virological and reservoir-related markers,^19^ while FTC-TP concentrations were interpreted descriptively in the context of previous pharmacokinetic–pharmacodynamic data.^27^

Exploratory ROC analyses supported a concentration–viraemia relationship, with intracellular TFV-DP and intracellular bictegravir showing the strongest discrimination for detectable viraemia. However, these analyses included few detectable viraemia events and pooled concentration–outcome pairs across follow-up timepoints. In addition, the two early protocol-defined confirmed virological failure events in the once-weekly arm occurred before scheduled week-12 pharmacokinetic sampling, limiting direct assessment of exposure at failure. The pharmacokinetic thresholds shown in this study should therefore be interpreted as exploratory pharmacological references, not validated clinical cutoffs. Overall, the pharmacokinetic and virological findings suggest that twice-weekly and three-times-weekly B/F/TAF may warrant further evaluation under close monitoring. The once-weekly arm showed early protocol-defined confirmed virological failures in the context of lower measured exposure, but the trial was too small for formal between-arm comparisons.

Reservoir analyses provided additional context. Ultrasensitive plasma RNA, intact and defective HIV DNA, and intracellular HIV RNA species showed no evidence of reservoir expansion or increased residual transcription with reduced-frequency schedules. The two early protocol-defined confirmed virological failure events in the once-weekly arm occurred in participants with among the highest baseline intact proviral DNA and LongLTR icRNA levels within that arm, suggesting that higher reservoir burden or transcriptional activity might increase rebound risk under suboptimal exposure. This observation is consistent with prior evidence linking reservoir size to virological failure during dolutegravir monotherapy,^28^ but remains exploratory. Future trials should include systematic reservoir profiling and prespecified analyses by baseline reservoir burden.^29^^,^^30^ CD4^+^ and CD8^+^ counts, inflammatory markers, metabolic indices, and patient-reported outcomes showed no clinically relevant changes, supporting short-term tolerability in this selected population.

Pharmacologically guided oral simplification could, in principle, complement long-acting injectable strategies by offering another approach to reducing dosing burden. However, the present findings should be interpreted as biological and pharmacokinetic proof-of-concept, not as evidence for clinical implementation. The development of once-weekly oral regimens specifically designed for extended dosing intervals supports interest in non-daily oral strategies, but these approaches require regimen-specific evidence and cannot be extrapolated from this pilot study.^31^

This study has limitations. The small sample size, single-centre design, and 48-week follow-up limited the ability to compare efficacy between dosing strategies, detect late virological failure or rare resistance events, and generalise the findings. The cohort was predominantly male, relatively young, highly adherent, had CD4 counts above 350 cells/μL, had sustained suppression before enrolment, and was selected for close trial monitoring; findings should therefore not be extrapolated to pregnant individuals, older adults, people with lower CD4 counts, or individuals with unstable adherence. Sex-specific analyses were not feasible because only three female participants were enrolled. Gender identity, race, and ethnicity were not collected, limiting assessment of representativeness and generalisability across sociocultural populations and structural determinants of health. HBV safety also requires caution. Participants with HBsAg-positive chronic HBV infection were excluded. Among those with resolved HBV infection, clinically significant reactivation was expected to be unlikely because anti-HBs was present and TAF/FTC exposure was reduced rather than withdrawn, although reactivation has been described in higher-risk settings, particularly isolated anti-HBc and fully tenofovir-sparing regimens.^32^ No clinical or biochemical signal suggestive of HBV reactivation was observed, but follow-up HBV DNA and repeat HBsAg testing were not protocol-mandated; subclinical reactivation or HBsAg seroreversion cannot be excluded. These findings should not be extrapolated to chronic HBV infection or to anti-HBc-positive individuals without appropriate monitoring. Finally, this pilot trial was not designed to assess HIV transmission risk. No suspected linked HIV transmission event or new partner HIV diagnosis was documented, but partner information and testing were not systematically collected. Because confirmed viraemia during experimental reduced-frequency strategies may have implications beyond the individual participant, future studies should include close viral-load monitoring, systematic screening for sexually transmitted infections, counselling during confirmed viraemia, and prospective collection of partner-related outcomes where appropriate; partner-protection guidance from analytical treatment interruption studies provides a useful framework, although analytical treatment interruption differs from reduced-frequency antiretroviral therapy.^33^

In summary, BETAF-RED provides preliminary virological, pharmacokinetic, and reservoir data on reduced-frequency B/F/TAF in selected, virologically suppressed adults under close monitoring. Early protocol-defined confirmed virological failures were observed in the once-weekly arm, and one confirmed low-level viraemia event occurred in the three-times-weekly arm. Twice-weekly and three-times-weekly dosing showed exploratory signals of maintained suppression in most participants, but this pilot trial was not powered for definitive efficacy conclusions, formal between-arm comparisons, or regimen selection for implementation. Larger, adequately powered multicentre studies are required before reduced-frequency B/F/TAF can be considered for clinical use.

## Contributors

EM conceived and designed the study. IC, AS, PC, ADN, BT, JB, JLB, ADA, and EM contributed to data collection. EMo, SVA, CR, CH, ADN, ASM, JA, ADA, and SSP contributed to laboratory analyses. EL performed the statistical analysis. IC, EMo, EL, and EM wrote the original draft of the manuscript. IC, EMo, AS, SVA, PC, CR, CH, ADN, ASM, BT, JB, JA, JLB, ADA, SSP, EL, and EM contributed to manuscript review and editing. IC, EM, ADN, and EL accessed and verified the underlying data. EL and EM had full access to all the data in the study. EM was responsible for the decision to submit the manuscript. All authors read and approved the final version for submission. EM is the corresponding author and guarantor of the study.

## Data sharing statement

De-identified individual participant data will not be made publicly available because of the small sample size, participant confidentiality, and the terms of informed consent and ethics approval. Requests for access to de-identified data may be considered by the corresponding author after publication, subject to appropriate ethics and institutional approvals and a data-sharing agreement.

## Declaration of interests

BT reports grants from GSK/ViiV Healthcare paid to her institution; honoraria for lectures or educational activities from GSK/ViiV Healthcare, Johnson & Johnson, Gilead Sciences, and MSD; payment for expert testimony from GSK/ViiV Healthcare and Johnson & Johnson; support for attending meetings from GSK/ViiV Healthcare; and advisory board participation for GSK/ViiV Healthcare.

JB reports grants from ViiV Healthcare, Gilead Sciences, and MSD to support a Neuropsychiatry and HIV meeting; honoraria for a conference presentation from the University of Liverpool; and support for attending meetings from Gilead Sciences, MSD, and ViiV Healthcare.

JA reports honoraria for educational activities from Gilead Sciences and ViiV Healthcare.

EM reports grants from Gilead Sciences and ViiV Healthcare paid to his institution, personal fees from MSD and ViiV Healthcare for advisory board participation, and unpaid leadership roles in EACS and NEAT-ID.

IC, EMo, AS, SVA, PC, CR, CH, ADN, ASM, JLB, ADA, SSP, and EL declare no competing interests.
