# Supplementary material for: Reduced-frequency bictegravir, emtricitabine, and tenofovir alafenamide in virologically suppressed adults with HIV: a single-centre randomised phase 2 pilot trial in Spain
Source: eClinicalMedicine. 2026 Aug 23;99:104158. doi: 10.1016/j.eclinm.2026.104158 (PMC13524659; doi:10.1016/j.eclinm.2026.104158)
Supplement: Supplementary file 1 [file mmc1.pdf]

Supplementary appendix

Reduced-frequency bictegravir, emtricitabine, and tenofovir alafenamide in virologically suppressed adults with HIV: a single-centre randomised phase 2 pilot trial in Spain

BETAF-RED trial

Index

Supplementary material captions S3

Supplementary tables S7

Supplementary Table 1. Participant-level details of protocol-defined confirmed virological failure S7
Supplementary Table 2. Viral blips during follow-up S9
Supplementary Table 3. Changes in CD4+ T-cell count, CD8+ T-cell count, and CD4+:CD8+ ratio from baseline to week 48 S10
Supplementary Table 4. Concomitant medication entries recorded during follow-up, by treatment arm S12
Supplementary Table 5. Area under the ROC curve for pharmacokinetic predictors of detectable viraemia S13
Supplementary Table 6. Self-reported adherence (SMAQ questionnaire) S14
Supplementary Table 7. Distribution of adverse events by system-organ class and treatment group S16
Supplementary Table 8. Body weight, BMI, BMI categories, and total body fat percentage by DXA S17
Supplementary Table 9. Changes in biochemical parameters from baseline to week 48 S19
Supplementary Table 10. Detectability of inflammatory markers (IL-6 and hsCRP) S22
Supplementary Table 11. Proportion of participants with good sleep quality (PSQI <=5) S23
Supplementary Table 12. Health-related quality of life assessed by EQ-5D-5L and EQ-VAS S24

Supplementary figures S25

Supplementary Figure 1A. Ultrasensitive plasma HIV-1 RNA levels at baseline and week 48 S25
Supplementary Figure 1B. Distribution of ultrasensitive plasma HIV-1 RNA categories at baseline and week 48 S26
Supplementary Figure 2. Receiver operating characteristic curves for pharmacokinetic predictors of detectable viraemia S27

Study protocol. BETAF-RED protocol, version 1.1, 10 April 2022 S28

The original page size and orientation of each supplied document have been preserved.

Supplementary materials

Supplementary Table 1. Participant-level details of protocol-defined confirmed virological failure

Data are shown for participants with protocol-defined confirmed virological failure. All confirmatory samples were obtained within the protocol-specified 2-week confirmatory window.
B/F/TAF=bictegravir, emtricitabine, and tenofovir alafenamide; ITT-exposed=intention-to-treat exposed; OD=once daily; 1W=once weekly; 3W=three times weekly; RT=reverse transcriptase; PR=protease.

Supplementary Table 2. Viral blips during follow-up

Data are n (%). A viral blip was defined as a single HIV-1 RNA value ≥50 copies per mL followed by resuppression to <50 copies per mL without treatment modification. Protocol-defined confirmed virological failure events were not counted as viral blips.
OD=once daily; 3W=three times weekly; 2W=twice weekly; 1W=once weekly.

Supplementary Table 3. Changes in $CD4^+$ T-cell count, $CD8^+$ T-cell count, and $CD4^+$:$CD8^+$ ratio from baseline to week 48, by treatment group

P values of each model:

- $CD4^+$ T-cell count: p=0.748
- $CD8^+$ T-cell count: p=0.550
- $CD4^+$:$CD8^+$ ratio: p=0.156

Data are mean change from baseline to week 48 with 95% CI, estimated using linear mixed-effects models. Between-group differences were assessed using group-by-time interaction terms.
OD=once daily; 3W=three times weekly; 2W=twice weekly; 1W=once weekly.

Supplementary Table 4. Concomitant medication entries recorded during follow-up, by treatment arm

Data are the number of concomitant medication entries recorded during follow-up. Participants could contribute more than one medication entry. No participant received rifampicin, rifabutin, rifapentine, carbamazepine, oxcarbazepine, phenobarbital, phenytoin, St John’s wort, sucralfate, cyclosporine, or magnesium-, aluminium-, calcium-, or iron-containing products during the study. Vitamin D preparations were not considered equivalent to polyvalent cation-containing mineral supplementation for the purpose of bictegravir chelation.
OD=once daily; 3W=three times weekly; 2W=twice weekly; 1W=once weekly; B/F/TAF=bictegravir, emtricitabine, and tenofovir alafenamide.

Supplementary Table 5. Area under the ROC curve for pharmacokinetic predictors of detectable viraemia

Data derived from receiver operating characteristic (ROC) curve analyses evaluating the ability of plasma and intracellular drug concentrations to discriminate detectable versus undetectable plasma HIV-1 RNA.
AUC=area under the curve.

Supplementary Table 6. Self-reported adherence (SMAQ questionnaire): number of missed doses in the previous week, by treatment group

Data are n (%), with column percentages. Adherence was assessed using the SMAQ questionnaire.
OD=once daily; 3W=three times weekly; 2W=twice weekly; 1W=once weekly; NA=not applicable.

Supplementary Table 7. Distribution of adverse events by system-organ class and treatment group

Data are number of events (% within treatment group). Percentages are calculated using the total number of adverse events in each treatment group as denominator. Among infection-related adverse events, four sexually transmitted or sexually associated events were reported: lymphogranuloma venereum proctitis (n=1), syphilis (n=1), *Neisseria gonorrhoeae* infection (n=1), and incident HCV infection documented by HCV RNA positivity (n=1).
OD=once daily; 3W=three times weekly; 2W=twice weekly; 1W=once weekly.

Supplementary Table 8. Body weight, BMI, BMI categories, and total body fat percentage by DXA at baseline and week 48, by treatment group

Data are mean (SD) [n] or n (%). Changes from baseline to week 48 were estimated using linear mixed-effects models. Between-group differences were assessed using group-by-time interaction terms.
OD=once daily; 3W=three times weekly; 2W=twice weekly; 1W=once weekly.

Supplementary Table 9. Changes in biochemical parameters from baseline to week 48 according to treatment group

Data are estimated using mixed-effects models. Results are presented as mean change from baseline to week 48 with 95% confidence intervals, except for CKD-EPI, which is presented as a ratio estimate with 95% confidence intervals.
OD=once daily; 1W=once weekly; 2W=twice weekly; 3W=three times weekly.

Supplementary Table 10. Detectability of inflammatory markers (IL-6 and hsCRP) at baseline and week 48, by treatment group

Data are n (%). Percentages are calculated within treatment group. Detectability was defined as a value at or above the lower limit of quantification: 2 pg/mL for IL-6 and 0.02 mg/dL for hsCRP.
OD=once daily; 3W=three times weekly; 2W=twice weekly; 1W=once weekly.

Supplementary Table 11. Proportion of participants with good sleep quality (PSQI ≤5), by treatment group and visit

Data are n (% within treatment group). Poor sleep quality corresponds to PSQI >5.
OD=once daily; 1W=once weekly; 2W=twice weekly; 3W=three times weekly; NA=not applicable.

Supplementary Table 12. Health-related quality of life assessed by EQ-5D-5L and EQ-VAS, by treatment group and visit

Data are n (% within treatment group) for EQ-5D-5L domains and mean (SD) [n] for EQ-VAS. Higher EQ-VAS scores indicate better perceived health status.
OD=once daily; 1W=once weekly; 2W=twice weekly; 3W=three times weekly.

Supplementary Figure 1A. Ultrasensitive plasma HIV-1 RNA levels at baseline and week 48

Ultrasensitive plasma HIV-1 RNA levels are shown in copies per mL at baseline and week 48 by treatment arm. Boxes represent median and interquartile range, whiskers indicate adjacent values, and dots represent outlier values. The dashed horizontal line indicates the assay limit of detection of 5 copies per mL.
OD=once daily; 3W=three times weekly; 2W=twice weekly; 1W=once weekly.

Supplementary Figure 1B. Distribution of ultrasensitive plasma HIV-1 RNA categories at baseline and week 48

Distribution of ultrasensitive plasma HIV-1 RNA categories at baseline and week 48 by treatment arm. Categories were defined as <5 copies/mL, no signal; <5 copies/mL, signal detected; and ≥5 copies/mL.
OD=once daily; 3W=three times weekly; 2W=twice weekly; 1W=once weekly.

Supplementary Figure 2. Receiver operating characteristic curves for pharmacokinetic predictors of detectable viraemia

Receiver operating characteristic curves were used to assess plasma bictegravir and intracellular bictegravir, emtricitabine-triphosphate, and tenofovir-diphosphate concentrations as continuous predictors of detectable viraemia, defined as HIV-1 RNA ≥50 copies per mL. The analysis used matched pharmacokinetic concentration–HIV-1 RNA pairs from available study timepoints, pooled across participants and visits. Plasma BIC AUROC was 0·685 (p=0·082), intracellular BIC AUROC was 0·739 (p=0·016), intracellular TFV-DP AUROC was 0·791 (p<0·001), and intracellular FTC-TP AUROC was 0·651 (p=0·196). Because few detectable viraemia events occurred and repeated observations from the same participants were pooled, these analyses are exploratory and should not be interpreted as defining validated clinical cutoffs or treatment targets.

BIC=bictegravir; FTC-TP=emtricitabine triphosphate; TFV-DP=tenofovir diphosphate; AUROC=area under the receiver operating characteristic curve.

Supplementary Table 1. Participant-level details of protocol-defined confirmed virological failure

Participant ID	Assigned arm	Event week	First HIV-1 RNA date	First HIV-1 RNA, copies per mL	Confirmatory HIV-1 RNA date	Confirmatory HIV-1 RNA, copies per mL	Interval between samples	Date of restart of once-daily B/F/TAF	Subsequent virological outcome	Genotypic resistance testing	ITT-exposed FDA Snapshot category, week 12	ITT-exposed FDA Snapshot category, week 48
22	3W	Week 12	2023-07-28	55	2023-08-09	60	12 days	2023-08-11	HIV-1 RNA <50 copies per mL from week 24 through week 48	Not performed because HIV-1 RNA was too low for reliable genotypic testing	FDA Snapshot virological failure category	FDA Snapshot virological failure category
30	1W	Week 4	2023-07-05	617	2023-07-19	29 800	14 days	2023-07-21	HIV-1 RNA <50 copies per mL from week 12 through week 48	Performed; RT T215A and PR L58E detected	FDA Snapshot virological failure category	FDA Snapshot virological failure category
39	1W	Week 4	2023-07-26	180	2023-08-02	780	7 days	2023-08-07	HIV-1 RNA <50 copies per mL from week 12 through week 48	Performed; RT K103R and RT M41L detected	FDA Snapshot virological failure category	FDA Snapshot virological failure category

Data are shown for participants with protocol-defined confirmed virological failure. All confirmatory samples were obtained within the protocol-specified 2-week confirmatory window.

B/F/TAF=bictegravir, emtricitabine, and tenofovir alafenamide; ITT-exposed=intention-to-treat exposed; OD=once daily; 1W=once weekly; 3W=three times weekly; RT=reverse transcriptase; PR=protease.

Supplementary Table 2. Viral blips during follow-up

Timepoint	Outcome	OD (n=9)	3W (n=10)	2W (n=10)	1W (n=10)	Total
Week 12	No blip	9 (100%)	9 (90%)	9 (90%)	9 (90%)	36 (95%)
	Blip	0 (0%)	1 (10%)	1 (10%)	1 (10%)	3 (5%)
Week 48	No blip	8 (100%)	10 (100%)	10 (100%)	7 (88%)	35 (97%)
	Blip	0 (0%)	0 (0%)	0 (0%)	1 (12%)	1 (3%)

Data are n (%).
A viral blip was defined as a single HIV-1 RNA value ≥50 copies per mL followed by resuppression to <50 copies per mL without treatment modification. Protocol-defined confirmed virological failure events were not counted as viral blips.
OD = once daily; 3W = three times weekly; 2W = twice weekly; 1W = once weekly.

Supplementary Table 3. Changes in $CD4^+$ T-cell count, $CD8^+$ T-cell count, and $CD4^+$:$CD8^+$ ratio from baseline to week 48, by treatment group

Outcome	Treatment group	Mean change from baseline to week 48 (95% CI)	p value
$CD4^+$ T-cell count (cells/μL)	OD	−23.55 (−154.70 to 107.60)	0.725
	1W	+134.39 (−21.44 to 290.22)	0.091
	2W	−114.97 (−237.05 to 7.11)	0.065
	3W	+32.23 (−89.85 to 154.30)	0.605
$CD8^+$ T-cell count (cells/μL)	OD	−83.68 (−236.45 to 69.09)	0.283
	1W	+141.76 (−39.74 to 323.25)	0.126
	2W	−111.53 (−253.73 to 30.67)	0.124
	3W	−36.81 (−179.01 to 105.39)	0.612
$CD4^+$:$CD8^+$ ratio	OD	+0.20 (0.05 to 0.34)	0.008
	1W	+0.11 (−0.07 to 0.28)	0.233
	2W	+0.02 (−0.11 to 0.15)	0.764
	3W	+0.14 (0.01 to 0.28)	0.035

P values of each model:

- $CD4^+$ T-cell count: p=0.748
- $CD8^+$ T-cell count: p=0.550
- $CD4^+$:$CD8^+$ ratio: p=0.156

Data are mean change from baseline to week 48 with 95% CI, estimated using linear mixed-effects models. Between-group differences were assessed using group-by-time interaction terms.

OD=once daily; 3W=three times weekly; 2W=twice weekly; 1W=once weekly.

Supplementary Table 4. Concomitant medication entries recorded during follow-up, by treatment arm

Concomitant medication category	OD	3W	2W	1W	Total
Systemic short-course antibiotics	2	0	4	1	7
Analgesics or anti-inflammatory drugs	0	4	2	1	7
Topical, ophthalmic, or nasal treatments	0	4	2	0	6
Vitamin D preparations	1	2	3	1	7
Lipid-lowering therapy	1	0	2	0	3
Antihypertensive drugs	0	1	1	0	2
Proton-pump inhibitors	0	0	1	0	1
Sleep/anxiolytic treatments	0	0	2	0	2
Antihistamines	0	1	0	0	1
Hormonal therapy	0	1	0	0	1
**Total concomitant medication entries**	**4**	**13**	**17**	**3**	**37**
**Concomitant medications judged likely to substantially reduce B/F/TAF exposure**	**0**	**0**	**0**	**0**	**0**

Data are the number of concomitant medication entries recorded during follow-up. Participants could contribute more than one medication entry. No participant received rifampicin, rifabutin, rifapentine, carbamazepine, oxcarbazepine, phenobarbital, phenytoin, St John's wort, sucralfate, cyclosporine, or magnesium-, aluminium-, calcium-, or iron-containing products during the study. Vitamin D preparations were not considered equivalent to polyvalent cation-containing mineral supplementation for the purpose of bictegravir chelation.

OD=once daily; 3W=three times weekly; 2W=twice weekly; 1W=once weekly; B/F/TAF=bictegravir, emtricitabine, and tenofovir alafenamide.

Supplementary Table 5. Area under the ROC curve for pharmacokinetic predictors of detectable viraemia

Variable	AUC	Standard Error	p value	95% CI Lower	95% CI Upper
**Plasma bictegravir**	0.685	0.106	0.082	0.476	0.893
**Intracellular bictegravir**	0.739	0.099	0.016	0.544	0.933
**Intracellular emtricitabine-triphosphate**	0.651	0.117	0.196	0.422	0.880
**Intracellular tenofovir-diphosphate**	0.791	0.073	<0.001	0.648	0.933

Data derived from receiver operating characteristic (ROC) curve analyses evaluating the ability of plasma and intracellular drug concentrations to discriminate detectable versus undetectable plasma HIV-1 RNA.
AUC = area under the curve.

Supplementary Table 6. Self-reported adherence (SMAQ questionnaire): number of missed doses in the previous week, by treatment group

Timepoint	Missed doses in previous week	OD (n=9)	3W (n=10)	2W (n=10)	1W (n=10)	Total
Week 4	0 missed doses	NA	9 (90%)	10 (100%)	10 (100%)	29 (100%)
	1 missed dose	NA	1 (10%)	0 (0%)	0 (0%)	1 (3%)
	≥2 missed doses	NA	0 (0%)	0 (0%)	0 (0%)	0 (0%)
Week 12	0 missed doses	6 (67%)	10 (100%)	10 (100%)	8 (89%)	34 (89%)
	1 missed dose	1 (11%)	0 (0%)	0 (0%)	1 (11%)	2 (5%)
	≥2 missed doses	2 (22%)	0 (0%)	0 (0%)	0 (0%)	2 (6%)
Week 24	0 missed doses	7 (88%)	8 (80%)	7 (88%)	8 (89%)	32 (89%)
	1 missed dose	1 (12%)	2 (20%)	1 (12%)	1 (11%)	5 (11%)
	≥2 missed doses	0 (0%)	0 (0%)	0 (0%)	0 (0%)	0 (0%)
Week 36	0 missed doses	6 (75%)	8 (80%)	8 (89%)	9 (100%)	31 (86%)
	1 missed dose	2 (25%)	2 (20%)	0 (0%)	0 (0%)	4 (11%)
	≥2 missed doses	0 (0%)	0 (0%)	0 (0%)	0 (0%)	0 (0%)
Week 48	0 missed doses	5 (62%)	9 (100%)	10 (100%)	8 (100%)	32 (91%)
	1 missed dose	3 (38%)	0 (0%)	0 (0%)	0 (0%)	3 (9%)

Timepoint	Missed doses in previous week	OD (n=9)	3W (n=10)	2W (n=10)	1W (n=10)	Total
	≥2 missed doses	0 (0%)	0 (0%)	0 (0%)	0 (0%)	0 (0%)

Data are n (%), with column percentages. Adherence was assessed using the SMAQ questionnaire.
OD = once daily; 3W = three times weekly; 2W = twice weekly; 1W = once weekly. NA= not applicable.

Supplementary Table 7. Distribution of adverse events by system-organ class and treatment group

System-organ class	OD (n=4 events)	1W (n=6 events)	2W (n=8 events)	3W (n=6 events)	Total (n=24 events)
Musculoskeletal	1 (25%)	0 (0%)	5 (62%)	2 (33%)	8 (33%)
Infections	2 (50%)	2 (33%)	1 (12%)	3 (50%)	8 (33%)
Neurological	0 (0%)	2 (33%)	1 (12%)	0 (0%)	3 (12%)
Gastrointestinal	1 (25%)	1 (17%)	0 (0%)	0 (0%)	2 (8%)
Cardiovascular	0 (0%)	0 (0%)	0 (0%)	1 (17%)	1 (4%)
Dermatologic	0 (0%)	1 (17%)	0 (0%)	0 (0%)	1 (4%)
Ocular/visual	0 (0%)	0 (0%)	1 (12%)	0 (0%)	1 (4%)
**Total**	**4 (100%)**	**6 (100%)**	**8 (100%)**	**6 (100%)**	**24 (100%)**

Data are number of events (% within treatment group). Percentages are calculated using the total number of adverse events in each treatment group as denominator. Among infection-related adverse events, four sexually transmitted or sexually associated events were reported: lymphogranuloma venereum proctitis (n=1), syphilis (n=1), *Neisseria gonorrhoeae* infection (n=1), and incident HCV infection documented by HCV RNA positivity (n=1).

OD=once daily; 3W=three times weekly; 2W=twice weekly; 1W=once weekly.

Supplementary Table 8. Body weight, BMI, BMI categories, and total body fat percentage by DXA at baseline and week 48, by treatment group

Outcome	Timepoint	OD (n=9)	3W (n=10)	2W (n=10)	1W (n=10)	Total
Body weight (kg), mean (SD)	Baseline	75 (11)	77 (10)	79 (18)	75 (9)	76 (12)
	Week 48	75 (12)	81 (12)	76 (8)	75 (12)	76 (11)
	Mean change (95% CI)	+0.21 (–2.28 to 2.70)	+1.18 (–1.84 to 4.21)	+1.73 (–0.59 to 4.05)	–0.11 (–2.42 to 2.21)	—
	p value of the model					0.826
BMI (kg/m²), mean (SD)	Baseline	24.5 (3.8)	24.7 (3.9)	26.1 (5.4)	24.4 (2.5)	25.0 (4.0)
	Week 48	24.6 (4.0)	28.2 (5.6)	25.5 (2.8)	24.2 (3.3)	25.3 (3.8)
	Mean change (95% CI)	+0.05 (–0.97 to 1.07)	+1.02 (–0.27 to 2.31)	+0.95 (0.00 to 1.89)	–0.27 (–1.28 to 0.74)	—
	p value of the model					0.593
BMI categories, n (%)	Baseline Normal weight	8 (100%)	7 (78%)	8 (89%)	8 (100%)	31 (91%)
	Baseline Obese	0 (0%)	2 (22%)	1 (11%)	0 (0%)	3 (9%)
	Week 48 Normal weight	6 (86%)	2 (50%)	8 (100%)	7 (100%)	23 (88%)
	Week 48 Obese	1 (14%)	2 (50%)	0 (0%)	0 (0%)	3 (12%)
Total body fat (%) by DEXA, mean (SD)	Baseline	24 (7)	28 (6)	33 (10)	23 (6)	27 (9)
	Week 48	22 (7)	29 (11)	34 (8)	24 (7)	27 (9)
	Mean change (95% CI)	–0.44 (–2.43 to 1.55)	–0.20 (–2.32 to 1.92)	+1.34 (–0.64 to 3.33)	+0.86 (–1.13 to 2.84)	—
	p value of the model					0.055

Data are mean (SD) [n] or n (%). Changes from baseline to week 48 were estimated using linear mixed-effects models. Between-group differences were assessed using group-by-time interaction terms.

OD=once daily; 3W=three times weekly; 2W=twice weekly; 1W=once weekly.

Supplementary Table 9. Changes in biochemical parameters from baseline to week 48 according to treatment group

	Treatment group	Mean change from baseline to week 48 (95% CI)	p value
**Glucose (mg/dL)**	OD	+7.36 (−2.77 to 17.48)	0.154
	1W	−5.10 (−17.01 to 6.81)	0.401
	2W	−0.32 (−9.75 to 9.10)	0.947
	3W	−1.26 (−10.69 to 8.16)	0.793
**Creatinine (mg/dL)**	OD	+0.04 (−0.02 to 0.09)	0.184
	1W	−0.03 (−0.09 to 0.04)	0.400
	2W	−0.06 (−0.11 to −0.00)	0.032
	3W	−0.03 (−0.08 to 0.02)	0.208
**CKD-EPI ≥90 (mL/min/1.73 m²)**	OD	+1.25 (0.80 to 1.95)	0.525
	1W	+1.46 (0.88 to 2.38)	0.131
	2W	+1.33 (0.89 to 2.00)	0.164
	3W	+1.29 (0.90 to 1.83)	0.164
**Total cholesterol (mg/dL)**	OD	+10.73 (−7.02 to 28.53)	0.237
	1W	+3.21 (−18.05 to 24.47)	0.767
	2W	+6.24 (−10.33 to 22.81)	0.461
	3W	+11.61 (−4.95 to 28.18)	0.169
**LDL cholesterol (mg/dL)**	OD	+9.72 (−1.00 to 20.44)	0.076
	1W	+19.72 (0.07 to 39.37)	0.049
	2W	+8.93 (−1.62 to 19.48)	0.097
	3W	+19.34 (6.27 to 32.41)	0.004
**HDL cholesterol (mg/dL)**	OD	−3.28 (−8.98 to 2.44)	0.264
	1W	−0.70 (−7.55 to 6.15)	0.842
	2W	+1.41 (−3.92 to 6.74)	0.604
	3W	−2.43 (−7.76 to 2.90)	0.371
**Triglycerides (mg/dL)**	OD	+15.71 (−63.35 to 94.78)	0.697

	Treatment group	Mean change from baseline to week 48 (95% CI)	p value
	1W	+31.05 (−60.84 to 122.94)	0.508
	2W	−10.85 (−84.53 to 62.83)	0.773
	3W	−1.33 (−75.01 to 72.35)	0.972
**AST (U/L)**	OD	−5.74 (−14.79 to 3.31)	0.214
	1W	−5.34 (−15.72 to 5.04)	0.313
	2W	+4.70 (−3.75 to 13.14)	0.276
	3W	+2.90 (−5.80 to 11.60)	0.514
**ALT (U/L)**	OD	−8.45 (−21.79 to 4.90)	0.215
	1W	−5.46 (−20.80 to 9.88)	0.485
	2W	−10.26 (−23.40 to 2.88)	0.126
	3W	−1.61 (−14.06 to 10.84)	0.800
**GGT (U/L)**	OD	+2.08 (−2.63 to 6.79)	0.387
	1W	+4.44 (−1.17 to 10.04)	0.121
	2W	−0.48 (−5.51 to 4.54)	0.851
	3W	+1.28 (−3.11 to 5.66)	0.569
**Alkaline phosphatase (U/L)**	OD	−3.64 (−12.55 to 5.26)	0.423
	1W	−7.00 (−17.51 to 3.51)	0.192
	2W	−24.91 (−33.71 to −16.10)	<0.001
	3W	−6.69 (−14.99 to 1.61)	0.114
**Total bilirubin (mg/dL)**	OD	−0.07 (−0.33 to 0.19)	0.588
	1W	+0.39 (0.09 to 0.69)	0.011
	2W	+0.07 (−0.17 to 0.31)	0.572
	3W	−0.03 (−0.27 to 0.21)	0.807
**Sodium (mmol/L)**	OD	+1.07 (−0.31 to 2.45)	0.128
	1W	+1.89 (0.30 to 3.47)	0.019
	2W	+0.02 (−1.26 to 1.31)	0.971

	Treatment group	Mean change from baseline to week 48 (95% CI)	p value
	3W	+0.64 (−0.65 to 1.92)	0.333
**Potassium (mmol/L)**	OD	−0.11 (−0.32 to 0.10)	0.291
	1W	+0.07 (−0.17 to 0.31)	0.588
	2W	−0.13 (−0.33 to 0.06)	0.172
	3W	+0.04 (−0.16 to 0.23)	0.716

Data are estimated using mixed-effects models. Results are presented as mean change from baseline to week 48 with 95% confidence intervals, except for CKD-EPI, which is presented as a ratio estimate with 95% confidence intervals.
OD = once daily; 1W = once weekly; 2W = twice weekly; 3W = three times weekly.

Supplementary Table 10. Detectability of inflammatory markers (IL-6 and hsCRP) at baseline and week 48, by treatment group

	OD (n=9)	1W (n=10)	2W (n=10)	3W (n=10)	Total
**IL-6 detectable (≥2 pg/mL)**					
Baseline	0 (0%)	1 (10%)	4 (40%)	4 (40%)	9 (24%)
Week 48	1 (11%)	0 (0%)	3 (33%)	0 (0%)	4 (11%)
**IL-6 <LLOQ (<2 pg/mL)**					
Baseline	8 (89%)	9 (90%)	6 (60%)	6 (60%)	29 (76%)
Week 48	8 (89%)	8 (100%)	6 (67%)	9 (100%)	31 (89%)
**hsCRP detectable (≥0.02 mg/dL)**					
Baseline	6 (75%)	9 (90%)	10 (100%)	9 (90%)	34 (89%)
Week 48	8 (89%)	8 (100%)	9 (100%)	9 (100%)	34 (97%)
**hsCRP <LLOQ (<0.02 mg/dL)**					
Baseline	2 (25%)	1 (10%)	0 (0%)	1 (10%)	4 (11%)
Week 48	1 (11%)	0 (0%)	0 (0%)	0 (0%)	1 (3%)

Data are n (%). Percentages are calculated within treatment group. Detectability defined as ≥ lower limit of quantification (LLOQ): 2 pg/mL for IL-6 and 0.02 mg/dL for hsCRP. OD = once-daily; 3W = three-times-per-week; 2W = twice-per-week; 1W = once-per-week.

Supplementary Table 11. Proportion of participants with good sleep quality (PSQI ≤5), by treatment group and visit

	OD (n=8)	1W (n=7)	2W (n=9)	3W (n=10)	Total
Baseline	2 (25%)	3 (43%)	2 (29%)	1 (14%)	8 (28%)
Week 4	NA	5 (50%)	5 (62%)	2 (25%)	12 (46%)
Week 12	3 (43%)	4 (57%)	6 (67%)	2 (20%)	15 (45%)
Week 24	2 (33%)	3 (57%)	5 (50%)	2 (22%)	12 (41%)
Week 36	4 (57%)	4 (80%)	4 (50%)	2 (29%)	14 (52%)
Week 48	4 (57%)	3 (86%)	6 (67%)	3 (43%)	19 (63%)

Data are n (% within treatment group). Poor sleep quality corresponds to Pittsburg Sleep Quality Index (PSQI) >5.
OD=once daily; 1W=once weekly; 2W=twice weekly; 3W=three times weekly. NA=not applicable.

Supplementary Table 12. Health-related quality of life assessed by EQ-5D-5L and EQ-VAS, by treatment group and visit

	Visit	OD (n=8)	1W (n=10)	2W (n=10)	3W (n=10)	Total
**EQ-5D-5L domains: participants reporting no problems, n (% within group)**						
Mobility	Baseline	8 (100%)	10 (100%)	8 (80%)	10 (100%)	36 (95%)
	Week 48	8 (100%)	6 (100%)	9 (100%)	8 (100%)	31 (100%)
Self-care	Baseline	8 (100%)	10 (100%)	10 (100%)	10 (100%)	38 (100%)
	Week 48	8 (100%)	6 (100%)	9 (100%)	8 (100%)	31 (100%)
Usual activities	Baseline	8 (100%)	10 (100%)	9 (90%)	10 (100%)	37 (97%)
	Week 48	8 (100%)	6 (100%)	9 (100%)	8 (100%)	31 (100%)
Pain / discomfort	Baseline	5 (63%)	10 (100%)	6 (60%)	10 (100%)	31 (82%)
	Week 48	7 (86%)	5 (86%)	8 (89%)	8 (100%)	28 (90%)
Anxiety / depression	Baseline	5 (63%)	8 (80%)	4 (40%)	8 (80%)	25 (64%)
	Week 48	8 (100%)	6 (100%)	8 (89%)	8 (100%)	30 (97%)
**EQ-VAS score, mean (SD) [n]**						
EQ-VAS (0–100 scale)	Baseline	87.6 (16.0)	74.0 (12.0)	88.2 (12.2)	83.4 (13.4)	86.2 (8.3)
	Week 48	87.5 (9.4)	81.1 (16.2)	88.6 (8.2)	84.6 (11.4)	82.5 (8.9)

Data are n (% within treatment group) for EQ-5D-5L domains and mean (SD) [n] for EQ-VAS. Higher EQ-VAS scores indicate better perceived health status.
OD = once daily; 1W = once weekly; 2W = twice weekly; 3W = three times weekly.

Supplementary Figure 1A. Ultrasensitive plasma HIV-1 RNA levels (copies per mL) at baseline and week 48. Boxes represent median and interquartile range, whiskers indicate adjacent values, and dots represent outlier values.

OD=once daily. 3W=three times weekly. 2W=twice weekly. 1W=once weekly.

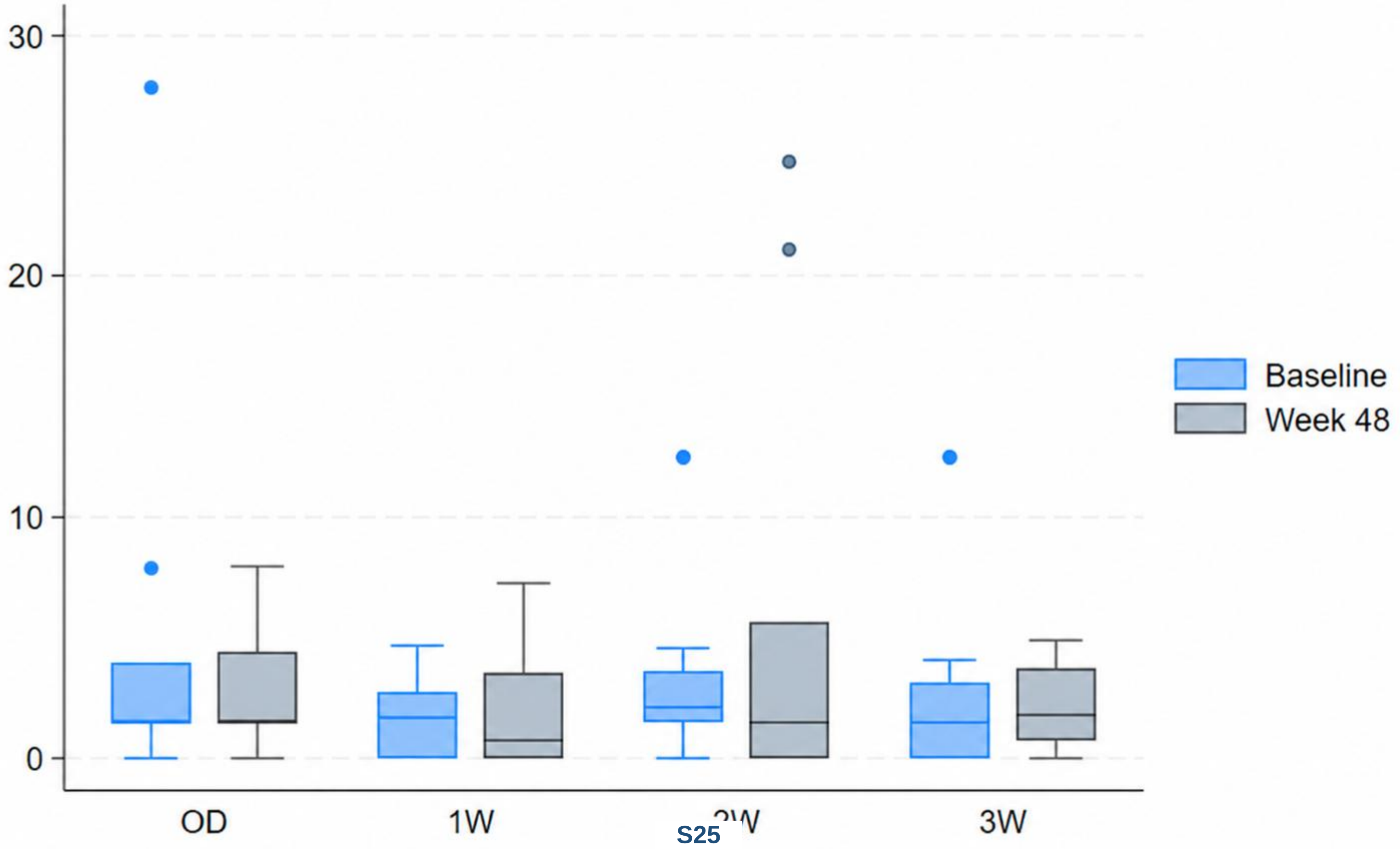

Supplementary Figure 1B. Distribution of ultrasensitive plasma HIV-1 RNA categories at baseline and week 48. Categories were defined as: <5 copies/mL, no signal; <5 copies/mL, signal detected; and ≥5 copies/mL. OD=once daily. 3W=three times weekly. 2W=twice weekly. 1W=once weekly.

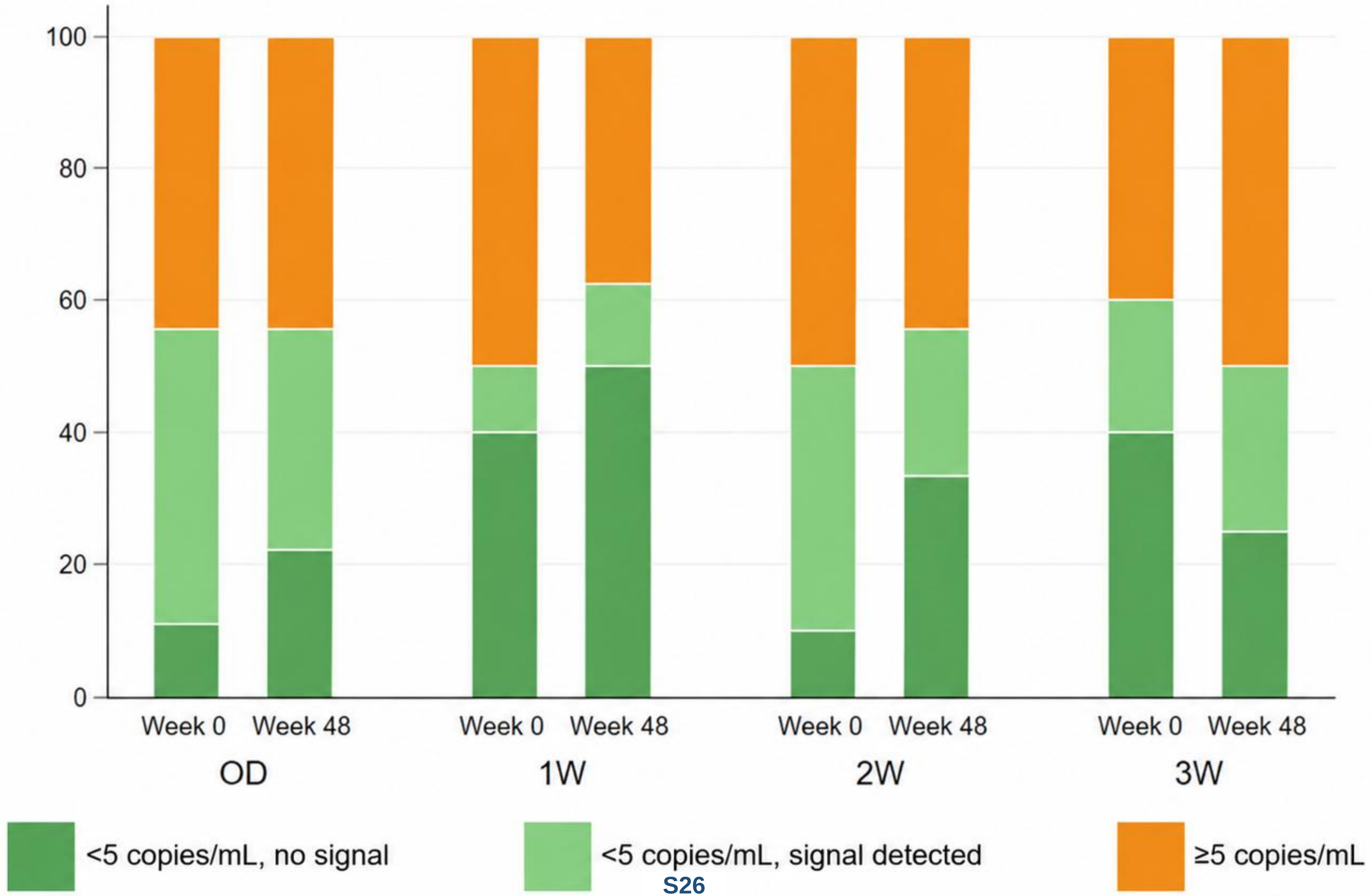

Supplementary Figure 2. Receiver operating characteristic (ROC) curves for pharmacokinetic predictors of detectable viraemia.

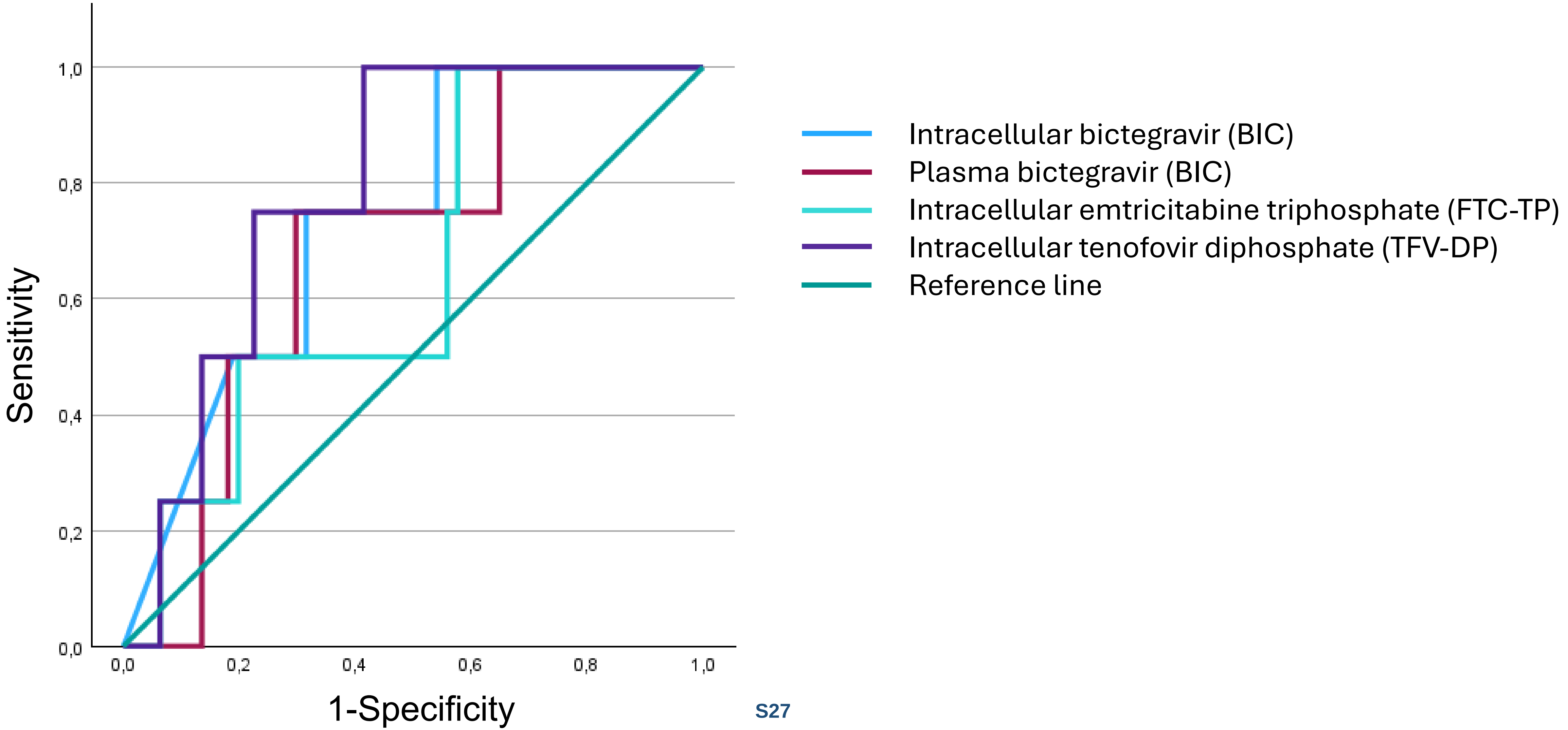

CLINICAL TRIAL PROTOCOL

**Protocol title:**	Safety, tolerability, and efficacy of a dose reduction strategy based on bictegravir/emtricitabine/tenofovir alafenamide in virologically suppressed HIV-infected adults.
**Protocol code:**	BETAF-RED
**Version and date**	1.1, 10-04-2022
**EudraCT**	2022-000358-26
**Sponsor**	Institut d'Investigacions Biomèdiques August Pi I Sunyer<br>–IDIBAPS-<br>Barcelona, Spain
**Principal investigator**	Dr. Esteban Martinez Chamorro<br>Infectious Diseases Service<br>Villarroel, 170<br>Barcelona

CONTACT DETAILS

Sponsor: Institut d'Investigacions Biomèdiques August Pi i Sunyer (IDIBAPS)

Principal Investigator:
Dr. Esteban Martinez Chamorro
Infectious Diseases Service
Hospital Clínic
Villarroel, 170
Barcelona

Other Hospital Clinic Investigators:
Pilar Callau
Ana Rodriguez
Iván Chivite
Berta Torres
Jose Luis Blanco
Mar Mosquera
José Alcamí
Sonsoles Sanchez Palomino
Cristina Rovira
Carmen Hurtado
Infectious Diseases Service
Hospital Clínic
Villarroel, 170
Barcelona

Investigators from other Centers:
Antonio D'Avolio
Amadeo Di Nicolo
University of Turin (Italy)

Pharmacovigilance: Clinical Trials Unit (CTU Clinic)

Statistician: Elisa de Lazzari

Local sample storage in Spain:
Cristina Rovira
Laboratori Retrovirologia i Immunopatogenia del VIH
Fundació Privada Clínic – IDIBAPS
CELLEX building, Planta 4ª Sector A
Facultat de Medicina UB
Barcelona-Spain
e-mail: crovira@clinic.cat

Pharmacokinetics samples storage facility:

Laboratory responsible:
Prof. Antonio D'Avolio (BSc, MSc, SM)
Head of the Laboratory of Clinical Pharmacology and Pharmacogenetics

Padiglione Q;
Amedeo di Savoia Hospital
C.so Svizzera 164, 10149
Torino (Italy)
Tel. Seg. +39.011.4393979
Fax: +39.011.4393996
Tel. Lab. +39.011.4393867, Uff. +39.011.4393841
e-mail: antonio.davolio@unito.it

SPONSOR SIGNATURE

I have read and approved this protocol. My signature, in conjunction with the signature of the investigator, confirms the agreement of both parties that the clinical study will be conducted in accordance with the protocol and all applicable local laws and regulations including, but not limited to, the International Conference on Harmonization Guideline for Good Clinical Practice (ICH GCP), the ethical principles that have their origin in the Declaration of Helsinki and applicable privacy laws.

Name of person who signs on behalf of the sponsor:
Signature:
Date:

INVESTIGATOR PROTOCOL AGREEMENT PAGE

I have thoroughly read and reviewed the trial protocol. I agree:

- To conduct the trial in accordance with GCP and the applicable regulatory requirements and with the approved protocol.

- That I am thoroughly familiar with the appropriate use of the investigational medicinal products, as described in the Investigator's Brochure and any other information provided by the Sponsor.

- To comply with the procedures for data recording /reporting.

- To ensure that all persons assisting me with the study are adequately informed about the study and of their study-related duties and functions.

- To permit monitoring, auditing and inspection and to retain the trial related essential documents for the period of time required according to ICH-GCP and local regulatory requirements.

Name of Principal Investigator: Esteban Martinez

Date: 10-04-2022

Contenido

CLINICAL TRIAL PROTOCOL 1

1. INTRODUCTION AND RATIONALE 13
2. OBJECTIVES OF THE CLINICAL TRIAL..18
3. TRIAL DESIGN 19
4. SUBJECT SELECTION AND WITHDRAWAL...23
5. TREATMENT OF SUBJECTS...25
6. VISIT SCHEDULE AND STUDY PROCEDURES 27
7. STATISTICAL ANALYSIS 32
8. SAFETY ASSESSMENT 34
9. DATA HANDLING 40
10. QUALITY CONTROL AND QUALITY ASSURANCE 42
11. ETHICAL AND REGULATORY CONSIDERATIONS..43
12. FINANCING AND INSURANCE 47
13. PUBLICATION 47

Appendix 1. Declaración de Helsinki
Appendix 2. Spanish Specific Questionnaries
Appendix 3. Pharmacocinetic Samples.

ABBREVIATIONS

ADR	Adverse Drug Reaction
AE	Adverse Event
AR	Adverse reaction
AST	Aspartate aminotransferase
BETAF	fixed combination of bictegravir/emtricitabine/TAF
DSMB	Data Safety Monitoring Board
EMA	European Medicines Agency
GCP	Good Clinical Practice
HCV	Hepatitis C Virus
HBV	Hepatitis B virus
HIV-1	Human immunodeficency virus type 1
ICF	Informed Consent Form
ICH	International Conference on Harmonization
FCRB	Fundació Clinic per a la Recerca Biomèdica
IMP	Investigational Medicinal Products
ISF	Investigator Site File
OD	Once-daily
SAE	Serious Adverse Events
SAP	Statistical Analysis Plan
SUSAR	Suspected Unexpected Serious Adverse Reaction
TMF	Trial master file
UAR	Unexpected Adverse Reaction
3W	3 days (Mondays, Wednesdays, and Fridays) a week
2 W	2 days a week
1 W	1 day a week

PROTOCOL SYNOPSIS

**Title of the study**	Safety, tolerability, and efficacy of a dose reduction strategy based on bictegravir/emtricitabine/tenofovir alafenamide in virologically suppressed HIV-infected adults.
**Protocol code**	BETAF-RED
**EudraCT No.**	2022-000358-26
**Sites**	Unicenter clinical trial:<br>Hospital Clinic-Fundació Clínic, Barcelona, Spain
**Study period**	48 weeks
**Primary objectives**	1. Viral efficacy (standard plasma viral load, lower limit of detection HIV RNA 50 copies/mL) of the reduction of BETAF regimen dose per week at 12 weeks.<br>2. Viral efficacy (standard plasma viral load, lower limit of detection HIV RNA 50 copies/mL) of the reduction of BETAF regimen dose per week at 48 weeks.
**Secondary objectives**	1. Virologial efficacy:<br>a. Standard plasma viral load, lower limit of detection HIV RNA 50 copies/mL) at 4, 24, and 36 weeks<br>b. Blips (VL ≥50 copies/mL followed by VL <50 copies/mL) at 4, 12, 24, 36, and 48 weeks.<br>c. Target not detected with standard plasma viral load (lower limit of detection HIV RNA 50 copies/mL) at 0, 4, 12, 24, 36, and 48 weeks<br>d. Ultrasensitive plasma viral load (lower limit of detection 5 copies/mL) at 0, 12, and 48 weeks<br>**e.** HIV-1 reservoir (total and integrated DNA) in CD4 cells at 0, 12, and 48 weeks.<br>f. In case of virological failure, ultra-deep sequencing of plasma and intracellular viral load to detect genotypic resistance<br>2. Immunological safety:<br>a. CD4 and CD8 cells, CD4/CD8 ratio, inflammation (hsCRP, IL-6, adiponectin) and immune activation (sCD14, CD163) markers at 0, 12, and 48 weeks.<br>3. Subclinical toxicity:

	a. Cummulated incidental adverse events at 48 weeks<br>b. Weight and body mass index (BMI) changes at 4, 12, 24, 36, and 48 weeks<br>c. Body composition (fat, fat-free mass, and bone by DEXA) at 0 and 48 weeks<br>d. Impact on sleep quality (Pittsburg Sleep Quality Index)<br>e. Impact on quality of life (EQ-5D-5L)<br><br>4. Pharmacokinetics of antiretroviral drugs at baseline and at 4, and 48 weeks:<br>a. Plasma levels of bictegravir, emtricitabine and tenofovir<br>b. Intracellular levels of bictegravir and phosphorylated emtricitabine and tenofovir.
**Study population**	Virologically suppressed (plasma HIV RNA <50 copies/mL) HIV-infected adults (≥18 years) on BETAF once daily
**Study Design**	Open-label, unicenter, national, randomized, pilot trial
**Number of subjects**	40 subjects
**Inclusion Criteria**	1. Stable and asymptomatic HIV-infected adults (≥18 years) on BETAF once daily for at least the previous 6 months.<br>2. Plasma HIV-1 RNA less than 50 copies/mL for at least the previous 6 months<br>3. CD4 cell counts greater than 350 cells/mL at the time of consideration for the study<br>4. Women of child-bearing potential must have a negative pregnancy test in serum before the inclusion in the study and agree to use highly effective contraceptive methods<br>5. Patients agree to participate
**Exclusion criteria**	1. Prior virological failure to any antiretroviral regimen or documented resistance mutations to bictegravir, emtricitabine, or tenofovir<br>2. Any diagnosis of psychiatric illness<br>3. Alcohol abuse or illicit drug consumption (based on their past medical history and specific questions at the time of recruitment)

	4. Patients co-infected with HIV and active hepatitis B or C virus<br><br>5. Any other condition at the doctor's discretion that did not allow ensuring a correct adherence.
**Investigational Product**	BIKTARVY ®(Bictegravir/FTC/TAF)
**Route and dosage form**	Oral administration
**Dosage**	BIKTARVY is a three-drug fixed dose combination product containing 50 mg of bictegravir (BIC), 200 mg of emtricitabine (FTC), and 25 mg of tenofovir alafenamide (TAF).<br><br>The recommended dosage of BIKTARVY is one tablet taken orally once daily with or without food in adults
**Arms**	1. (OD arm): one tablet taken orally once daily (OD arm)<br><br>2. (3W arm): one tablet taken orally 3 days per week (3W arm) : Mondays, Wednesdays, and Fridays<br><br>3. (2W arm): one tablet taken orally 2 days per week (2W arm): Mondays, and Thursdays<br><br>4. (1W arm): one tablet taken orally 1 days per week (1W arm): Mondays
**Duration of treatment**	The duration of the study treatment will be 48 weeks
**Outcome measures**	Primary outcomes will be defined as:<br>- Patients with VL <50 copies at 12 weeks (FDA snapshot algorithm) in the intention-to-treat (ITT) population.<br><br>- Patients with VL <50 copies at 48 weeks (FDA snapshot algorithm) in the intention-to-treat (ITT) population.
**Secondary and exploratory outcome measures**	1. Virological efficacy:<br>a. Standard plasma viral load, lower limit of detection HIV RNA 50 copies/mL) at 4, 24, and 36 weeks<br><br>b. Blips (VL ≥50 copies/mL followed by VL <50 copies/mL) at 4, 12, 24, 36, and 48 weeks.<br><br>c. Target not detected with standard plasma viral load (lower limit of detection HIV RNA 50 copies/mL) at 0, 4, 12, 24, 36, and 48 weeks<br><br>d. Ultrasensitive plasma viral load (lower limit of detection 5 copies/mL) at 0, 12, and 48 weeks<br><br>e. HIV-1 reservoir (total and integrated DNA) in CD4 cells at 0,

	12, and 48 weeks.<br>f. In case of virological failure, ultra-deep sequencing of plasma and intracellular viral load to detect genotypic resistance<br>2. <u>Immunological safety</u>:<br>a. CD4 and CD8 cells, CD4/CD8 ratio at 0, 12, and 48 weeks<br>b. Inflammation (hsCRP, IL-6, adiponectin) and immune activation (sCD14, CD163) markers at 0, 12, and 48 weeks<br>3. <u>Subclinical toxicity</u>:<br>a. Cummulated incidental adverse events at 48 weeks<br>b. Weight and body mass index (BMI) changes at 4, 12, 24, 36, and 48 weeks<br>c. Body composition (fat, fat-free mass, and bone by DEXA,) at 0 and 48 weeks<br>d. Impact on sleep quality (Pittsburg Sleep Quality Index) at 0, 4, 12, 24, 36, and 48 weeks<br>e. Impact on quality of life (EQ-5D-5L) at 0 and 4,12,24,36, 48 weeks<br>4. <u>Pharmacokinetics of antiretroviral drugs at baseline and at 12 and 48 weeks</u>:<br>a. Plamas levels of bictegravir, emtricitabine and tenofovir<br>b. Intracellular levels of bictegravir, phosphorylated emtricitabine and phosphorylated tenofovir.
**Statistical Methods**	Summary statistics will be performed using absolute frequency and percentages for qualitative variables and mean and SD or median and IQR for quantitative characteristics, at each visit. The primary and the secondary virological outcomes will be reported as absolute frequency and percentage along with the exact 95%CI, for each trial arm and visit. Laboratory parameters, weight, BMI and dexa parameters will be described with median and IQR for each trial arm and visit. Items of PSQI and EQ-5D-5L questionnaires will be reported with absolute frequency and percentage. Incidence of AEs will be reported as number of events per 100/person-month.<br><br>Under descriptive/exploratory purposes, the virological outcomes will be compared between groups using the Fisher's exact test. Laboratory parameters and dexa will be compared between study arms and follow-up visits by means the mixed-effects regression model. They will be log-transformed in case of non-normal residual

	distribution and a description with geometric mean and 95%CI will be reported too.<br><br>Incidence rate of AEs will be compared between arms using the Poisson (or negative binomial) regression model. All tests will be two-tailes with a significance level set at 0.05

1. INTRODUCTION AND RATIONALE

With widespread access to effective antiretroviral therapy, a majority of HIV-infected patients can nowadays achieve sustained viral suppression [1]. However, antiretroviral therapy should be indefinitely taken and the associated risk of long-term chronic toxicities and/or negative impact on aging comorbidities increases with duration of exposure. In fact, these reasons are now the most common ones leading to changes in antiretroviral therapy in developed countries [2].

Because of suboptimal efficacy and limited number, antiretroviral drugs have been usually approved at the maximum tolerated dose rather than the minimum effective dose [3]. There is a clear relationship between plasma levels of some antiretroviral drugs such as tenofovir [4], efavirenz (EFV) [5], or dolutegravir (DTG) [6] and their potential for developing drug-related toxicities. Reducing the daily dose of EFV from 600 to 400 mg in antiretroviral-naive patients [7, 8], using tenofovir alafenamide (TAF) instead of tenofovir disoproxil fumarate (TDF) [9, 10], or administering DTG dose in the morning to decrease plasma DTG concentration at night [11] are strategies decreasing exposure to EFV, tenofovir, or DTG respectively, aimed to provide similar efficacy yet better safety profile.

Antiretroviral therapy has evolved to a larger number of families available and drugs with more convenient dosing schemes and better overall tolerability. The construction of fixed-drug combinations with half-lives long enough to allow for once-daily dosing established the basis for preferred antiretroviral regimens in the last decade. Triple regimens based on integrase inhibitors are currently recommended as first options worldwide [12-15]. The combination of bictegravir/emtricitabine/tenofovir alafenamide (BETAF) has been the latest single-table triple regimen incorporated into the therapeutic armamentarium. In Spain, BETAF is one of the most commonly used regimens in antiretroviral-naive patients and it is widely used also for simplification purposes in virologically treated HIV-positive adults. In contrast with other preferred regimens which are contraindicated in persons with positive HLA-B 5701 (dolutegravir/abacavir/lamivudine) or in persons with chronic hepatitis B (dolutegravir/abacavir/lamivudine, dolutegravir/lamivudine), there are no specific limitations or restrictions with BETAF. The Hospital Clínic cohort currently cares for more than 5000 active HIV-positive adults, of whom approximately 40% are receiving BETAF. Despite BETAF being one of the best tolerated regimens, current data suggests that bictegravir may be associated with central nervous system adverse effects although less commonly than dolutegravir [16], TAF with higher plasma lipids than TDF [17], and both bictegravir and TAF with higher weight gain [18] although the pathogenesis and risk factors of these potential adverse effects are currently unknown. BETAF is one of the simplest available regimens, but there may be further room for improvement. Each one of the individual components of BETAF has among the longest half-lives of all currently recommended antiretrovirals: 39 hours for intracellular emtricitabine triphosphate [19], 150-180 hours for intracellular tenofovir diphosphate with TDF [20], and 163 hours for the dissociation of bictegravir from wild-type HIV-1 integrase-DNA complex [21]. Moreover, more than 2-fold higher intracellular tenofovir diphosphate concentrations can be achieved with TAF relative to TDF [22]. These pharmacokinetic characteristics could allow for longer than once-daily dosing. Missing up to 4 doses of BETAF in *in-vitro* culture models seems safe in terms of breakthrough and resistance development [23, 24]. *In vitro* experiments mimicking missing one or two doses a weeks for 5 weeks did not show any viral breakthrough; the same experiments

missing three or four doses a week for 5 weeks showed the possibility for viral breakthrough but at no expense of major resistance development [23, 24]. These are *in vitro* data and, although oritentative, cannot be extrapolated to *in vivo*. We have followed two patients who decided by themselves to take BETAF either twice a week or once a week and both have maintained HIV-1 RNA below 50 copies/mL for more than six months [unpublished data]. The high genetic barrier of DETAF has been confirmed with the absence of emergent resistance in clinical trials [25] even in patients with archived resistance [26]. Finally, studies on viral rebound after antiretroviral therapy interruption have shown that it may take 2-3 weeks to have detectable HIV RNA in plasma [27]. Recently, a French randomized clinical trial showed that 4-days on and 3-days off maintenance antiretroviral therapy showed non-inferiority with daily standard triple antiretroviral therapy after one year of follow-up [28]. For all these reasons, we believe that there is a sound rationale to test longer than once daily BETAF dosing as we propose in this study.

Seminal studies in virally suppressed HIV-infected patients showed a few years ago that that short-cycle 5 days on/2 days off intermittent treatment with EFV and TDF plus other nucleoside reverse transcriptase inhibitors with long half-lives such as 3TC or ddI [29] or EFV/FTC/TDF [30] were feasible and able to maintain viral suppression. One of these studies has recently published 4-year follow-up data showing impressive results [31]. Our group has previously performed a pilot study with the support of Instituto de Salud Carlos III (Convocatoria de Ayudas de Proyectos de Investigación en Salud, Project number PI12/01217) showing that three-day per week EFV/FTC/TDF in patients with sustained viral suppression was a feasible option that maintained efficacy and improved subclinical toxicity parametres at 24 weeks. None of the participants with three-day per week EFV/FTC/TDF had viral failure, and their ultrasensitive plasma viral load, HIV-1 reservoir in peripheral blood mononuclear cells, and several in-depth immunological parameters remained similarly stable as compared with patients on daily EFV/FTC/TDF [32, 33]. Moreover, reduction of drug exposure for 24 weeks only had a significant positive impact on parameters reflecting toxicities related with efavirenz or tenofovir such as sleep quality, bone mineral density, and proteinuria despite the patients included (by inclusion criteria) were asymptomatic and had no laboratory evidence of toxicity. Due to the extremely good results after the first 24 weeks, an extension of the study was approved and all participants were switched to three-day per week EFV/FTC/TDF and followed for more than 3 years. In such a long period, nopatient has dveloped virological failure [data submitted for publication]. Our study represented a proof of concept that has been later confirmed in a larger independent clinical trial [34].

Standard (lower limit of detection 50 copies/mL) plasma HIV RNA assessment and CD4 and CD8 cells have been so far the tools commonly used for research and clinical care purposes. However, new treatment simplification strategies might need more sophisticated laboratory tools to further confirm efficacy when compared with standard triple antiretroviral therapy [35, 36]. For this reason, we propose to measure ultrasensitive plasma viral load, HIV-1 reservoir in peripheral blood mononuclear cells, and several key inflammation and immune activation markers.

In addition to proving efficacy, from an ethical perspective any therapy simplification strategy has to offer additional advantages to patients [37]. We asume that reduction of drug exposure will have a significant positive impact on parameters reflecting potential toxicities associated with bictegravir or tenofovir. For this reason, we propose to measure changes in regional fat and fat-free mass

(DEXA), bone mineral density (DEXA), quality of life (EQ-5D-5L), and sleep quality (Pittsburg Sleep Quality Index, PSQI).

It makes sense to generate pharmacology data with reduced dose schedules with BETAF. These pharmacokinetic data will support further the expected efficacy and safety of the reduced dose BETAF schedule and will be useful for future studies. For this reason, we also propose to measure plasma and intracellular bictegravir, tenofovir, and emtricitabine levels.

REFERENCES

1. O'Connor J, et al. Durability of viral suppression with first-line antiretroviral therapy in patients with HIV in the UK: an observational study. Lancet HIV 2017; 4: e295-e302

2. Di Biagio A, et al. Treatment discontinuation in HIV-1-infected individuals starting their first-line HAART after 2008: data from the ICONA Foundation Study Cohort. J Int AIDS Soc 2014; 17(Suppl 3): 19825
3. Vozza L, et al. How medicines are born: the imperfect science of drugs. London ; Hackensack, NJ : World Scientific Publishing Europe Ltd., 2017

4. Baxi SM, et al. Common clinical conditions -age, low BMI, ritonavir use, mild renal impairment- affect tenofovir pharmacokinetics in a large cohort of HIV-infected women. AIDS 2014; 28: 59-66
5. Gutierrez F, et al. Prediction of neuropsychiatric adverse events associated with long-term efavirenz therapy, using plasma drug level monitoring. Clin Infect Dis 2005; 41: 1648-1653

6. Yagura, H., H. et al. Impact of UGT1A1 gene polymorphisms on plasma dolutegravir trough concentrations and neuropsychiatric adverse events in Japanese individuals infected with HIV-1. BMC Infect Dis 2017; 17: 622

7. ENCORE1 Study Group. Efficacy of 400 mg efavirenz versus standard 600 mg dose in HIV-infected, antiretroviral-naive adults (ENCORE1): a randomised, double-blind, placebo-controlled, non-inferiority trial. Lancet 2014; 383: 1474-1482

8. ENCORE1 Study Group. Efficacy and safety of efavirenz 400 mg daily versus 600 mg daily: 96-week data from the randomised, double-blind, placebo-controlled, non-inferiority ENCORE1 study. Lancet Infect Dis 2015; 15: 793-802

9. Sax PE, I, et al. Tenofovir alafenamide vs. tenofovir disoproxil fumarate in single tablet regimens for initial HIV-1 therapy: a randomized phase 2 study. J Acquir Immune Defic Syndr 2014; 67: 52-58

10. Mills A, et al. Tenofovir alafenamide versus tenofovir disoproxil fumarate in the first protease inhibitor-based single-tablet regimen for initial HIV-1 therapy: a randomized phase 2 study. J Acquir Immune Defic Syndr 2015; 69: 439-445

11. Capetti A, et al. Morning dosing for dolutegravir-related insomnia and sleep disorders. HIV Medicine 2017; 19: e62 -e63

12. Borrador del documento de consenso de Gesida/Plan Nacional sobre el Sida respecto al tratamiento antirretroviral en adultos infectados por el virus de la inmunodeficiencia humana (Actualización enero 2020). In: http://gesida-seimc.org/category/guias-clinicas/borradores

13. European AIDS Clinical Society (EACS) Guidelines Version 11.0 November 2021. In: https://www.eacsociety.org/media/final2021eacsguidelinesv11.0_oct2021.pdf

14. Guidelines for the Use of Antiretroviral Agents in Adults and Adolescents with HIV. Department of Health and Human Services (updated December 18, 2019). In: https://aidsinfo.nih.gov/contentfiles/lvguidelines/AdultandAdolescentGL.pdf

15. Update of Recommendations on First- and Second-Line Antiretroviral Regimens. World Health Organization 2019. In: https://apps.who.int/iris/bitstream/handle/10665/325892/WHO-CDS-HIV-19.15-eng.pdf?ua=1

16. Stellbrink HJ, et al. Co-formulated bictegravir, emtricitabine, and tenofovir alafenamide versus dolutegravir with emtricitabine and tenofovir alafenamide for initial treatment of HIV-1 infection: week 96 results from a randomised, double-blind, multicentre, phase 3, non-inferiority trial. Lancet HIV 2019; 6: e364 -72

17. Mallon PW, et al. Changes in lipids after a direct switch from TDF to TAF. CROI 2019, Seattle: abstract 652

18. Sax PE, et al. Weight gain following initiation of antiretroviral therapy: risk factors in randomized comparative clinical trials. Clin Infect Dis 2019 (published ahead of print)

19. Wang L, et al. Pharmacokinetic and pharmacodynamic characteristics of emtricitabine support its once daily dosing for the treatment of HIV infection. AIDS Res Hum Retroviruses 2004; 20: 1173-1182

20. Hawkins T, et al. Intracellular pharmacokinetics of tenofovir diphosphate, carbovir triphosphate, and lamivudine triphosphate in patients receiving triple-nucleoside regimens. J Acquir Immune Defic Syndr 2005; 39: 406-411

21. White KL et al. Long dissociation of bictegravir from HIV-1 integrase-DNA complexes. Antimicrob Agents Chemother 2021; 65: e02406-20

22. Podany AT, et al. Plasma and intracellular pharmacokinetics of tenofovir in patients switched from tenofovir disoproxil fumarate to tenofovir alafenamie. AIDS 2018; 32: 761-765

23. Mulato A et al. Forgiveness of BIC/FTC/TAF: In Vitro Simulations of Intermittent Poor Adherence Find Limited HIV-1 Breakthrough and High Barrier to Resistance. IDWeek 2020: poster 1448.

24. Acosta RK et al. In Vitro Forgiveness of INSTI-Containing Regimens at Drug Concentrations Simulating Variable Adherence. IDWeek 2021: poster 888.

25. Sax PE et al. Switching to bictegravir, emtricitabine, and tenofovir alafenamide in virologically suppressed adults with human immunodeficiency virus. 2021; 73: e485-e493

26. Andreatta K et al. Switching to vbictegravir/emtricitabine/tenofovir alafenamide maintained HIV-1 RNA suppression in participants with archived antiretroviral resistance including M184V/I. J Antimicrob Chemother 2019; 74: 3555-3564.

27. Li JZ et al. Time to viral rebound after interruption of modern antiretroviral therapies. Clin Infect Dis 2021Jun 12; ciab541. doi: 10.1093/cid/ciab541. Online ahead of print.

28. Landman R et al. A 4-days-on and 3-days-off maintenance treatment strategy for adults with HIV-1 (ANRS 170 QUATUOR): a randomised, open-label, multicentre, parallel, non-inferiority trial. Lancet HIV 2022; 9: e79-e90

29. Cohen C, et al. The FOTO study: the 48 week extension to assess durability of the strategy of taking efavirenz, tenofovir and emtricitabine five days on, two days off (FOTO) each week in virologically suppressed patients. 5th International AIDS Society Conference on HIV Pathogenesis, Treatment, and Prevention; Cape Town, South Africa; July 19 -22, 2009. Abstr MOPE B063

30. BREATHER (PENTA 16) Trial Group. Weekends-off efavirenz-based antiretroviral therapy in HIV-infected children, adolescents, and young adults (BREATHER): a randomised, open-label, non-inferiority, phase 2/3 trial. Lancet HIV 2016; 3: e421-e430

31. Turkova A, et al. Weekends-off efavirenz-based antiretroviral therapy in HIV-infected children, adolescents and young adults (BREATHER): Extended follow-up results of a randomised, open-label, non-inferiority trial. PLoS ONE 13(4): e0196239.

32. Rojas J, Blanco JL, Sanchez-Palomino S, et al. A maintenance 3-day-per-week schedule with the single tablet regimen efavirenz/emtricitabine/tenofovir disoproxil fumarate is effective and decreases sub-clinical toxicity. AIDS 2018; 32: 1633-1641

33. Guardo AC, Zarama A, González T, et al. Effects on immune system and viral reservoir of a short-cycle antiretroviral therapy in virologically suppressed HIV-positive patients. AIDS 2019; 33: 965-972

34. Bellagamba R, Giancola ML, Tommasi C, et al. Randomized clinical trial on efficacy of fixed-dose efavirenz/tenofovir/emtricitabine on alternate days versus continuous treatment. AIDS 2019; 33: 493-502

35. Liz JZ, Sax PE, Marconi VC, et al. No significant changes to residual viremia after switch to dolutegravir and lamivudine in a randomized trial. Open Forum Infect Dis 2019; 6: ofz056

36. Charpentier C, Peytavin G, Burdet, et al. Residual HIV-1 RNA, HIV-1 DNA, and drug plasma Cmin in dual DTG+3TC, ANRS 167 LAMIDOL. CROI 2019, Seattle: abstract 491].

37. Carr A, Hoy J, Pozniak A. The ethics of switch/simplify in antiretroviral trials: noninferior or just inferior? PLoS Med 2012; 9:e1001240.

2. OBJECTIVES OF THE CLINICAL TRIAL

The aim of this study is to assess the feasibility of dose redutions of Bictegravir/FTC/TAF in virologically suppressed HIV-infected adults on BETAF once daily .

Primary objectives

1. To assess viral efficacy (VL <50 copies) of the reductions of BETAF regimen dose at 12 weeks (on-treatment and intent-to-treat populations).
2. To asess viral efficacy of the reduction of BETAF regimen dose at 48 weeks (on-treatment and intent-to-treat populations).

Secondary objectives

1. Virological efficacy:
   - Standard plasma viral load, lower limit of detection HIV RNA 50 copies/mL) at 4, 24, and 36 weeks
   - Blips (VL ≥50 copies/mL followed by VL <50 copies/mL) at 4, 12, 24, 36, and 48 weeks.
   - Target not detected with standard plasma viral load (lower limit of detection HIV RNA 50 copies/mL) at 0, 4, 12, 24, 36, and 48 weeks
   - Ultrasensitive plasma viral load (lower limit of detection 5 copies/mL) at 0, 12, and 48 weeks
   - HIV-1 reservoir (total and integrated DNA) in CD4 cells at 0, 12, and 48 weeks.
   - In case of virological failure (confirmed HIV RNA ≥50 copies/mL in ≤2 weeks), ultra-deep sequencing of plasma and intracellular viral load to detect genotypic resistance

2. Immunological safety:

- CD4 and CD8 cells, CD4/CD8 ratio at 0, 12, and 48 weeks
- Inflammation (hsCRP, IL-6, adiponectin) and immune activation (sCD14, CD163) markers at 0, 12, and 48 weeks

3. Subclinical toxicity:
   - Cummulated incidental adverse events at 48 weeks
   - Weight and body mass index (BMI) changes at 4, 12, 24, 36, and 48 weeks
   - Body composition (fat, fat-free mass, and bone by DEXA) at 0 and 48 weeks
   - Impact on sleep quality (Pittsburg Sleep Quality Index) at 0, 4, 12, 24, 36and 48 weeks
   - Impact on quality of life (EQ-5D-5L) at 0, 4, 12, 24, 36 and 48 weeks

4. Pharmacokinetics of antiretroviral drugs at baseline and at 12, and 48 weeks
   - Plasma levels of bictegravir, emtricitabine and tenofovir

- Intracellular levels of bictegravir and phosphorylated emtricitabine and tenofovir .

3. TRIAL DESIGN

Primary and secondary endpoints

<u>Primary endpoints</u>

1. To assess viral efficacy (VL <50 copies) of the reduction of BETAF regimen dose at 12 weeks (on-treatment and intention-to-treat populations).
2. To assess viral efficacy of the reduction of BETAF regimen dose at 48 weeks (on-treatment and intention-to-treat populations)..

<u>Secondary endpoints</u>:

1. Virological efficacy:
   - Standard plasma viral load, lower limit of detection HIV RNA 50 copies/mL) at 4, 24, and 36 weeks
   - Blips (VL ≥50 copies/mL followed by VL <50 copies/mL) at 4, 12, 24, 36, and 48 weeks.
   - Target not detected with standard plasma viral load (lower limit of detection HIV RNA 50 copies/mL) at 0, 4, 12, 24, 36, and 48 weeks
   - Ultrasensitive plasma viral load (lower limit of detection 5 copies/mL) at 0, 4, and 48 weeks
   - HIV-1 reservoir (total and integrated DNA) in CD4 cells at 0, 12, and 48 weeks.
   - In case of virological failure, ultra-deep sequencing of plasma and intracellular viral load to detect genotypic resistance

2. Immunological safety:
   - CD4 and CD8 cells, CD4/CD8 ratio at 0, 12, and 48 weeks
   - Inflammation (hsCRP, IL-6, adiponectin) and immune activation (sCD14, CD163) markers at 0, 12, and 48 weeks

3. Subclinical toxicity:
   - Cummulated incidental adverse events at 48 weeks
   - Weight and body mass index (BMI) changes at 4, 12, 24, 36, and 48 weeks
   - Body composition (fat, fat-free mass, and bone by DEXA) at 0 and 48 weeks
   - Impact on sleep quality (Pittsburg Sleep Quality Index) at 0 and 4, 12, 24, 36, 48 weeks
   - Impact on quality of life (EQ-5D-5L) at 0 and 4, 12, 24, 36, 48 weeks

4. Pharmacokinetics of antiretroviral drugs at baseline and at 4, and 48 weeks
   - Plasma levels of bictegravir, emtricitabine and tenofovir
   - Intracellular levels of bictegravir and phosphorylated emtricitabine and tenofovir .

Study design.

This is a phase II, unicentric, open, pilot, randomized, controlled trial to evaluate Bictegravir/FTC/TAF.

The study will be developed at a single clinical care centre:

Hospital Clínic de Barcelona, Barcelona, Spain

Total follow-up will be 48 weeks.

Patients on BETAF regimen who meet inclusion criteria and none of the exclusion criteria will be included in this trial.

Treatment:

BIKTARVY® is a three-drug fixed dose combination product containing 50 mg of bictegravir (BIC), 200 mg of emtricitabine (FTC), and 25 mg of tenofovir alafenamide (TAF). The recommended dosage of BIKTARVY is one tablet taken orally once daily with or without food.

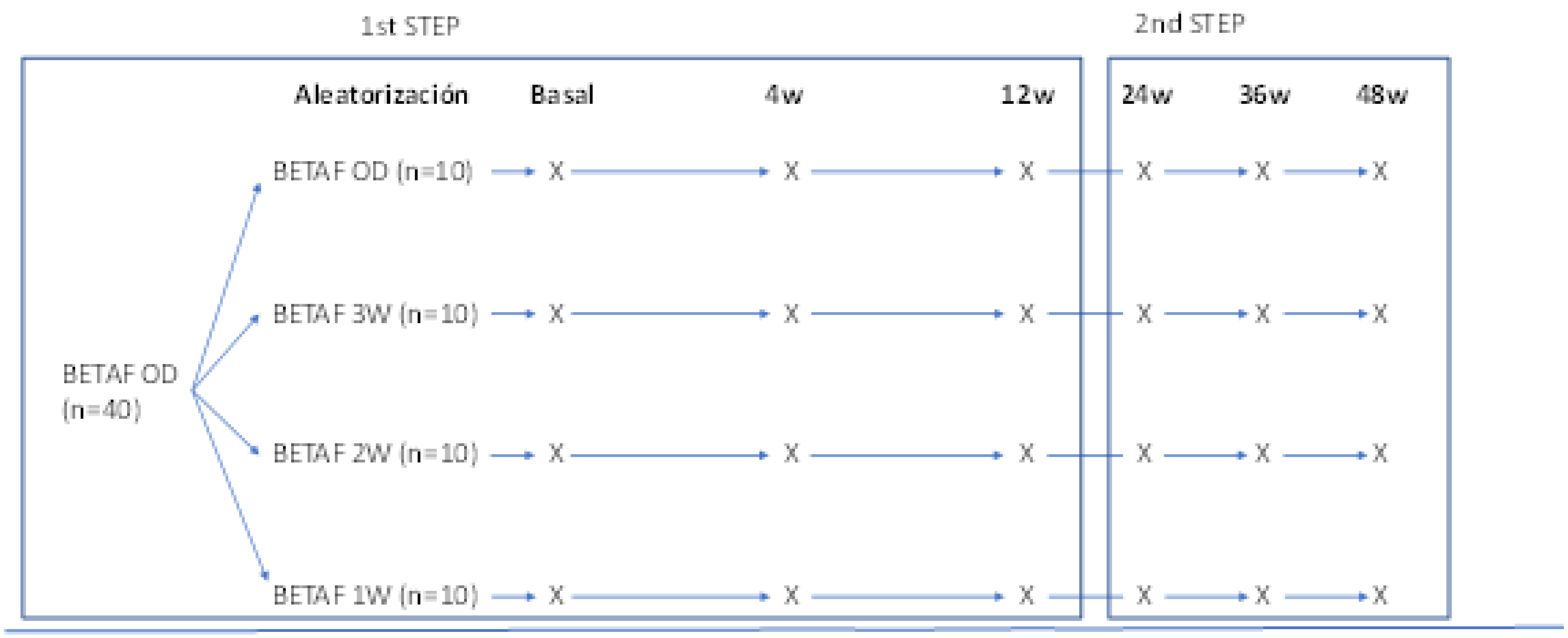

	Basal	4w	12w	24w	36w	48w
Visits/questionaries (QoL/PSQI)	X	X	X	X	X	X
Standart Analyses	X	X	X	X	X	X
especial analyses(Virol/Immunol)	X		X			X
DEXA orTAC abdominal	X					X
PK (plasma/células)	X		X			X
Virológical failure (if it occurs)		(X)	(X)	(X)	(X)	(X)

Figure 1. Flow-chart of the study

(BETAF OD arm): one tablet taken orally once daily

(BETAF 3W arm): one tablet taken orally 3 days per week : Mondays, Wednesdays, and Fridays

(BETAF 2W arm): one tablet taken orally 2 days per week : Mondays, and Thursdays

(BETAF 1W arm): one tablet taken orally 1 days per week : Mondays

Trial duration

The duration of the trial will be 48 weeks. The recruitment period will be 12 months.

Criteria for termination and/or discontinuation

Once participants start their allocated regimen, they will be reinforced to adhere to the corresponding schedule. Patients will be recommended to put alarm settings in their agendas, calendars, or smartphones at the initiation of the study. They will be visited at 4 weeks and plasma HIV RNA will be measured. All participants included will complete the first 4-week follow-up step.

Any participant from experimental arms with confirmed (≤2 weeks) detectable HIV RNA at any follow-up visit will have genotypic resistance studied and will be re-started on full daily Biktarvy dose and. Patients will continue follow-up until termination of the study at 48 weeks.

Any participant will discontinue study participation if he/she is unwilling or unable to meet the protocol requirements in terms of the visit schedule or if the patient or the investigator considers it is best to end their participation in the study. All participants have the right to withdraw their consent at any time during the study without any prejudice.
All follow-up terminations of study subjects and the reasons for them must be reported immediately to the study monitor and be duly documented both in the medical records and the case report form.

Stopping rules: If any experimental arm shows more than 30% on-treatment virological failure and more than 10% intent-to-treat virological failure, such an experimental arm will be stopped; patients not showing virological failure will be recommended to take BETAF daily and patients showing virological failure will have their regimen adjusted according to their best individual options.

Premature termination of the study will occur if all three experimental arms will need to be stopped. Premature termination will be reported to the local Ethics Committee and competent authorities in accordance with country specific requirements.

Once all patients have reached the 12-week primary end-point, a Data Safety Monitoring Board (DSMB) will be immediately convened to assess the efficacy and safety of each experimental arm. The following independent experts have been appointed for the DSMB: Dr. Joan Albert Arnaiz, Dr Francesc Vidal, and Dr. José Antonio Martínez

Drug accountability.

BIKTARVY® will be administered by the Pharmacy Service of the participating Center

Allocation participant identification code

All participants will be randomly allocated to one of the four treatment arms of the study and assigned with a subject number that will allow the patient to be identified. Subjects will be identified only by subject number in order to protect confidentiality. The site investigator will keep its own patient log to match the identification number with the patient’s personal data, in accordance with applicable regulatory and country-specific data protection requirements.

The subject number will be done trough an electronic case report form (eCRF) that will be created using REDCap. This system is regulatory compliant (GCP, 21CRF11, EC Clinical Trial Directive).

Source data verification

Source documents are defined as all observations or notes recorded on the clinical interventions, and all reports and notes required for assessment and reconstruction of the research study. Accordingly, source documents include but are not limited to laboratory reports, EKG tracings, ultrasonography reports, hospital reports, patient progress notes, radiologist reports, or any other reports or records of any procedure according to this protocol.

Whenever possible the original document should be kept as the source document; however, provision of a photocopy which is clear, legible and an exact duplicate of the original document and signed by the principal investigator is acceptable.

End of the trial

The end of the trial is defined as the date of the last visit of last subject undergoing the trial (LVLP).

Statement that the trial will be conducted in compliance with legal requirements

The trial will be conducted in accordance with the principles of the Declaration of Helsinki, and according to applicable regulations in Spain.

By signing the investigator's agreement, the investigator undertakes to comply with the provisions contained in applicable legislation on clinical trials and agrees to conduct the study in an efficient and diligent manner in accordance with good clinical practice guidelines, applicable European and local regulations, as well as the guidelines or regulations relating to clinical trial management.

The investigator must prepare and keep adequately study documentation according to good clinical practice, and applicable local and national regulations.

4. SUBJECT SELECTION AND WITHDRAWAL

Description of study population

Participants eligible for this study will be consecutive stable and asymptomatic HIV-infected adults (≥18 years) on BETAF once daily will be invited to participate if they have plasma HIV-1 RNA less than 50 copies/mL for at least the previous 6 months and CD4 cell counts greater than 350 cells/mL at the time of consideration for the study.

Subject inclusion criteria

Patients included into the study must meet all the following criteria:

1. stable and asymptomatic HIV-infected adults (≥18 years) on BETAF once daily for at least the previous 6 months.
2. plasma HIV-1 RNA less than 50 copies/mL for at least the previous 6 months
3. CD4 cell counts greater than 350 cells/mL at the time of consideration for the study
4. women of child-bearing potential must have a negative pregnancy test in serum before the inclusion in the study and agree to use highly effective contraceptive methods,
5. agree to participate

Subject exclusion criteria

1. prior virological failure to any antiretroviral regimen or documented resistance mutations to bictegravir, emtricitabine, or tenofovir
2. any diagnosis of a psychiatric illness
3. alcohol abuse or illicit drug consumption (based on their past medical history and specific questions at the time of recruitment)
4. patients co-infected with HIV and active hepatitis B or C virus
5. any other condition at the doctor's discretion that did not allow ensuring a correct adherence.

Withdrawal criteria

Study withdrawal

All patients have the right to withdraw their consent at any time during the study without prejudice to them.

The patient may also discontinue study participation in the following instances:

1. If the investigator considers in the interest of the subject (i.e intercurrent illness, occurrence of adverse events) that it is best for them to stop study medication.
2. The subject fails to comply with the protocol requirements or fails to cooperate with investigator.

All follow-up terminations of study patients and their reasons must be reported

immediately to monitor and sponsor and be duly documented in both the subject's CRF and source document. All patients who discontinue study medication will be asked to do the follow up and requested to attend for study visits up until week 48. If this is not possible or acceptable to the subject or Investigator, the subject may be withdrawn from the study and the reason for withdrawal recorded in the CRF. The final form in the CRF should be completed.

3. If a female subject becomes pregnant along the study, she will discontinue the study treatment.

Specific criteria for study treatment withdrawal

1. Virological failure:

   Any participant from experimental arms with confirmed (≤2 weeks) detectable HIV RNA at any follow-up visit. The patient will have genotypic resistance studied and will be re-started on full daily Biktarvy dose. The patient will be maintained into the study until completion of the 48-week follow-up.

2. Withdrawal of informed consent

3. Any reason which prevents the adequate development of the study (e.g. pregnancy, uncompensated systemic illnesses, major surgery, requirement of therapies with clinically meaningful interactions or contraindicated, etc.)

5. TREATMENT OF SUBJECTS

Description of study treatments

BIKTARVY® is a three-drug fixed dose combination product containing 50 mg of bictegravir (BIC), 200 mg of emtricitabine (FTC), and 25 mg of tenofovir alafenamide (TAF). The recommended dosage of BIKTARVY is one tablet taken orally once daily with or without food in adults for 48weeks.

Name and description of the investigational products

Treatment: BIKTARVY® is a single-tablet regimen for HIV.

Generic Name: bictegravir + tenofovir alafenamide + emtricitabine

Abbreviation: BIC + TAF + FTC

Drug Class: Single-Tablet Regimens

Company: Gilead Sciences

Approval Status: Approved by EMA and FDA

Farmaceutical form:

Each tablet contains 50 mg bictegravir, 25 mg tenofovir alafenamide, and 200 mg emtricitabine.

Dosage:

One tablet once day.

Storage conditions:

Do not store below 30 °C. Do not freeze. Summary of known and potential risks and benefits

Undesirable effects:

The most frequently reported adverse reactions were headache (5%), diarrhoea (5%) and nausea (4%).

Others common adverse reactions (≥1/100 to <1/10) are: depression, abnormal dreams, hiperbilirubinaemia and fatigue.

Contraindicated:

Hipersensitivity to the active substances or to any of the excipients

Co-administration with rifampicin and St. John's wort

Special warnings and precautions for use:

- Patients co-infected with HIV and hepatitis B or C virus

- Patients with pre-existing liver dysfunction,
- An increase in weight and in levels of blood lipids and glucose may occur during antiretroviral therapy.
- Mitochondrial dysfunction following exposure in utero.
- Osteonecrosis.
- Interactions with other medicinal products.

Labeling of investigational products:

Ther are complet IMPD In the Investigator file to consult

Bictarvy® will be distributed to Hospital Clínic Pharmacy Service labeled according to the current legislation by Pharmacy Service (Hospital clinic).

Concomitant, nonpermitted and permitted medication

<u>Non permitted and permitted medication</u>

The study medication should not be administered with rifampicin and Hypericum perforatum.

It is not recommended (case by case evaluation) concomitant administration with rifabutine, rifapentin, azithromycin, clarithromycin, carbamacepine, oxcarbacepin, fenobarbital, fenitoin, sucralfate, cyclosporine, simultaneously with magnesium, aluminium and ferrum supplementation (2 hours difference or with aliments)

Monitoring of compliance

Bictarvy® will be administered by hospital staff (Pharmacy Service of Hospital Clínic of Barcelona). Randomization, and treatment allocation.

All eligible subjects will be treated with Bictarvy®and will be assigned in a 1:1:1:1 ratio to the study treatment arms. The allocation list will be generated by an independent statistician and the randomization process and subject code assignation will be carried out electronically through the electronic case report form (eCRF) that will be created using RedCap (hosted at Hospital Clinic).

Continuation of treatment after the end of the trial

At week 48, the study will be stopped and the investigator will decide the best management and therapy for each patient.

6. VISIT SCHEDULE AND STUDY PROCEDURES

Assessment at each visit

Table 1 shows the summary of visits and procedures that will take place during the study.

1. Screening visit (from day -14 to day 0). After providing informed consent, all subjects will undergo screening assessments:

- Review inclusion and exclusion criteria.
- Obtain written informed consent.
- Medical history and physical examination (including vital signs).
- Register comorbidities.
- Register concomitant medications (including LVP).
- Blood tests for:
    - Blood cells: Red cell count, Hemoglobin, Hematocrit, Platelets, White cell count, Lymphocytes, Neutrophils
    - Biochemical general profile (including total, LDL, and HDL Cholesterol, triglycerides, Creatinine levels, glomerular filtration rate)
    - Liver tests: ALT, AST
    - Blood cells
    - Standard HIV Viral load
    - CD4 and CD8 cells
- Pregnancy test in urine will be performed in all fertile women.
- Recommendations on contraception methods during participation in the clinical trial will be provided.

Total required amount of blood at screening visit: (15 mL). Total required amount of urine at screening visit (applies only to women with childbearing potential): 6mL.

2. Baseline visit (day 0) : The following procedures will be completed prior to treatment administration:

- Randomization
- Complete physical examination.
- Register of comorbidities.
- Register concomitant medications (including LVP).
- Date administration treatment
- PSQI questionnaire and EQ-5D-5L questionnaire.
- Dual-X-absorptiometry (DXA)
- Blood tests: (only if it was not done at the screening visit)
    - Blood cells
    - Biochemical general profile (including total, LDL, and HDL Cholesterol, triglycerides, Creatinine levels, glomerular filtration rate
    - Liver tests: ALT, AST,
    - Standard and ultrasensitive plasma HIV Viral load
    - CD4 and CD8 cells, and inflammation and immune markers
    - Standart and ultrasensitive plasma viral load

   - HIV-1 reservoir in peripheral blood mononuclear cells
- Plasma and cells for pharmacokinetic studies:
   - Plasma levels of bictegravir, emtricitabine and tenofovir
   - Intracellular levels of bictegravir and phosphorylated emtricitabine and tenofovir
- Pregnancy test in urine or blood will be performed in all fertile women.
- Recommendations on contraception methods during participation in the clinical trial will be provided.
- Incidental adverse events (all visits) (overall and AE leading to discontinuation)

Total required amount of blood at baseline visit (30mL). Total required amount of urine at screening visit (applies only to women with childbearing potential): 6mL.

3. <u>Study follow-up visits</u>: week 12
   - Complete physical examination.
   - Register of comorbidities.
   - Register concomitant medications (including LVP).
   - Date administration treatment
   - PSQI questionnaire and EQ-5D-5L questionnaire.
   - Blood tests: (only if it was not done at the screening visit)
      - Blood cells
      - Biochemical general profile (including total, LDL, and HDL Cholesterol, triglycerides, Creatinine levels, glomerular filtration rate
      - Liver tests: ALT, AST,
      - Standard and ultrasensitive plasma HIV Viral load
      - CD4 and CD8 cells, and inflammation and immune markers
      - Standart and ultrasensitive plasma viral load
      - HIV-1 reservoir in peripheral blood mononuclear cells
   - Plasma and cells for pharmacokinetic studies:
      - Plasma levels of bictegravir, emtricitabine and tenofovir
      - Intracellular levels of bictegravir and phosphorylated emtricitabine and tenofovir
   - Pregnancy test in urine or blood will be performed in all fertile women.
   - Recommendations on contraception methods during participation in the clinical trial will be provided.
   - Incidental adverse events (all visits) (overall and AE leading to discontinuation)

   Total required amount of blood at baseline visit: 30mL. Total required amount of urine at screening visit (applies only to women with childbearing potential): 6mL.

4. <u>Study folow-up visits</u>: 4, 24, and 36.
   - Complete physical examination.
   - Register of comorbidities.
   - Register concomitant medications.

- Date administration treatment
- PSQI questionnaire and EQ-5D-5L questionnaire.
- Blood tests:
  - Blood cells
  - Biochemical general profile (including Total, LDL, and HDL Cholesterol, triglycerides, Creatinine levels, glomerular filtration rate
  - Liver tests: ALT, AST
  - Standard HIV Viral load
  - CD4 and CD8 cells

- Pregnancy test in urine or blood will be performed in all fertile women.
- Recommendations on contraception methods during participation in the clinical trial will be provided.
- Incidental adverse events (all visits) (overall and AE leading to discontinuation)

Total required amount of blood at baseline visit: 15mL. Total required amount of urine at screening visit (applies only to women with childbearing potential): 6mL.

5. <u>Week 48 or early termination visit.</u>

<u>Early termination visit</u>: in case of early termination of a participant, a termination visit should be done. The evaluations and procedures will be the same as the study follow up visits baseline and visit week 48.

- Complete physical examination.
- Register of comorbidities.
- Register concomitant medications.
- Date administration treatment
- PSQI questionnaire and EQ-5D-5L questionnaire.
- Dual-X-absorptiometry (DXA)
- Blood tests:
  - Blood tests
  - Biochemical general profile (including Total, LDL, and HDL Cholesterol, triglycerides, Creatinine levels, glomerular filtration rate
  - Liver tests: ALT, AST
  - Standard and ultrasensitive plasma HIV Viral load
  - CD4 and CD8 cells, and inflammation and immune markers
  - HIV-1 reservoir in peripheral blood mononuclear cells
- Plasma and cells for pharmacokinetic studies:
  - Plasma levels of bictegravir, emtricitabine and tenofovir
  - Intracellular levels of bictegravir and phosphorylated emtricitabine and tenofovir
- Pregnancy test in urine or blood will be performed in all fertile women.
- Recommendations on contraception methods during participation in the clinical trial will be provided.
- Incidental adverse events (all visits) (overall and AE leading to discontinuation)

Total required amount of blood at week 48 visit: 30mL. Total required amount of urine at screening visit (applies only to women with childbearing potential): 6mL.

	**Screening visit (from day -7 to day -1)**	**Day 0 (treatment initiation)**	**Week 12**	**Week 4, 24 , 36**	**Week 48 or Premature end**
Compliance with inclusion and exclusion criteria	X				
Randomization		X			
Clinical evaluation	X	X	X	X	X
Blood cells, biochemistry profile and liver tests(*)	X	X	X	X	X
Standard plasma HIV RNA & CD4/CD8 ratio(*)	X	X	X	X	X
Pregnancy test[1]	X[2]	X			X
Plasma levels and intracellular levels of bictegravir, emtricitabine and tenofovir sample (PK)[3]		X	X		X
Blood special storage (Virol/immunology)[3]		X	X		X
Questionnaire on adherence to ART and pill count			X	X	X
Questionnaire PSQI (α)+ Questionnaire EQ-5D-5L[3]		X	X	X	X
Dual-X-absorptiometry (DXA)		X			X
Adverse events		X	X	X	X

[1] Pregnancy test in women of childbearing age.
[2] Can be done at the screenning visit or at the baseline visit
[3] Can be done at the scrennning visit or at the baseline visit but always after signing the patient's consent

Figure 2. Schedule

Blood and urine samples for laboratory assessments during the study

Blood samples to determine hematology, biochemistry and coagulation tests will be analyzed at the local laboratory of each participating center. After analysis the samples will be destroyed locally.

The following parameters will be evaluated:

- Blood cells: Red cell count, Hemoglobin, Hematocrit, Platelets, White cell count, Lymphocytes, Neutrophils.
- Biochemistry: Glucose, AST, ALT, Creatinine, estimated glomerular filtration rate, Total, LDL, and HDL cholesterol, and triglycerides.
- HIV test
- Pregnancy test (applies exclusively to women with childbearing potential)
- CD4 (absolute and percent), CD8 (absolute and percent), Cd4/CD8 ratio standard HIV viral load

Samples for analysis at the end of the study (stored samples)

Samples for analysis at baseline visit, at 4 weeks and at 48 weeks (end of the study) will be obtained (15 mL at each visit baseline, week 4, week 48). Samples will be stored at -80ºC locally at Hospital Clinic of Barcelona.

Samples will be analyzed to determine:

- Viral reservoir (total and integrated DNA): HIV laboratory, Hospital Clínic, Barcelona
- Inflammatory markers (hsCRP, IL6, adiponectin, sCD14, sCD163): At HIV laboratory, Hospital Clínic, Barcelona
- Pharmacocinetic samples: Plasma levels and intracellular levels of bictegravir, emtricitabine and tenofovir samples (see annex 3)
- All samples will be transferred under dry ice condition and according to local and European Regulations on the transportation.

7. STATISTICAL ANALYSIS

General remarks

The statistical analysis will be carried out in accordance with the principles specified in the International Conference on Harmonization (ICH) Topic E9 (CPMP / ICH / 363/96) (55). Data analysis will be performed using the statistical software: Stata version 17.

A summary of the overall approach to statistical analysis is presented hereafter.

Also, it will stick to the recommendations given by the consensus documents of the scientific journals (i.e. CONSORT 2010 extension for pilot and feasibility trials) to improve reliability and value of medical research literature by promoting transparent and accurate reporting of clinical research studies.

Sample size

Sample size has been established at 40 patients, ten per study arm. The rationale for this number is feasibility and economic reasons. All efforts will be done to maintain follow-up of every participant during the whole study period.

Analysis populations

The population considered for this study will be:

1. Intention to Treat (ITT) population: all patients who are included into the study regardless of whether they have completed the treatment and/or protocol procedures as prescribed. This population will be used for all efficacy analyses, and all analyses of demographic and baseline characteristics.

2. On Treatment or Per Protocol Population (PP): Per protocol patient sets will be defined as those patients included in the study who have received the investigational product without major protocol deviations that might impact the study's main assessments. These deviations will be assessed during the data review prior to database lock. This population will be used to assess the immunological outcomes, viral reservoir, inflammatory and immunological markers, plasma levels and intracellular levels of bictegravir, emtricitabine and tenofovir sample levels, EQ-5D-5L and PSQI questionnaires.

3. The Safety population is defined as all included subjects who received at least one dose of the investigational product. In this study the Safety population will have the same definition than the ITT subset and thus, all safety analysis will be conducted on the ITT population.

The precise reasons for excluding subjects from the study population will be fully defined and documented before data lock.

Statistical Methods

Summary statistics will be performed using absolute frequency and percentages for qualitative variables and mean and SD or median and IQR for quantitative characteristics, at each visit. The primary and the secondary virological outcomes will be reported as absolute frequency and percentage along with the exact 95%CI, for each trial arm and visit. Laboratory parameters, weight, BMI and dexa parameters will be described with median and IQR for each trial arm and visit. Items of PSQI and EQ-5D-5L questionnaires will be reported with absolute frequency and percentage. Incidence of AEs will be reported as number of events per 100person-month.

Under descriptive/exploratory purposes, the virological outcomes and qualitative characteristics included in the secondary outcomes will be compared between groups using the Fisher's exact test. Laboratory parameters and dexa will be compared between study arms and follow-up visits by means the mixed-effects regression model. They will be log-transformed in case of non-normal residual distribution and a description with geometric mean and 95%CI will be reported too. Incidence rate of AEs will be compared between arms using the Poisson (or negative binomial) regression model. All tests will be two-tailes with a significance level set at 0.05.

Multiplicity adjustment and interim analysis

No inferential analyses are planned in the protocol. No formal imputations will be performed, the analyses will be based on the Available Data Only (ADO) approach.

Data Review (DR)

Data Review (DR) will be performed before lock of database. Data will be examined for compliance with the trial protocol by the monitor and the data manager. Deviations will be sent to the project statistician to plan listings for the Data Review. The objective is to carry out the population selection and definition of the final study populations as well as a preliminary assessment of the quality of the trial data.

8. SAFETY ASSESSMENT

Definitions

Adverse event (AE): an AE is any untoward medical occurrence in a clinical study subject administered a medicinal (investigational or non-investigational) product. An AE does not necessarily have a causal relationship with the treatment. An AE can therefore be any unfavorable and unintended sign (including an abnormal finding), symptom, or disease temporally associated with the use of a medicinal (investigational or non-investigational) product, whether or not related to that medicinal (investigational or non-investigational) product (Definition per International Conference on Harmonization [ICH]).

This includes any occurrence that is new in onset or aggravated in severity or frequency from the baseline condition, or abnormal results of diagnostic procedures, including laboratory test abnormalities.

Serious adverse event (SAE): an SAE is any untoward medical occurrence that at any dose: Results in death, is life-threatening, requires inpatient hospitalization or prolongation of existing hospitalization, results in persistent or significant disability/incapacity, is a congenital anomaly/birth defect, is a suspected transmission of any infectious agent via a medicinal product, is medically important (according to the treating physician), other important medical events that may not be immediately life-threatening or result in death or hospitalization but may jeopardize the subject or may require intervention to prevent one of the other outcomes listed in the definition above.

If a serious and unexpected AE occurs for which there is evidence suggesting a causal relationship between the study drug and the event (eg, death from anaphylaxis), the event must be reported as a serious and unexpected suspected adverse reaction even if it is a component of the study endpoint (eg, all-cause mortality).

Any event requiring hospitalization (or prolongation of hospitalization) that occurs during the course of a subject's participation in a study must be reported as an SAE, except hospitalizations for the following: hospitalizations not intended to treat an acute illness or AE (eg, social reasons such as pending placement in long-term care facility), surgery or procedure planned before entry into the study.

Adverse reaction: An AR is an AE suspected to be causally related to a medicinal product.

An ADR is a response to a medicinal product which is noxious and unintended. Response in this context means that a causal relationship between a medicinal product and an adverse event is at least a reasonable possibility (i.e. the relationship cannot be ruled out). Adverse reactions may arise from use of the product within or outside the terms of the marketing authorization or from occupational exposure. Conditions of use outside the marketing authorization include overdose, misuse, abuse and medication errors. [Sources EMA GVP Annex I, and ICH E2A].

Unexpected Adverse Reaction (UAR): Any adverse reaction, whose nature or severity of which is not consistent with the applicable product information (e.g. investigator's brochure for an unauthorized investigational product or summary of product characteristics for an authorized product).

Suspected Unexpected Serious Adverse Reaction (SUSAR): An adverse reaction that is both serious and unexpected.

Assessment of intensity

An assessment of intensity grade will be made using the general categorical descriptors outlined in the WHO Toxicity Grading Scale. The investigator should use clinical judgment in assessing the severity of events not directly experienced by the subject (eg, laboratory abnormalities).

Assessment of causality

- Possible: An AE that might be due to the use of the drug. An alternative explanation, eg, concomitant drug(s), concomitant disease(s), is inconclusive. The relationship in time is reasonable; therefore, the causal relationship cannot be excluded.

- Probable: An AE that might be due to the use of the drug. The relationship in time is suggestive (eg, confirmed by dechallenge). An alternative explanation is less likely, eg, concomitant drug(s), concomitant disease(s).

- Very Likely: An AE that is listed as a possible adverse reaction and cannot be reasonably explained by an alternative explanation, eg, concomitant drug(s), concomitant disease(s). The relationship in time is very suggestive (eg, it is confirmed by dechallenge and rechallenge).

- AE related: The relationship in time of the AE with the study drug indicates a possible causal relationship and it cannot be explained by factors such as the patient's clinical condition or therapeutic interventions.

- AE unrelated: The relationship in time of the AE with the study drug indicates an unlikely causal relationship, or other factors (concomitant medication or conditions) or other therapeutic interventions provide a satisfactory explanation for the AE.

Collection and follow up of adverse events

AEs may be recorded at each visit based on careful clinical observation of the patient, laboratory tests or spontaneously reports by the participant discovered as a result of general questioning by the study staff. All AEs will be recorded on the medical history and in the CRF. The investigator will also decide whether the adverse event is, based on his/her judgment, related or not to the study drug—this decision should also be noted in the medical history and

CRF.

The following will be recorded for each event: description, severity (grade 1, 2, 3, 4 and 5), duration (start and end dates), intensity, causal relationship with the drug (according to the previously attributability criteria) and study drug(s) for which this causal relationship is suspected, actions taken and outcome, using choices given on the patient's medical history. The investigator should report the underlying condition when a surgical or medical procedure is required as the event term, and the procedure as an action taken. For a preexisting AE that has worsened in terms of severity or frequency, the meaning of the change should be specified.

For all AEs, the Investigator must pursue and obtain information adequate both to determine the outcome of the AE and to assess whether it meets criteria for classification as a serious adverse event (SAE) requiring immediate notification (see section 8.3). Follow-up of the AE is required if the AE persists until the event resolves or stabilizes at a level acceptable to the Investigator.

The degree of severity of an adverse event provides a qualitative assessment of the extent or intensity of an adverse event elicited by the investigator or reported by the patient. Severity does not reflect the clinical seriousness of the event, only the grade or extent of the complaint or incidence.

Adverse events (including pre-treatment adverse events) must be recorded on an Adverse Event Report Form. The investigator must provide information on the adverse event, preferably with a diagnosis, or at least with signs and symptoms; start and stop dates (and start and stop time if the adverse event lasts less than 24 hours); intensity; causal relationship to IMP; action taken; and outcome. If the adverse event is an overdose, the nature of the overdose must be stated (for example, medication error, accidental overdose, or intentional overdose) and the investigator shall notify sponsor or whoever assumes the tasks delegated by the sponsor, within 24 hours from the time of knowing about the event. The reporting circuit and form will be the same as for the SAE.

In order to avoid vague, ambiguous, or colloquial expressions, all AEs should be recorded in standard medical terminology using MedDRA dictionary, rather than the patient's own words. The SAEs that have not resolved by the end of the study, or that have not resolved upon discontinuation of the subject's participation in the study, must be followed until any of the following occurs: the event resolves, the event stabilizes, the event returns to baseline, if a baseline value/status is available, the event can be attributed to agents other than the study drug or to factors unrelated to study conduct, it becomes unlikely that any additional information can be obtained (subject or health care practitioner refusal to provide additional information, lost to follow-up after demonstration of due diligence with follow-up efforts).

Information on adverse events that are not serious or unexpected and on those considered unrelated to the study treatment will be collected in tabular form at the end of the clinical trial or

at the time of interim analyses when these are planned.

The sponsor will keep a record of all AEs reported by investigators. These records will be submitted to the competent authorities when requested.

Pregnancy

Subjects will be instructed to notify the investigator if the subject or partner becomes pregnant.

If any pregnancy occurs during the course of the study, the investigator shall notify the sponsor or whoever assumes the tasks delegated by the sponsor within 24 hours of knowing about the event. Also, a follow-up of the pregnancy will be performed to document its outcome and the state of health of the newborn. If the pregnancy outcome meets the SAE criteria or if the newborn presents a serious event, the procedures for reporting an SAE will be followed.

The report shall be made using the specific form for the notification of pregnancy, which must be sent by fax or email to the same contact who receives the SAE notifications.

Procedure for expedited reporting of serious advers events and serious unexpected adverse reactions

Phamacovigilance activities will be delegated by the sponsor to Pharmacovigilance center (PV center) located at Hospital Clínic Barcelona CTU (Clinical Trial Unit), Barcelona, Spain.

Serious Adverse Events (SAEs)

The principal investigator or delegates will report immediately any SAE occurring during the study to the PV center regardless of their degree of causal relationship with the study drug. Any such serious adverse event due to any cause, whether or not related to the study medication, occurring from signing of informed consent and up to 30 days after receiving initiating the study, must be reported within 24 hours of occurrence or when the investigator becomes aware of the event.

The initial report of SAE should be written and as complete as possible including details of the current disease and SAE and assessment of the causal relationship between the AE and the investigational product. Reporting will be made using the Serious Adverse Event Report Form included in Appendix 3 of this protocol within 24 hours from first knowledge by the investigator, completing all information on the form in the following two days.

The information missing at the time of the initial report must be reported in the SAE follow-up form.

SAEs forms should be sent immediately by fax or email to:
CTU (clinical Trial Unit)
Farmacologia Clínica
Hospital Clínic Barcelona

Fax number: 93 2279877
Email address: acruceta@clinic.cat and copy to cencinas@clinic.cat ayelamosm@clinic.cat

For SAEs, the investigator will provide the PV center with all documentation related to the event (additional laboratory tests, discharge reports, etc.).

The investigator must also follow up SAEs and similarly report information related to the event until it has subsided, returned to baseline, can be attributed to products other than the study medication or to factors unrelated to conduct of the study, it is unlikely to obtain additional information, or in case of permanent impairment, until the condition stabilizes.

In the event of fatal or life-threatening, the investigator should be required to provide with all additional information requested by the study coordinator, the EC and regulatory authorities to PV center.

The PV center shall keep detailed records of all the SAEs or the events of special interest which are notified by the investigators.

In the case of a medication error or if the investigational medicinal product is used outside the provisions of the protocol, while conducting the study, the investigator shall notify the PV center within 24 hours from the time of knowing about the event. The reporting circuit and form will be the same as for the SAE.

In the case of documenting medication errors or overdose, the sponsor will carry out the necessary actions for its management and prevention (specify when necessary).

Suspected Unexpected Serious Adverse Reactions (SUSARs)

The PV center will report any events that are serious and unexpected that may be related to the investigational products to the competent authorities in accordance with local regulations.

Reporting will be made using the SAE form

The maximum deadline for reporting will be 15 calendar days from the time the sponsor is aware of the SUSAR. For SUSARs causing death or that are life-threatening for the subject, the maximum reporting time will be 7 calendar days from the time the sponsor is aware of them. This information will be completed, when possible, in the following 8 days.

Expedited reporting of other relevant safety information:

The FV center shall notify regulatory authorities, as soon as possible and no later than 15 days after having knowledge of it, any information that could alter the benefit/risk relationship of the investigational medicinal product (e.g. an increase in the rate of occurrence of the expected SAR, SUSARs that occur after the completion of a clinical trial, new events related to the conduct of the trial or the development of the investigational medicinal product, etc.).

Annual safety report

During the course of the study, the FV center will prepare periodic safety reports annually following the recommendations outlined in the ICH E2F guidelines, and they must be submitted to the regulatory authorities and the ECs involved following the timetable established in the legislation.

Opening of the blind and unmasking

The blind opening procedure is in the investigator's file. The masking list is kept in the Pharmacy Service of the Hospital Clinic of Barcelona.

Report to the investigators

The sponsor shall present the safety information that could affect the safety of the patients included in the study to the investigators as soon as possible.

In addition, the investigator will be informed throughout the study about all safety aspects, including amendments to the protocol due to safety reason.

9. DATA HANDLING

Recording of data

Data generated and/or collected will be registered in participating centers using an electronic data management system with remote data entry. Completeness and plausibility checks will ensure the collection of high quality data. An electronic case report form (eCRF) will be designed, validated and implemented with electronic data capture tools hosted at hospital ClinicREDCap and will provide electronic data capture functionality (EDC) to the investigators.

The system complies with the relevant international standards and provides the capability to perform all major data management activities within a consistent, auditable and integrated electronic environment (query management, data entry, data validation, report generation).

An e-CRF for each patient will be completed by authorized personnel.

Each centre will have access to their patients, and access will be secured by the use of unique personal logins. Each data recording or editing event will be logged in the database to allow better monitoring and database coordination.

The Sponsor or his delegates must ensure that data are recorded in the eCRF correctly and completely by authorized personnel. The investigator has to confirm the integrity of the data transferred to the eCRF by signature.

Direct access to source data/documents

Investigators will ensure access to the source documents of the staff responsible for guaranteeing data quality and data analysis. In addition, access to documentation will be provided, if necessary, to the staff duly authorized by the sponsor (study monitors), to regulatory authorities and to Ethics Committee if they request to inspect the study.

Source documents will be stored in the Investigators´s File. These documents will be kept for a minimum period of 25 years by the PI, after which they will be destroyed.

Data management

The Investigator must ensure the accuracy, completeness, legibility and timelines of data reported in the CRF and all required reports. Any change or correction to the CRF must be dates, initialled and explained (if necessary).

All documentation derived from this study must be stored for 25 years.

Archiving and storage of data

The investigator is responsible for maintaining all records which enable the conduct of the clinical trial at the site to be fully documented, in compliance with ICH GCP filing standard. Timeliness and completeness of the documentation will be regularly checked by the clinical

monitor. The documentation of the clinical trial including all the relevant correspondence should be kept by the investigator for the minimum period required by applicable regulatory and country-specific requirements.
All completed study related documents (e.g. eCRF, Informed consent forms, drug accountability logs, staff signature lists, Subject identification log, …) must be archived by 25 years.

Audit and inspection

The investigators agree to comply with the requirements of the sponsor and the relevant authority for an audit or an inspection of the study.

The audit can apply to all stages of the study, from development of the protocol to publication of the results and filing the data used or produced in the study.

10.QUALITY CONTROL AND QUALITY ASSURANCE

According to ICH/GCP guidelines, the sponsor should ensure that the trial is adequately monitored.

The purpose of monitoring is to verify that the rights and wellbeing of human subjects are protected; that the trial is accurate, complete and verifiable with source data and that the trial is conducted in compliance with the protocol, GCP and the applicable regulatory requirements.
To ensure homogeneity and the same quality standards, monitors in all study countries will be trained with the same procedures.

Source data will be verified during on-site monitoring visits. During the visits, the monitor will compare the data entered into the eCRF with the source documents. The nature and location of all source documents will be identified to ensure that all sources of original data required to complete the eCRF are known to the monitor and study-site personnel and are accessible for verification. During monitoring visits, the relevant study-site personnel should be available, the source documentation accessible, and a suitable environment provided for review of suty-related documents.

A close-out visits will be performed before the final closure of each centre. During this visit, medication accountability will be checked and arranged according to the indications of the final version of the protocol. Besides, a general overview and a final revision of pending queries will be implemented, ensuring that all data and corresponding source documents are in place before the site closure.

11.ETHICAL AND REGULATORY CONSIDERATIONS

Ethics approval

This study will be conducted in accordance with the protocol and ethical principles stated in the Declaration of Helsinki or the applicable guidelines on GCP, and all applicable local laws, rules, and regulations.

Requirements for ethical review as set forth in Directive 2001/20/EC of the European Parliament and of the Council of 4 April 2001 on the approximation of the laws, regulations and administrative provisions of the Member States relating to the implementation of good clinical practice (GCP) in the conduct of clinical trials on medicinal products for human use or other relevant local regulations for institutional review will be followed. The Protocol, informed consent form, Investigator's Brochure and other required documents must be approved by the Ethics Committee before enrolment of subjects in the study. The letter of approval from the Ethics Committee, as well as a list of documents reviewed, will be filed in the Investigator Site File (ISF) and a copy will be filed in the trial master file (TMF) held by the Sponsor.

The Sponsor and his delegates, in collaboration with the investigator, will be responsible for reporting to the Ethics Committee all changes in research activity, including protocol amendments, updates of Investigator's Brochures, annual safety reports, all unanticipated problems involving risks to human subjects, and study termination.

Regulatory considerations

In parallel to the submission to the Ethics Committee, the Sponsor has to obtain an authorisation from the appropriate competent authority to conduct the clinical study. Subjects must not be entered into the study until the relevant Ethics Committee has issued its opinion and the CA has given authorisation to conduct the study.

All substantial amendments must be submitted to the Ethics Committee and/or to the Competent authorities for approval.

Neither the investigator nor the sponsor will modify this protocol without a formal amendment by the sponsor. All protocol amendments must be issued by the sponsor, and signed and dated by the investigator. Protocol amendments must not be implemented without prior Ethics Committee approval, or when the relevant competent authority has raised any grounds for non-acceptance, except when necessary to eliminate immediate hazards to the subjects, in which case the amendment must be promptly submitted to the Ethics Committee and relevant competent authority.

Documentation of amendment approval by the investigator and Ethics Committee must be provided to the sponsor. During the course of the study, in situations where a departure from the protocol is unavoidable, the investigator or other physician in attendance will contact the appropriate sponsor representative. Except in emergency situations, this contact should be made before implementing any departure from the protocol. In all cases, contact with the sponsor must be made as soon as possible to discuss the situation and agree on an appropriate

course of action. The data recorded in the eCRF and source documents will reflect any departure from the protocol, and the source documents will describe this departure and the circumstances requiring it.

Responsibilities of the investigators

The investigator is responsible for ensuring that the study is performed in accordance with the protocol, current ICH guidelines on Good Clinical Practice (GCP), and applicable regulatory and country-specific requirements. Good Clinical Practice is an international ethical and scientific quality standard for designing, conducting, recording, and reporting studies that involve the participation of human subjects. Compliance with this standard provides public assurance that the rights, safety, and well-being of study subjects are protected, consistent with the principles that originated in the Declaration of Helsinki (and updated according to its last version, Fortaleza, Brazil, 2013), and that the study data are credible.

In addition, the investigator is responsible for giving information about the study to all staff members involved in the study or in any element of subject management, before and during the course of the study.

The investigator is also responsible for ensuring the privacy, health, and welfare of the subjects during and after the study. The investigator(s) must be familiar with the background and requirements of the study and with the properties of the investigational product as described in the Investigator's Brochure.

Before the start of the study, the investigator (or sponsor where required) will provide the Ethics Committee with current and complete copies of the following documents (as required by local regulations).

Informed consent

Each subject must give written consent according to local requirements after the nature of the study has been fully explained. The ICF(s) must be signed before performance of any study related activity. The ICF(s) that is/are used must be approved by both the sponsor and by the reviewing Ethics Committee and be in a language that the patient can read and understand.

The informed consent should be in accordance with principles that originated in the eclaration of Helsinki (updated according to its last version, Fortaleza, Brazil, 2013), current ICH and GCP guidelines, applicable regulatory and country-specific requirements, and sponsor policy. Before enrollment in the study, the investigator or an authorized member of the study- site personnel must explain to potential subjects the aims, methods, reasonably anticipated benefits, and potential hazards of the study, and any discomfort that the participation in the study may entail.

Subjects will be informed that their participation is voluntary and that they may withdraw consent to participate at any time. They will be informed that choosing not to participate will not affect the care the subject will receive for the treatment of his or her disease. Subjects will be told that alternative treatments are available if they refuse to take part and that such

refusal will not prejudice future treatment. Finally, they will be told that the investigator will maintain a subject identification register and that their records may be accessed by health authorities and authorized sponsor personnel without violating the confidentiality of the subject, to the extent permitted by the applicable law(s) or regulations. By signing the ICF the subject is authorizing such access, including permission to obtain information about his or her survival status, and agrees to allow his or her study physician to recontact the subject for the purpose of obtaining consent for additional safety evaluations, if needed, and subsequent disease-related treatments, or to obtain information about his or her survival status.

The subject will be given sufficient time to read the ICF and the opportunity to ask questions.

After this explanation and before entry into the study, consent should be appropriately recorded by means of the subject's personally dated signature. After having obtained the consent, a copy of the ICF must be given to the subject.

Patient confidentiality

The collection and processing of personal data from subjects enrolled in this study will be limited to those data that are necessary to fulfill the objectives of the study. Patient data will be anonymized to ensure confidentiality and compliance with applicable data privacy protection laws and regulations.

Subjects will be codified with a study code that prevents their identity from being deduced.

The PI and duly authorized collaborators will compromise to maintain personal data strictly confidential, according to the corresponding country-specific requirements. The link between the numeric code and real personal data from subjects will be rigorously kept by the PI. The informed consent obtained from the subject includes explicit consent for the processing of personal data and for the investigator/institution to allow direct access to his or her original medical records (source data/documents) for study-related monitoring, audit, Ethics Committee review, and regulatory inspection.

In the case report form, the patient will only be identified by the assigned study code. The name of patients will not appear in any publication or report of the study results.

The participation of the patient in the trial will be noted in their medical records.

The investigator will complete a list which will include the names of the patients participating in the trial, the number of inclusion in the study, and their medical history. Only investigators and the staff responsible for guaranteeing data quality and data analysis will have access to the clinical documentation of the participants.

Duly authorized persons by the sponsor and the health authorities and the Ethics Committee may audit or inspect the trial. Personal information will not be publicly available, in compliance with Personal Data Protection regulation.

The subject has the right to request through the investigator access to his or her personal data and the right to request rectification of any data that are not correct or complete pursuant to European requirements (EU Directive 2016/679, on data protection). According to the above law, patients can exercise their rights to data access, rectification, opposition, cancellation, request a copy and portability, for which they must contact the study doctor.

In addition, the sponsor cannot make decisions as to the significance of any findings resulting from exploratory research. Therefore, exploratory research data will not be returned to subjects or investigators, unless required by law or local regulations. Privacy and confidentiality of data generated in the future on stored samples will be protected by the same standards applicable to all other clinical data.

Only data collected for the study that does not bear any information that could directly identify the patient will be transferred to third parties or other countries. Should this transfer occur, it will be for the same purposes as the study and guarantee confidentiality with at least the level of protection afforded by applicable regulations in Spain.

Patients will be informed that their clinical data will be incorporated into an automated study-specific file after and the results of the clinical trials and different studies conducted with samples can be communicated at scientific meetings, medical conferences or publications. However, patient's identity or identifiable data will never be disclosed.

12.FINANCING AND INSURANCE

Financing

This study is funded in part by a grant received from Instituto de Salud Carlos III (PI20/869)

Insurance

The Sponsor or its **delegate** will procure insurance for this clinical trial to cover trial related injuries of the participants according to local regulatory requirements.

Clinical trial participants will be provided on request with the conditions of insurance along with the patient information and consent form.

13. PUBLICATION

The main results will be published according regulatory SpanIsh requeriments.

PROTOCOL APPENDIX

APPENDIX 1. DECLARACIÓN DE HELSINKI

Appendix I. World Medical Association Declaration of Helsinki Ethical Principles for Medical Research Involving Human Subjects

World Medical Association

Adopted by the 18th WMA General Assembly, Helsinki, Finland, June 1964, and amended by the: 29th WMA General Assembly, Tokyo, Japan, October 1975

35th WMA General Assembly, Venice, Italy, October 1983 41st WMA General Assembly, Hong Kong, September 1989

48th WMA General Assembly, Somerset West, Republic of South Africa, October 1996 52nd WMA General Assembly, Edinburgh, Scotland, October 2000 53rd WMA General Assembly, Washington, DC, USA, October 2002 (Note of Clarification added) 55th WMA General Assembly, Tokyo, Japan, October 2004 (Note of Clarification added)

59th WMA General Assembly, Seoul, Republic of Korea, October 2008 64th WMA General Assembly, Fortaleza, Brazil, October 2013

Preamble

1- The World Medical Association (WMA) has developed the Declaration of Helsinki as a statement of ethical principles for medical research involving human subjects, including research on identifiable human material and data.

   The Declaration is intended to be read as a whole and each of its constituent paragraphs should be applied with consideration of all other relevant paragraphs.

2- Consistent with the mandate of the WMA, the Declaration is ad- dressed primarily to physicians. The WMA encourages others who are involved in medical research involving human subjects to adopt these principles.

General Principles

3- The Declaration of Geneva of the WMA binds the physician with the words, “The health of my patient will be my first consideration,” and the International Code of Medical Ethics declares that, “A physician shall act in the patient's best interest when providing medical care.”

4- It is the duty of the physician to promote and safeguard the health, well-being and rights of patients, including those who are involved in medical research. The physician's knowledge and conscience are dedicated to the fulfilment of this duty.

5- Medical progress is based on research that ultimately must include studies involving human subjects.

6- The primary purpose of medical research involving human subjects is to understand the causes, development and effects of diseases and improve preventive, diagnostic and

therapeutic interventions (methods, procedures and treatments). Even the best proven interventions must be evaluated continually through research for their safety, effectiveness, efficiency, accessibility and quality.

7- Medical research is subject to ethical standards that promote and ensure respect for all human subjects and protect their health and rights.

8- While the primary purpose of medical research is to generate new knowledge, this goal can never take precedence over the rights and interests of individual research subjects.

9- It is the duty of physicians who are involved in medical research to protect the life, health, dignity, integrity, right to self- determination, privacy, and confidentiality of personal information of research subjects. The responsibility for the protection of research subjects must always rest with the physician or other health care professionals and never with the research subjects, even though they have given consent.

10- Physicians must consider the ethical, legal and regulatory norms and standards for research involving human subjects in their own countries as well as applicable international norms and standards. No national or international ethical, legal or regulatory requirement should reduce or eliminate any of the protections for research subjects set forth in this Declaration.

11- Medical research should be conducted in a manner that minimises possible harm to the environment.

12- Medical research involving human subjects must be conducted only by individuals with the appropriate ethics and scientific education, training and qualifications. Research on patients or healthy volunteers requires the supervision of a competent and appropriately qualified physician or other health care professional.

13- Groups that are underrepresented in medical research should be provided appropriate access to participation in research.

14- Physicians who combine medical research with medical care should involve their patients in research only to the extent that this is justified by its potential preventive, diagnostic or therapeutic value and if the physician has good reason to believe that participation in the research study will not adversely affect the health of the patients who serve as research subjects.

15- Appropriate compensation and treatment for subjects who are harmed as a result of participating in research must be ensured.

Risks, Burdens and Benefits

16- In medical practice and in medical research, most interventions involve risks and burdens.

Medical research involving human subjects may only be con- ducted if the importance of the objective outweighs the risks and burdens to the research subjects.

17- All medical research involving human subjects must be pre- ceded by careful assessment of predictable risks and burdens to the individuals and groups involved in the research in comparison with foreseeable benefits to them and to other individuals or groups affected by the condition under investigation.

Measures to minimise the risks must be implemented. The risks must be continuously monitored, assessed and documented by the researcher.

1- Physicians may not be involved in a research study involving human subjects unless they are confident that the risks have been adequately assessed and can be satisfactorily managed.

When the risks are found to outweigh the potential benefits or when there is conclusive proof of definitive outcomes, physicians must assess whether to continue, modify or immediately stop the study.

Vulnerable Groups and Individuals

2- Some groups and individuals are particularly vulnerable and may have an increased likelihood of being wronged or of incurring additional harm.

   All vulnerable groups and individuals should receive specifically considered protection.

3- Medical research with a vulnerable group is only justified if the research is responsive to the health needs or priorities of this group and the research cannot be carried out in a non- vulnerable group. In addition, this group should stand to benefit from the knowledge, practices or interventions that result from the research.

Scientific Requirements and Research Protocols

4- Medical research involving human subjects must conform to generally accepted scientific principles, be based on a thorough knowledge of the scientific literature, other relevant sources of information, and adequate laboratory and, as appropriate, ani- mal experimentation. The welfare of animals used for research must be respected.

5- The design and performance of each research study involving human subjects must be clearly described and justified in a research protocol.

The protocol should contain a statement of the ethical considerations involved and should indicate how the principles in this Declaration have been addressed. The protocol should include information regarding funding, sponsors, institutional affiliations, potential conflicts of interest, incentives for subjects and information regarding provisions for treating and/or compensating subjects who are harmed as a consequence of participation in the research study.

In clinical trials, the protocol must also describe appropriate arrangements for post-trial provisions.

Research Ethics Committees

6- The research protocol must be submitted for consideration, comment, guidance and approval to the concerned research ethics committee before the study begins. This committee must be transparent in its functioning, must be independent of the re- searcher, the sponsor and any other undue influence and must be duly qualified. It must

take into consideration the laws and regulations of the country or countries in which the research is to be performed as well as applicable international norms and standards but these must not be allowed to reduce or eliminate any of the protections for research subjects set forth in this Declaration.

The committee must have the right to monitor ongoing studies. The researcher must provide monitoring information to the committee, especially information about any serious adverse events. No amendment to the protocol may be made without consideration and approval by the committee. After the end of the study, the researchers must submit a final report to the committee containing a summary of the study's findings and conclusions.

Privacy and Confidentiality

1- Every precaution must be taken to protect the privacy of re- search subjects and the confidentiality of their personal information.

Informed Consent

2- Participation by individuals capable of giving informed consent as subjects in medical research must be voluntary. Although it may be appropriate to consult family members or community leaders, no individual capable of giving informed consent may be enrolled in a research study unless he or she freely agrees.

3- In medical research involving human subjects capable of giving informed consent, each potential subject must be adequately informed of the aims, methods, sources of funding, any possible conflicts of interest, institutional affiliations of the re- searcher, the anticipated benefits and potential risks of the study and the discomfort it may entail, post-study provisions and any other relevant aspects of the study. The potential subject must be informed of the right to refuse to participate in the study or to withdraw consent to participate at any time without reprisal. Special attention should be given to the specific information needs of individual potential subjects as well as to the methods used to deliver the information.

After ensuring that the potential subject has understood the in- formation, the physician or another appropriately qualified individual must then seek the potential subject's freely-given in- formed consent, preferably in writing. If the consent cannot be expressed in writing, the non-written consent must be formally documented and witnessed.

All medical research subjects should be given the option of being informed about the general outcome and results of the study.

4- When seeking informed consent for participation in a research study the physician must be particularly cautious if the potential subject is in a dependent relationship with the physician or may consent under duress. In such situations the informed con- sent must be sought by an appropriately qualified individual who is completely independent of this relationship.

1- For a potential research subject who is incapable of giving in- formed consent, the physician must seek informed consent from the legally authorised representative. These individuals must not be included in a research study that has no likelihood of benefit for them unless it is intended to promote the health of the group represented by the potential subject, the research cannot in- stead be performed with persons capable of providing in- formed consent, and the research entails only minimal risk and minimal burden.

2- When a potential research subject who is deemed incapable of giving informed consent is able to give assent to decisions about participation in research, the physician must seek that assent in addition to the consent of the legally authorised representative. The potential subject's dissent should be respected.

3- Research involving subjects who are physically or mentally in- capable of giving consent, for example, unconscious patients, may be done only if the physical or mental condition that prevents giving informed consent is a necessary characteristic of the research group. In such circumstances the physician must seek informed consent from the legally authorised representative. If no such representative is available and if the research cannot be delayed, the study may proceed without informed consent provided that the specific reasons for involving subjects with a condition that renders them unable to give informed consent have been stated in the research protocol and the study has been approved by a research ethics committee. Consent to remain in the research must be obtained as soon as possible from the subject or a legally authorised representative.

4- The physician must fully inform the patient which aspects of their care are related to the research. The refusal of a patient to participate in a study or the patient's decision to withdraw from the study must never adversely affect the patient-physician relationship.

5- For medical research using identifiable human material or data, such as research on material or data contained in biobanks or similar repositories, physicians must seek informed consent for its collection, storage and/or reuse. There may be exceptional situations where consent would be impossible or impracticable to obtain for such research. In such situations the re- search may be done only after consideration and approval of a research ethics committee.

Useof Placebo

6- The benefits, risks, burdens and effectiveness of a new inter- vention must be tested against those of the best proven inter- vention(s), except in the following circumstances:

Where no proven intervention exists, the use of placebo, or no intervention, is acceptable; or

Where for compelling and scientifically sound methodological reasons the use of any intervention less effective than the best proven one, the use of placebo, or no intervention is necessary to determine the efficacy or safety of an intervention

and the patients who receive any intervention less effective than the best proven one, placebo, or no intervention will not be sub- ject to additional risks of serious or irreversible harm as a result of not receiving the best proven intervention.

Extreme care must be taken to avoid abuse of this option.

Post-Trial Provisions

1- Inadvanceofaclinicaltrial, sponsors,researchersandhostcoun- try governments should make provisions for post-trial access for all participants who still need an intervention identified as ben- eficial in the trial. This information must also be disclosed to par- ticipants during the informed consent process.

Research Registration and Publication and Dissemination of Results

2- Every research study involving human subjects must be registered in a publicly accessible database before recruitment of the first subject.

3- Researchers, authors, sponsors, editors and publishers all have ethical obligations with regard to the publication and dissemination of the results of research. Researchers have a duty to make publicly available the results of their research on human subjects and are accountable for the completeness and accuracy of their reports. All parties should adhere to accepted guidelines for ethical reporting. Negative and inconclusive as well as positive results must be published or otherwise made publicly avail- able. Sources of funding, institutional affiliations and conflicts of interest must be declared in the publication. Reports of re- search not in accordance with the principles of this Declaration should not be accepted for publication.

Unproven Interventions in Clinical Practice

4- In the treatment of an individual patient, where proven inter- ventions do not exist or other known interventions have been ineffective, the physician, after seeking expert advice, with in- formed consent from the patient or a legally authorised repre- sentative, may use an unproven intervention if in the physi- cian's judgement it offers hope of saving life, re-establishing health or alleviating suffering. This intervention should subse- quently be made the object of research, designed to evaluate its safety and efficacy. In all cases, new information must be re- corded and, where appropriate, made publicly available.

APPENDIX 2. SPECIFIC SPANISH QUESTIONNARIES

Cuestionario de Salud

Versión en español para España

(Spanish version for Spain)

Spain (Spanish) © 2009 EuroQol Group EQ-5D™ is a trade mark of the EuroQol Group

Debajo de cada enunciado, marque UNA casilla, la que mejor describe su salud HOY.

MOVILIDAD

No tengo problemas para caminar ❑

Tengo problemas leves para caminar ❑

Tengo problemas moderados para caminar ❑

Tengo problemas graves para caminar ❑

No puedo caminar ❑

AUTO-CUIDADO

No tengo problemas para lavarme o vestirme ❑

Tengo problemas leves para lavarme o vestirme ❑

Tengo problemas moderados para lavarme o vestirme ❑

Tengo problemas graves para lavarme o vestirme ❑

No puedo lavarme o vestirme ❑

ACTIVIDADES COTIDIANAS *(Ej.: trabajar, estudiar, hacer las tareas domésticas, actividades familiares o actividades durante el tiempo libre)*

No tengo problemas para realizar mis actividades cotidianas ❑

Tengo problemas leves para realizar mis actividades cotidianas ❑

Tengo problemas moderados para realizar mis actividades cotidianas ❑

Tengo problemas graves para realizar mis actividades cotidianas ❑

No puedo realizar mis actividades cotidianas ❑

DOLOR / MALESTAR

No tengo dolor ni malestar ❑

Tengo dolor o malestar leve ❑

Tengo dolor o malestar moderado ❑

Tengo dolor o malestar fuerte ❑

Tengo dolor o malestar extremo ❑

ANSIEDAD / DEPRESIÓN

No estoy ansioso ni deprimido ❑

Estoy levemente ansioso o deprimido ❑

Estoy moderadamente ansioso o deprimido ❑

Estoy muy ansioso o deprimido ❑

Estoy extremadamente ansioso o deprimido ❑

Spain (Spanish) © 2009 EuroQol Group EQ-5D™ is a trade mark of the EuroQol Group

- Nos gustaría conocer lo buena o mala que es su salud HOY.
- La escala está numerada del 0 al 100.
- 100 representa la mejor salud que usted se pueda imaginar.
  0 representa la peor salud que usted se pueda imaginar.
- Marque con una X en la escala para indicar cuál es su estado de salud HOY.
- Ahora, en la casilla que encontrará a continuación escriba el número que ha marcado en la escala.

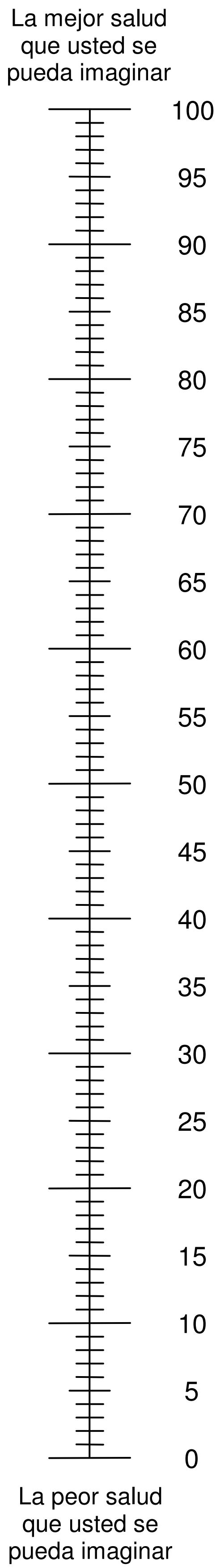

SU SALUD HOY = ☐

Spain (Spanish) © 2009 EuroQol Group EQ-5D™ is a trade mark of the EuroQol Group

ÍNDICE DE CALIDAD DE SUEÑO DE PITTSBURGH
(PSQI)

APELLIDOS Y NOMBRE:______________________ N.º HªC:__________

SEXO:__________ ESTADO CIVIL:__________ EDAD:__________ FECHA: __________

INSTRUCCIONES:

Las siguientes preguntas hacen referencia a cómo ha dormido Vd. **normalmente durante el último mes**. Intente ajustarse en sus respuestas de la manera más exacta posible a lo ocurrido durante la **mayor parte** de los días y noches del **último mes.**

¡Muy Importante! CONTESTE A TODAS LAS PREGUNTAS

1. Durante el **último mes**, ¿Cuál ha sido, normalmente, su hora de acostarse?

APUNTE SU HORA HABITUAL DE ACOSTARSE: ________

2. ¿Cuánto tiempo habrá tardado en dormirse, **normalmente**, las noches del **último mes**?

APUNTE EL TIEMPO EN MINUTOS: ________

3. Durante el **último mes**, ¿a qué hora se ha levantado **habitualmente** por la mañana?

APUNTE SU HORA HABITUAL DE LEVANTARSE: ________

4. ¿Cuántas horas calcula que habrá dormido **verdaderamente** cada noche durante el **último mes**? (El tiempo puede ser diferente al que Vd. permanezca en la cama).

APUNTE LAS HORAS QUE CREA HABER DORMIDO: ________

Para cada una de las siguientes preguntas, elija la respuesta que más se ajuste a su caso. Intente contestas a TODAS las preguntas.

5. Durante el **último mes**, cuántas veces ha tenido Vd. problemas para dormir a causa de:

a) No poder conciliar el sueño en la primera media hora:
- Ninguna vez en el último mes ____
- Menos de una vez a la semana ____
- Una o dos veces a la semana ____
- Tres o más veces a la semana ____

b) Despertarse durante la noche o de madrugada:
- Ninguna vez en el último mes ____
- Menos de una vez a la semana ____
- Una o dos veces a la semana ____
- Tres o más veces a la semana ____

c) Tener que levantarse para ir al servicio:
- Ninguna vez en el último mes ____
- Menos de una vez a la semana ____
- Una o dos veces a la semana ____
- Tres o más veces a la semana ____

d) No poder respirar bien:
- Ninguna vez en el último mes ____
- Menos de una vez a la semana ____
- Una o dos veces a la semana ____
- Tres o más veces a la semana ____

e) Toser o roncar ruidosamente:
- Ninguna vez en el último mes ____
- Menos de una vez a la semana ____
- Una o dos veces a la semana ____
- Tres o más veces a la semana ____

f) Sentir frío:
- Ninguna vez en el último mes ____
- Menos de una vez a la semana ____
- Una o dos veces a la semana ____
- Tres o más veces a la semana ____

g) Sentir demasiado calor:
- Ninguna vez en el último mes ____
- Menos de una vez a la semana ____
- Una o dos veces a la semana ____
- Tres o más veces a la semana ____

h) Tener pesadillas o «malos sueños»:
- Ninguna vez en el último mes ____
- Menos de una vez a la semana ____
- Una o dos veces a la semana ____
- Tres o más veces a la semana ____

i) Sufrir dolores:
- Ninguna vez en el último mes ______
- Menos de una vez a la semana ______
- Una o dos veces a la semana ______
- Tres o más veces a la semana ______

j) Otras razones (por favor, descríbalas a continuación):

____
____
____

- Ninguna vez en el último mes ______
- Menos de una vez a la semana ______
- Una o dos veces a la semana ______
- Tres o más veces a la semana ______

6. Durante el **último mes**, ¿cómo valoraría, en conjunto, la calidad de su sueño?
- Bastante buena ______
- Buena ______
- Mala ______
- Bastante mala ______

7. Durante el **último mes,** ¿cuántas veces habrá tomado medicinas (por su cuenta o recetadas por el médico) para dormir?
- Ninguna vez en el último mes ______
- Menos de una vez a la semana ______
- Una o dos veces a la semana ______
- Tres o más veces a la semana ______

8. Durante el **último mes**, ¿cuántas veces ha sentido somnolencia mientras conducía, comía, o desarrollaba alguna otra actividad?
- Ninguna vez en el último mes ______
- Menos de una vez a la semana ______
- Una o dos veces a la semana ______
- Tres o más veces a la semana ______

9. Durante el **último mes**, ¿ha representado para Vd. mucho problema el «tener ánimos» para realizar alguna de las actividades detalladas en la pregunta anterior?
- Ningún problema ______
- Sólo un leve problema ______
- Un problema ______
- Un grave problema ______

10. ¿Duerme Vd. solo o acompañado?
- Solo ______
- Con alguien en otra habitación ______
- En la misma habitación, pero en otra cama ______
- En la misma cama ______

POR FAVOR, SÓLO CONTESTE A LAS SIGUIENTES PREGUNTAS EN EL CASO DE QUE DUERMA ACOMPAÑADO.

Si Vd. tiene pareja o compañero de habitación, pregúntele si durante el **último mes** Vd. ha tenido:

a) Ronquidos ruidosos.
- Ninguna vez en el último mes ______
- Menos de una vez a la semana ______
- Una o dos veces a la semana ______
- Tres o más veces a la semana ______

b) Grandes pausas entre respiraciones mientras duerme.
- Ninguna vez en el último mes ______
- Menos de una vez a la semana ______
- Una o dos veces a la semana ______
- Tres o más veces a la semana ______

c) Sacudidas o espasmos de piernas mientras duerme.
- Ninguna vez en el último mes ______
- Menos de una vez a la semana ______
- Una o dos veces a la semana ______
- Tres o más veces a la semana ______

d) Episodios de desorientación o confusión mientras duerme.
- Ninguna vez en el último mes ______
- Menos de una vez a la semana ______
- Una o dos veces a la semana ______
- Tres o más veces a la semana ______

e) Otros inconvenientes mientras Vd. duerme (Por favor, descríbalos a continuación):

____
____
____
____
____

- Ninguna vez en el último mes ______
- Menos de una vez a la semana ______
- Una o dos veces a la semana ______
- Tres o más veces a la semana ______

CORRECCIÓN DEL CUESTIONARIO DE PITTSBURGH

El Índice de Calidad de sueño de Pittsburgh (PSQI) consta de 19 preguntas autoaplicada y de 5 preguntas evaluadas por la pareja del paciente o por su compañero/a de habitación (si éste está disponible). Sólo las preguntas auto-aplicadas están incluidas en el puntaje. Los 19 Items auto-evaluados se combinan entre sí para formar siete «componentes» de puntuación, cada uno de los cuales tiene un rango entre 0 y 3 puntos. En cualquier caso, una puntuación de 0 puntos indica que no existe dificultad, mientras que un puntuación de 3 indica una severa dificultad. Los siete componentes entonces se suman para rendir una puntuación global, que tiene un rango de 0 a 21 puntos, indicando una puntuación de 0 puntos la no existencia de dificultades, y una de 21 indicando severas dificultades en todas las áreas estudiadas.

Para corregir, proceda de la siguiente manera:

Componente 1: Calidad subjetiva del sueño

Examine la pregunta n.º 6, y asigne la puntuación correspondiente:

Respuesta	*Puntuación del componente 1*
«Muy buena»	0
«Bastante buena»	1
«Bastante mala»	2
"Muy mala»	3

Puntuación del componente 1:———

Componente 2: Latencia de sueño

1.º Examine la pregunta n.º 2, y asigne la puntuación correspondiente:

Respuesta	*Puntuación*
< ó = a 15'	0
16-30 minutos	1
31-60 minutos	2
> 60 minutos	3

Puntuación de la pregunta n.º 2:———

2.º Examine la pregunta n.º 5a, y asigne la puntuación correspondiente:

Respuesta	*Puntuación*
Ninguna vez en el último mes	0
Menos de una vez a la semana	1
Una o dos veces a la semana	2
Tres o más veces a la semana	3

Puntuación de la pregunta n.º 5a:———

3.º Sume las puntuaciones de las preguntas n.º 2 y n.º 5a

Suma de las *puntuaciones* de las preguntas n.º 2 y n.º 5a:———

4.º Asigne la puntuación del componente 2 como sigue:

Suma de n.º 2 y n.º 5a	*Puntuación*
0	0
1-2	1
3-4	2
5-6	3

Puntuación del componente 2:———

Componente 3: Duración del sueño

Examine la pregunta n.º 4, y asigne las puntuaciones correspondientes:

Respuesta	*Puntuación del componente 3*
> 7 horas	0
6-7 horas	1
5-6 horas	2
< 5 horas	3

Puntuación del componente 3:———

Componente 4: eficiencia de sueño habitual

1.º Escriba aquí la cantidad de horas dormidas:

2.º Calcule el número de horas permanecidas en la cama:

Hora de levantarse (pregunta n.º 3) ———
Hora de acostarse (pregunta n.º 1) ———
Número de horas permanecidas en la cama: ———

3.º Calcule la eficiencia habitual de sueño como sigue:

(Número de horas dormidas/número de horas permanecidas en la cama) x 100 = Eficiencia habitual de sueño (%)

(———/———) x 100 = ———%

4.º Asigne la puntuación del componente 4 como sigue:

Eficiencia habitual de sueño%	*Puntuación*
> 85%	0
75-84%	1
65-74%	2
< 65%	3

Puntuación del componente 4:———

Componente 5: Perturbaciones del sueño

1.º Examine las preguntas del n.º 5b al 5j, y asigne puntuaciones para cada pregunta según sigue:

Respuesta	*Puntuación*
Ninguna vez en el último mes	0
Menos de una vez a la semana	1
Una o dos veces a la semana	2
Tres o más veces a la semana	3

Puntuación n.º 5b ———
n.º 5c ———
n.º 5d ———
n.º 5e ———
n.º 5f ———
n.º 5g ———
nº 5h ———
n.º 5i ———
n.º 5j ———

2.º Sume las puntuaciones de las preguntas 5b a 5j:

Suma de 5b a 5j:———

3.º Asigne la puntuación del componente 5 como sigue:

Suma de 5b a 5j	*Puntuación del componente 5*
0	0
1-9	1
10-18	2
19-27	3

Puntuación del componente 5:———

Puntuación Global del PSQI

Sume las puntuaciones de los 7 componentes:

Componente 6: Uso se medicación hipnótica

Examine la pregunta n.º 7 y asigne la puntuación que corresponda:

Respuesta	*Puntuación*
Ninguna vez en el último mes	0
Menos de una vez a la semana	1
Una o dos veces a la semana	2
Tres o más veces a la semana	3

Puntuación del componente 6:———

Componente 7: Disfunción diurna

1.º Examine la pregunta n.º 8, y asigne las puntuaciones como sigue:

Respuesta	Puntuación
Ninguna vez en el último mes	0
Menos de una vez a la semana	1
Una o dos veces a la semana	2
Tres o más veces a la seman	3

Puntuación de la pregunta n.º 8:———

2.º Examine la pregunta n.º 9, y asigne las puntuaciones como sigue:

Respuesta	*Puntuación*
Ningún problema	0
Sólo un leve problema	1
Un problema	2
Un grave problema	3

3.º Sume las puntuaciones de las preguntas nº 8 y nº 9:

Suma de nº 8 y nº 9:———

4º Asigne las puntuaciones del componente 7 como sigue:

Suma de n.º 8 y n.º 9	*Puntuaciones*
0	0
1-2	1
3-4	2
5-6	3

Puntuación del componente 7:———

Puntuación total del PSQI:———

APPENDIX 3. PHARMACOKINETIC SAMPLES

Plasma and PBMCs samples protocols (University of Turin – CoQua Lab)

- Taking PK samples should occur as near to the specified time points as possible. However, regardless of when the blood sample was drawn, the EXACT time this happened should be recorded on the CRF form. In case of delay, omit PK sample only and leave time of sampling on the CRF blank, if next PK sample should be taken in within the next 15 mins. Discard the tube and inform laboratory of the missing tube and timepoint.

- After blood withdrawal, make sure the blood collection tubes are gently upturned 8-10 times to mix the solution in the tubes with the blood.

- All PK samples should be transferred immediately to the laboratory. The plasma PK sample should be transferred together with the 2 x 8ml CPT tubes for the PBMC sample which is collected at the defined time point. Due to the nature of the PBMC work, this sample must be processed immediately.

- Upon arrival of blood samples at the laboratory, PK-samples should be processed by the laboratory within one hour after arrival, at least.

SAMPLES:
2 mL of Lithium Heparin plasma (from n.1 7 mL LH tube). One mL for NRTIs and one mL for bictegravir
2 aliquots PBMCs (from n. 2 CPT tube). One for NRTIs and one for bictegravir

REQUIRED MATERIALS and PROTOCOLS:
- **PLASMA**: in a 7 mL lithium-heparin tube which must be centrifuged at 1400 x g (about 3000 rpm) for 10 minutes at 4° C to obtain two aliquots of about 1-2 mL (min.0.5 mL) in cryovials.
Store cryovials at -80°C until the shipment to Turin Laboratory.

SAMPLE FOR PBMC COLLECTION

1. For each scheduled timing, 2 full 8 mL (16 mL total) Cell Preparation Tubes (CPT ® Vacutainers; Becton, Dickinson and Co., Franklin Lakes, NJ, U.S.A) will be collected.

2. The CPT will be immediately inverted 3-4 times in order to achieve tubes activation and brought to the laboratory for the processing. THIS STEP HAS TO BE PERFORMED AT ROOM TEMPERATURE and should be performed within 1 hour.

PBMC ISOLATION PROTOCOL
STARTING NOTE:
The whole protocol should be completed in about 40 minutes after the arrival of CPT tubes to the

Lab. Performing at least a couple of trials is strongly suggested for the technicians involved in the protocol, before applying it to the study.

For each scheduled timing, 2 full 8 mL (16 mL total) Cell Preparation Tubes (CPT ® Vacutainers; Becton, Dickinson and Co., Franklin Lakes, NJ, U.S.A) will be collected.

The CPT will be immediately inverted 3-4 times in order to achieve tubes activation and brought to the laboratory for the processing. THIS STEP HAS TO BE PERFORMED AT ROOM TEMPERATURE and should be performed within 1 hour.

CPT Vacutainers will be centrifuged at 1700 x *g* for 15 min AT ROOM TEMPERATURE, in order to achieve cell separation by density gradient.

FIRST WASHING STEP:

The cell layer will be transferred in 50 mL Falcon tubes, brought to 40 mL with cold isotonic solution (0.9% NaCl at 4°C), gently inverted and centrifuged at 700 x *g* for 6 min at 4°C. FROM NOW ON, ALL THE STEPS HAVE TO BE PERFORMED IN COLD MEDIA.

ERYTHROCYTE LYSIS:

After discarding the supernatant, the cell pellet will be treated with 2mL of cold ammonium salt solution (3.5 g ammonium chloride + 0.036 g ammonium carbonate in 500 mL of water at 4°C), in order to break residual erythrocytes' cells membranes, left in incubation **exactly 1 min** and then brought to volume of 40 mL with cold isotonic solution (0.9% NaCl at 4°C), in order to dilute the ammonium salt solution.

CELL COUNTING (if possible):

If an automated counter is available: Two aliquots of 0.5 mL of the cell suspension will be transferred and diluted in 2 beckers with 19.5 mL of isotonic solution, for the cell counting. IMMEDIATELY PERFORM THE SECOND WASHING STEP ON THE RESIDUAL VOLUME. The cell number and the mean cell volume (MCV) will be determined in double replicate for each becker through an automated cell counter (Beckman Z2 Coulter®).

Direct count with Burker's chamber could be alternatively used instead of a cell counter.

SECOND WASHING STEP:

contextually with the cell counting, the remaining 39 mL of the cell suspension (or 40 mL if counting has not been performed) will be centrifuged again at 700 x *g* for 6 min at 4°C. Immediately discard the supernatant.

CELL STORAGE:

The resulting pellet (residual volume about 100 µL) will be added with 900 µL of water: methanol

30:70 vol:vol solution, resuspended and divided in two aliquots (about 500 µL each).
Storage should be carried out immediately at -80°C.

Note: *if cell counting with an automated counter is not possible (this is the first choice in terms of accuracy), the counting will be performed with a turbidimetric assay at the Lab. of Clinical Pharmacology and Pharmacogenetics.*
Generally, a good number of PBMC for each aliquot is around 1 or 2x10^6, with an optimal number around 5x10^6.

Additional Note: *for security and sample integrity reasons, the use of Cryovials with an internal screw cap should be strongly discouraged. External-screw caps are the best options in order to avoid sample leakage.*
